# On-demand utilization of phosphoribosyl pyrophosphate by downstream anabolic pathways

**DOI:** 10.1016/j.jbc.2023.105011

**Published:** 2023-07-04

**Authors:** Benoît Pinson, Michel Moenner, Christelle Saint-Marc, Alexandra Granger-Farbos, Bertrand Daignan-Fornier

**Affiliations:** 1 Institut de Biochimie et Génétique Cellulaires, UMR 5095, CNRS – Université de Bordeaux, Bordeaux, France; 2 Metabolic Analyse Service, TBMCore – Université de Bordeaux - CNRS UAR 3427 - INSERM US005, Bordeaux, France

**Keywords:** pentose phosphate pathway, nucleotide biosynthesis, phosphoribosyl pyrophosphate, yeast

## Abstract

The pentose phosphate pathway (PPP) is critical for anabolism and biomass production. Here we show that the essential function of PPP in yeast is the synthesis of phosphoribosyl pyrophosphate (PRPP) catalyzed by PRPP-synthetase. Using combinations of yeast mutants, we found that a mildly decreased synthesis of PRPP affects biomass production, resulting in reduced cell size, while a more severe decrease ends up affecting yeast doubling time. We establish that it is PRPP itself that is limiting in invalid PRPP-synthetase mutants and that the resulting metabolic and growth defect can be bypassed by proper supplementation of the medium with ribose-containing precursors or by the expression of bacterial or human PRPP-synthetase. In addition, using documented pathologic human hyperactive forms of PRPP-synthetase, we show that intracellular PRPP as well as its derived products can be increased in both human and yeast cells, and we describe the ensuing metabolic and physiological consequences. Finally, we found that PRPP consumption appears to take place “on demand” by the various PRPP-utilizing pathways, as shown by blocking or increasing the flux in specific PRPP-consuming metabolic routes. Overall, our work reveals important similarities between human and yeast for both synthesis and consumption of PRPP.

The pentose phosphate pathway (PPP; Fig. 1*A*) has two key functions (1). At first, it contributes to a large part of NADP reduction through its oxidative branch and, second, it provides ribose-5-phosphate, an obligatory precursor of the synthesis of all nucleotides and derivatives, through the non-oxidative branch. In addition, the PPP produces C3 to C7 metabolites, of which erythrose-4-phosphate is used in the synthesis of aromatic amino acids. Hence, the PPP is central to anabolism, notably through the synthesis of pentose for nucleotides and the supply of NADP(H) which is involved in most anabolic oxydo-reductive reactions. Therefore, under proliferation conditions, a portion of the carbon flux is routed to PPP and used for anabolism and *de novo* biomass formation.

**Figure 1:**
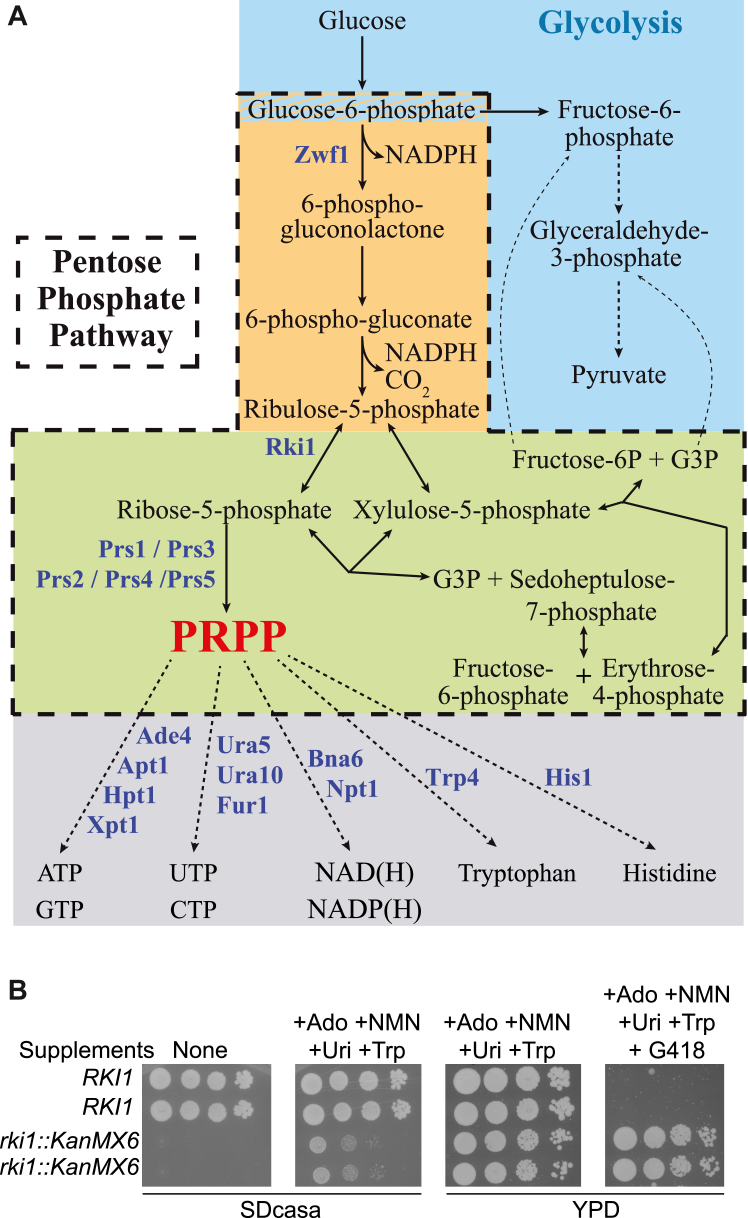
**Schematic representation of PRPP synthesis and consumption in *Saccharomyces cerevisiae***. *A*, Proteins mentioned in the text are shown in *blue*. Glycolysis, pentose phosphate pathway oxidative and non-oxidative branches, and PRPP consumption pathways are boxed in *blue*, *orange*, *green*, and *gray*, respectively. *B*, essential synthesis of ribose-5-phosphate by the ribose-5-phosphate transketolase Rki1 can be rescued by supplementation bypassing PRPP requirement. The heterozygote diploid *RKI1*/*rki1*Δ (Y5217) was transformed with a plasmid allowing expression of the human nucleoside carrier hENT1 (p4491). Transformants were sporulated, and the meiotic progeny was dissected by micromanipulation on YPDA medium supplemented with Adenosine (Ado), Uridine (Uri), and Tryptophan at 300 μM each and Nicotinamide mononucleotide (NMN) at 100 μM. Serial dilutions (1/10) of four spores from a representative tetrad were spotted on indicated media, and plates were imaged after 2 days at 30 °C. ATP, adenosine triphosphate; CTP, Cytidine triphosphate; G3P, glyceraldehyde-3-phosphate; GTP, Guanosine triphosphate; NAD(H), Nicotinamide adenine dinucleotide (reduced form); NADP(H), Nicotinamide adenine dinucleotide phosphate (reduced form); PRPP, 5-phosphoribosyl-1-pyrophosphate and UTP, Uridine triphosphate.

Synthesis of NADPH by the PPP is particularly important for human erythrocytes and a defect in glucose-6-phosphate dehydrogenase (G6PDH), the first step of the oxidative branch of PPP from glucose-6-phosphate, results in hemolytic anemia (2). In yeast cells, NADPH synthesis *via* the PPP is also important but essentially under oxidative stress conditions or when a significant amount of NADPH is needed for sulfur assimilation. Accordingly, the yeast *zwf1* mutant lacking G6PDH activity is hyper-sensitive to oxidizing agents and is an auxotroph for methionine (3). Importantly, while most of the genes encoding enzymes of the PPP are not essential in yeast (4, 5, 6, 7, 8), *RKI1* encoding ribose-5-phosphate ketol-isomerase is indispensable for yeast survival (9), presumably due to its critical role in synthesizing ribose-5-phosphate, which is an essential metabolite required for nucleotide biosynthesis. In fact, ribose-5-phosphate is never used as such in biosynthesis reactions; it first has to be activated to phosphoribosyl pyrophosphate (PRPP), the final product of PPP, by a family of enzymes named PRPP-synthetases (Fig. 1*A*).

In budding yeast, PRPP-synthetases are multimeric enzymes. There are five genes, *PRS1-5*, encoding PRPP synthetase subunits. Based on specific activity measurements from single and combined knockout (KO) (10, 11), as well as single and combined expression of the yeast subunits in *E. coli* (12), it was concluded that Prs1 with Prs3 form the major enzyme complex, while Prs2-Prs5 and Prs4-Prs5 form two minor complexes. Of note, Prs1 and Prs5 carry additional non-homologous regions which interact with the cell wall integrity pathway (13, 14). Importantly, *prs1* and *prs3* individual KO resulted in a severe growth defect while only combinations of KO comprising members of both the Prs1-Prs3 and the Prs2-Prs5 complexes were synthetic lethal (10). Finally, *prs1* and *prs3* mutants had low intracellular nucleotides (10). However, it is not yet established whether the slow growth of these mutants is the direct consequence of limiting amounts of PRPP or whether there are unrelated effects of the deletion of the *PRS1* or *PRS3* genes. In yeast, PRPP can be used in enzymatic reactions, in five different synthesis pathways, catalyzed by 11 phosphoribosyl transferases (Fig. 1*A*) (15): purine nucleotide biosynthesis (Ade4, Apt1, Hpt1, Xpt1); pyrimidine nucleotide biosynthesis (Ura5, Ura10, Fur1); pyridine cofactor biosynthesis (Bna6, Npt1); histidine biosynthesis (His1); and tryptophan biosynthesis (Trp4). Whether some of these pathways are more limiting than others, when PRPP synthesis is low, is not known.

In humans, there are three monomeric PRPP synthetase isoforms (PRPS1-3). The PRPS1 and PRPS2 enzymes are 95% identical and are expressed in all tissues examined, while PRPS3 is specifically expressed in the testis (16, 17). PRPS1 is the most highly expressed isoform and is mutated in a whole range of genetic diseases (18). Interestingly, several distinct syndromes are associated with specific mutations in PRPS1, either decreasing or increasing its activity (18). It hence appears that in humans, the level of PRPP synthetase must be finely tuned to a given physiological level. In addition, several lines of evidence support the central role of PRPS1 and PRPS2 in tumor progression and resistance to anti-cancer drugs. Increased activity of PRPS1 was shown responsible for thiopurine resistance in relapsed childhood Acute Lymphoblastic Leukemia (ALL) (19), as well as cisplatin resistance in breast cancer cells (20). Besides the drug resistance effects, increased *PRPS1* expression was found to result in an anti-apoptotic effect in B-ALL cell lines and was associated with a poor prognosis for disease progression (21). Finally, activation of PRPS1 by keto hexokinase-A drives hepatocellular carcinoma formation (22). PRPS2, although it is dispensable in mice, is required for Myc-driven tumorigenesis (23) leading to an interesting alternative strategy to indirectly target the “undruggable” Myc protein. More recently, PRPS2 overexpression was found to increase cell migration and invasion and proposed to drive colorectal cancer metastasis (24). The requirement for PRPP for all the nucleotide synthesis pathways makes the synthesis of PRPP an interesting metabolic bottleneck that could be targeted. PRPS1 and PRPS2 are thus generating growing attention for their roles in cancer cells. However, the physiological fine-tuning of PRPS activity, affected in several diseases, makes it challenging to target PRPS. Therefore, a better understanding of PRPP synthesis and utilization is required.

In this work, we first established that the synthesis of ribose-5-phosphate and PRPP is the essential function of PPP in yeast. We then aimed at deciphering how PRPP synthesis and consumption affect metabolism and proliferation in yeast cells. We report that PRPP synthetase, but not ribose-5-phosphate, is limiting PRPP synthesis. We also show that PRPP-utilizing enzymes compete for PRPP and that, under physiological conditions, PRPP is limiting for nucleotide synthesis but not for proliferation and that it is competitively consumed by the downstream pathways. Finally, we investigated PRPP metabolism in human cells and found that the main conclusions drawn from yeast apply quite similarly to human cells.

## Results

### Synthesis of ribose-5-phosphate and PRPP is the essential function of PPP in yeast

As mentioned above, *RKI1* is the only essential gene in the PPP. This is most likely due to the fact that the PPP can be fed by the glycolysis pathway *via* several intermediates, namely, glucose-6-phosphate, fructose-6-phosphate, and glyceraldehyde-3-phosphate (Fig. 1*A*). It ensues that there is a redundancy in the synthesis of PPP intermediates with a possible exception for ribose-5-phosphate synthesized by Rki1 and for its derived product, PRPP, synthesized by Prs1-5 which are also essential for yeast cells in combinations (10). We thus hypothesized that the lethality associated with *rki1* knock-out should be bypassed if essential metabolites requiring PRPP for their synthesis were provided as supplements to the growth medium. These metabolites are purines, pyrimidines, pyridines, and amino acids (Fig. 1*B*). The supplementation could not simply be done by feeding the cells with preformed nucleotides since nucleotides are not taken up by yeast cells. We hence fed the cells with nucleosides, which are nucleotide precursors carrying the ribose moiety but no phosphate. The nucleosides provided to cells were adenosine, uridine, and nicotinamide riboside (Nr) for purines, pyrimidines, and pyridines, respectively. Of note, since Nr is chemically unstable, cells were fed with nicotinamide mononucleotide that is metabolized to Nr by the Pho5 phosphatase enzymatic activity and immediately taken up by the Nrt1 permease, as shown previously (25). Since adenosine is not taken up efficiently in *Saccharomyces cerevisiae*, we expressed a human nucleoside transporter (hENT1) to facilitate uptake. In addition, tryptophan and histidine were supplied to bypass the requirement of PRPP for the synthesis of these amino acids. This combination of nucleoside and amino-acid supplements clearly bypassed the inviable phenotype of the *rki1* mutant (Fig. 1*B*) lacking ribose-5-phosphate ketol-isomerase and hence unable to synthesize ribose-5-phosphate from ribulose-5-phosphate (Fig. 1*A*). We conclude that the main function of the non-oxidative branch of the PPP, in yeast, is to provide ribose-5-phosphate that is used for nucleotide and amino acid synthesis *via* the synthesis of PRPP by Prs1-5. PRPP synthesis from ribose-5-phosphate hence appears to be the essential role of the PPP in yeast.

### Metabolic and phenotypic effects of alterations in the yeast PRPP synthetase genes

**Figure 2:**
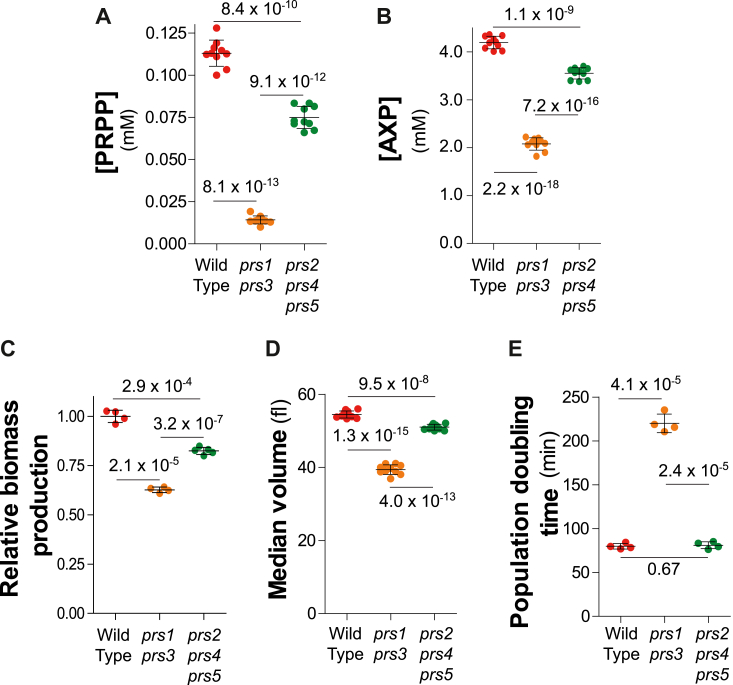
**Phenotypic and metabolic consequences of PRPP decrease in yeast**. *A*–*E*, Wild-type (Y12325), *prs1*Δ *prs3*Δ (Y12562), and *prs2*Δ *prs4*Δ *prs5*Δ (Y12649) were exponentially grown in SDcasaWAU medium for 24 h before measurement of biomass production rate (*C*), median volume (*D*), population doubling time (*E*), or metabolic extraction, separation and quantification (*A* and *B*), done as described in Experimental procedures. [AXP] = [ATP] + [ADP]+ [AMP] (individually presented in Fig. S1, *A*, *E*, and *I*). Each parameter was determined on four to eight independent measurements. Numbers on each panels correspond to *p*-values calculated from a Welch’s unpaired *t* test.

We then evaluated the metabolic and phenotypic contributions of the various yeast PRPP-synthetase isoforms in an isogenic prototrophic background. Prototrophic strains were used to ensure that the observed effects did not result from metabolic interferences with the auxotrophic markers commonly used to facilitate genetic studies in yeast and which often affect PRPP-requiring pathways. Two mutant strains lacking either the major enzyme (*prs1 prs3* double knock-out mutant) or the minor forms (*prs2 prs4 prs5* triple knock-out mutant) were compared with an isogenic wild-type strain. Intracellular PRPP was in the 0.1 mM range for the wild-type strain, lowered by 25% in the *prs2 prs4 prs5* triple mutant, and was decreased almost 10-fold in the *prs1 prs3* mutant (Fig. 2*A*). This result is in good agreement with previous results showing a much more severe defect in PRPP synthetase activity in the *prs1 prs3* mutant than in the *prs2 prs4 prs5* triple mutant (10). Of note, the concentration of PRPP in yeast cells is way below the concentration of nucleotides which is around 4 mM for the sum of adenylic nucleotides (AXP) alone (Fig. 2*B*). This suggests that PRPP is not stored but rather utilized on demand.

**Figure 3:**
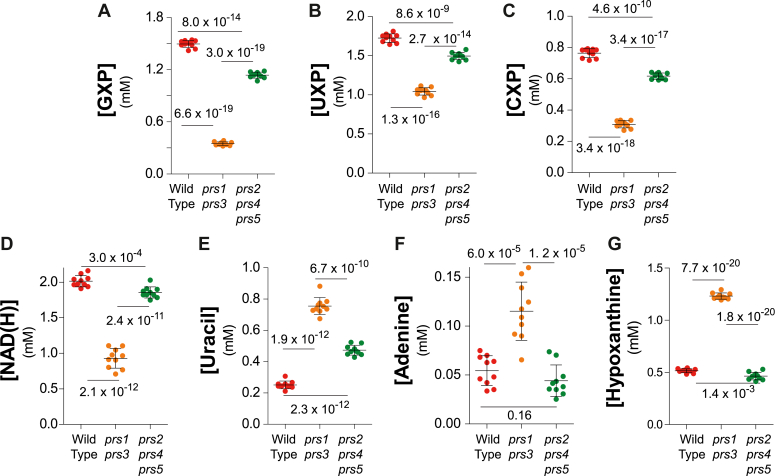
**Consequences of PRPP variations on nucleotide synthesis and precursor accumulation**. Metabolic extractions and quantification were performed on wild-type (Y12325), *prs1*Δ *prs3*Δ (Y12562), and *prs2*Δ *prs4*Δ *prs5*Δ (Y12649) exponentially grown for 24 h in SDcasaWAU medium, as in Figure 2. For each nucleotide (N), [NXP] = [NTP] + [NDP]+ [NMP] (presented in Fig. S1). NAD(H) corresponds to the sum of the oxidized (NAD^+^) and reduced (NADH) form of the pyridine nucleotide. Each metabolite, nucleotide (*A*–*D*), and precursor (*E*–*G*) was quantified from eight independent yeast cultures and metabolite extractions. Numbers on each panel correspond to *p*-values calculated from Welch’s unpaired *t* test.

The effects of the mutations on intracellular PRPP (Fig. 2*A*) were associated with a significant decrease in the ability of the mutant cells to produce biomass, although the effect was much stronger in the case of the *prs1 prs3* mutant (Fig. 2*C*). Quantifying biomass production rate does not allow, in a single measurement, to differentiate the effects on cell division from those on cell growth. We hence appraised these two parameters separately, by measuring cell volume (Fig. 2*D*) and population doubling time (Fig. 2*E*). Clearly, in the triple *prs2 prs4 prs5* mutant, the lower biomass production was associated with a lower cell volume, while doubling time was not increased (Fig. 2, *D* and *E*). By contrast, in the *prs1 prs3* mutant, both factors were severely affected (Fig. 2, *D* and *E*). Finally, metabolic profiling of the three strains revealed that the *prs1 prs3* mutant was the most affected strain for all four nucleotides as well as the sum of NAD^+^ and NADH (hereafter referred as NAD(H)), while the *prs2 prs4 prs5* mutant was less severely, although significantly, affected (Figs. 2*B*, 3, *A*–*E*, and S1). Of note, by contrast to previously published data (10), we found that AXP, GXP, UXP, and CXP were all significantly affected in the *prs2 prs4 prs5* mutant when compared to the isogenic wild-type strain (Figs. 2*B* and 3, *A*–*C*). This discrepancy could be due to the fact that the strains used in our work are prototrophic, while in the previous work, the used strains were mutated in four PRPP-utilizing pathways (purine, pyrimidine, tryptophan, and histidine). Interestingly, uracil, adenine and hypoxanthine, three pyrimidine and purine precursors, were higher in the *prs1 prs3* mutant than in the control strain (Fig. 3, *E*–*G*), suggesting that the low PRPP level in the mutant limited their consumption by the cognate phosphoribosyl transferases (Fig. 1*A*).

We conclude that decreasing intracellular PRPP (Fig. 2*A*), and subsequently intracellular nucleotides (Figs. 2*B* and 3, *A*–*D*), severely affected biomass production rate in both *prs* mutants (Fig. 2*C*), while division, as revealed by population doubling time, was only affected in the *prs1prs3* mutant but not significantly in the *prs2 prs4 prs5* mutant (Fig. 2*E*). Hence, PRPP shortage, apparently, first affected cell growth (size decrease, Fig. 2*D*) before slowing down cell division (doubling time increase, Fig. 2*E*).

**Figure 4:**
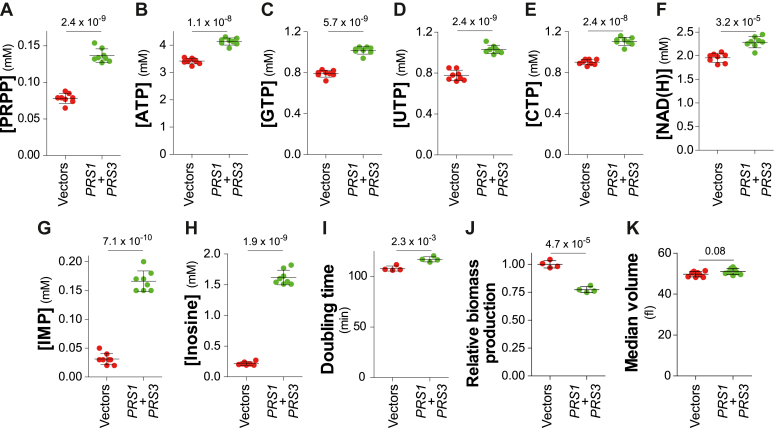
**Metabolic and phenotypic consequences of *PRS1-PRS3* overexpression.** Wild-type (Y11451) strain was transformed with either the empty vectors (vectors, YepLac181 and YepLac195) or plasmids allowing overexpression of *PRS1* (p6160) and *PRPS3* (p5671) (*PRS1* + *PRS3*). Transformants selected on SC -Ura -Leu medium were exponentially grown for 24 h in the same liquid medium before extractions and quantification of metabolites from eight independent cultures (*A*–*H*) or determination of either the population doubling time (*I*), the relative biomass production rate (*J*) or the median volume (*K*). The *p*-values, calculated from Welch’s unpaired *t* test are presented by numbers.

We then overexpressed the two major isoform genes (*PRS1* and *PRS3*) concomitantly on multicopy yeast plasmids and evaluated the metabolic and phenotypic effects (Fig. 4). Of note, due to the presence of the two plasmids, the yeast strains and the growth media used in Figures 3 and 4 are different. The metabolic effects of *PRS1*/*PRS3* overexpression, increased PRPP (Fig. 4*A*), as well as nucleotide and NAD(H) concentrations (Fig. 4, *B*–*F*). This result shows that increasing PRPP synthesis in yeast can significantly increase intracellular nucleotides. Accordingly, the purine intermediate metabolites IMP and inosine were also very significantly increased (Fig. 4, *G* and *H*), as previously reported for purine overproducing *ADE4* dominant mutants (25). However, nucleotide overproduction did not improve yeast growth and division. Indeed, when considering the physiological consequences of *PRS1*/*PRS3* overexpression, we observed that it negatively increased the population doubling time (Fig. 4*I*) and diminished the biomass production rate (Fig. 4*J*), while it had no significant effect on cell size (Fig. 4*K*). This establishes that there is no strict proportional relationship between PRPP concentration and generation time or cell size and that the connection between metabolism and these traits is probably more complex. The metabolic burden imposed by increased synthesis of nucleotides and accumulation of intermediates such as IMP and inosine in the mM range (Fig. 4, *G* and *H*) could be responsible for the higher doubling time by diverting a significant part of glucose from glycolysis to nucleotide synthesis *via* the PPP.

### Phenotypic and metabolic rescue of the yeast prs1prs3 mutant by exogenous supplementation or expression of human and bacterial PRPS genes

It should be stressed that the results on doubling time and biomass do not allow us to conclude whether these effects are consequences of the decreased intracellular nucleotide levels. It could in part be due to lower intracellular PRPP, independent of its effect on nucleotides, or alternatively to a possible moonlighting role of PRPS independently of its catalytic activity.

**Figure 5:**
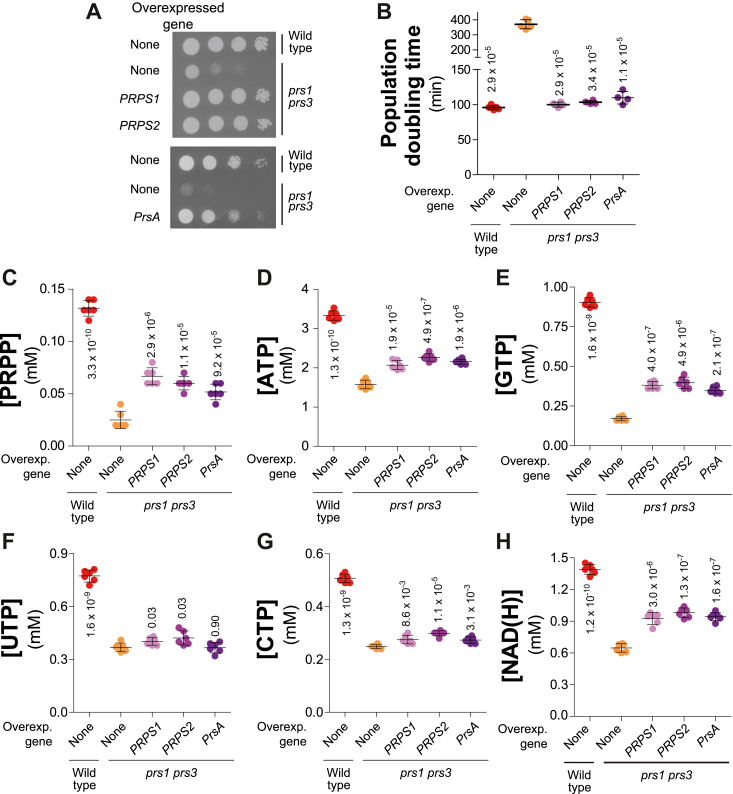
**PRPP content is limiting for yeast proliferation and nucleotide synthesis.***A*, the growth defect of the *prs1 prs3* mutant is restored by expression of either the human or bacterial PRPP synthetases. Wild-type (Y11418) and *prs1*Δ *prs3*Δ (Y11983) strains were transformed with either an empty vector (None) or plasmids allowing expression of human (*PRPS1* (p6001) or *PRPS2* (p6020)) or bacterial (*PrsA;* p5909) PRPP-synthetases. Transformants were selected on SDcasaWA medium and serial dilutions (1/10) of transformants were spotted on SDcasaWA and plates were imaged after 36 h (*PrsA*) or 48 h (*PRPS*) at 30 °C. *B*–*I*, transformants were exponentially grown for 24 h in liquid SDcasaWA before determination of the population doubling time (*B*) or extractions and quantification of metabolites (*C*–*H*). Each metabolite was quantified from six independent cultures. The *p*-values, calculated from Welch’s unpaired *t* test, are presented by numbers. Overexp., overexpressed.

First, to establish that PRPP synthesis is indeed the limiting factor for proliferation in the *prs1 prs3* mutant, we expressed the monomeric human PRPP synthetases, PRPS1 and PRPS2, as well as the *E. coli* enzyme (PrsA) under the control of a yeast promoter. Expression of the human or bacterial enzymes restored colony growth on plates (Fig. 5*A*) and doubling time in liquid cultures (Fig. 5*B*). Importantly, expression of the exogenous PRPS in yeast restored intracellular PRPP, although only partially (Fig. 5*C*). Similarly, expression of exogenous PRPS in yeast only partially, but significantly, restored nucleotide levels, although to various extents, higher for purines than for pyrimidines for unknown reasons (Fig. 5, *D*–*H*). We conclude that synthesis of PRPP derivatives is apparently limiting for proliferation in the *prs1 prs3* mutant and that partial restoration of the intracellular pools, by expression of exogenous PRPS, is likely sufficient to recover doubling times not very different from that of the wild-type control. Of note, this result is highly consistent with the one observed previously for the *prs2 prs4 prs5* mutant (Fig. 2*E*). It should be noted that the phenotypic and metabolic rescue by expression of bacterial PrsA or human PRPS in the *prs1 prs3* mutant is probably contributed, in part, by the Prs2 Prs4 and Prs5 isoforms that are still present in this mutant (11).

**Figure 6:**
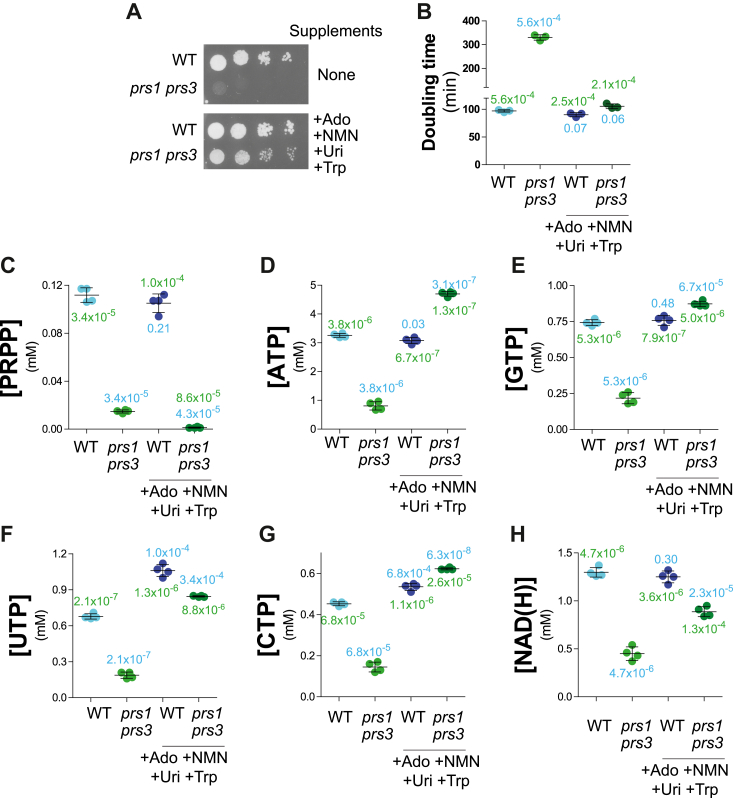
**Growth and metabolic defects of the *prs1 prs3* mutant are rescued by supplementation with PRPP-not-requiring precursors.***A*, addition of nucleoside precursors partially restores the *prs1 prs3* mutant growth defect. Wild-type (Y11418) and *prs1*Δ *prs3*Δ (Y11985) strains were transformed with the h*ENT1* expressing plasmid (p4991). Transformants were selected on SDcasaW medium supplemented with adenosine (Ado, 300 μM), Uridine (Uri, 300 μM), Tryptophan (300 μM), and Nicotinamide mononucleotide (NMN, 100 μM). Transformants were serial diluted (1/10) and spotted on SDcasaW supplemented or not with Ado, Uri, Trp, and NMN. Plates were imaged after 36 h at 30 °C. *B*-*H*, transformants were exponentially grown for 24 h in SDcasaW liquid medium supplemented or not with the indicated precursors prior to population doubling time measurement (*B*) or metabolic extraction, separation, and quantification (*C*–*H*). The *p* values, calculated from a Welch’s unpaired *t* test, are presented by either blue (comparison with wild-type cells grown without precursors) or *green numbers* (comparison with *prs1*Δ *prs3*Δ cells grown without precursors).

We then asked whether it is PRPP itself or its derived products that is limiting the growth of the *prs1 prs3* mutant. We intended to address this question by restoring higher intracellular nucleotides without restoring PRPP-synthetase activity. This was done as above for *rki1* by feeding the cells with nucleosides and amino acids. Of note, histidine is contained in the casamino mixture added to the medium and is present in all conditions. The addition of each alone of the ribose-containing precursors or final products had little effect on the colony growth of a *prs3* mutant (Fig. S2); however, the combined addition of the supplements resulted in a significant improvement in colony growth (Fig. 6*A*). Furthermore, the rescue of doubling time by the combination of supplements was total in liquid cultures (Fig. 6*B*), suggesting that the partial rescue effect observed on plates (Fig. 6*A*) could be due to poor diffusion of the substrates in the agar plate, as previously reported (26). Importantly, the addition of the supplements to the *prs1 prs3* mutant did not increase intracellular PRPP concentration (Fig. 6*C*) but rather lowered it for a yet ununderstood reason, while, as expected, it significantly increased all intracellular NTPs (Fig. 6, *D*–*G*) to levels comparable to those of the wild-type isogenic strain. Meanwhile, NAD(H) was only partially restored (Fig. 6*H*), the metabolization of nicotinamide mononucleotide to Nr, and/or the uptake of Nr by Nrt1 being possibly limiting. We conclude that most of the growth defect of the *prs1 prs3* mutant is due to its inability to synthesize a sufficient amount of PRPP-derived metabolites.

Altogether, our results show that low synthesis of PRPP and more specifically of its derived products, such as nucleotides, is the principal cause of the slow growth phenotype of the *prs1 prs3* mutant.

### Isolation and characterization of genetic suppressors of the prs1 prs3 mutant

**Figure 7:**
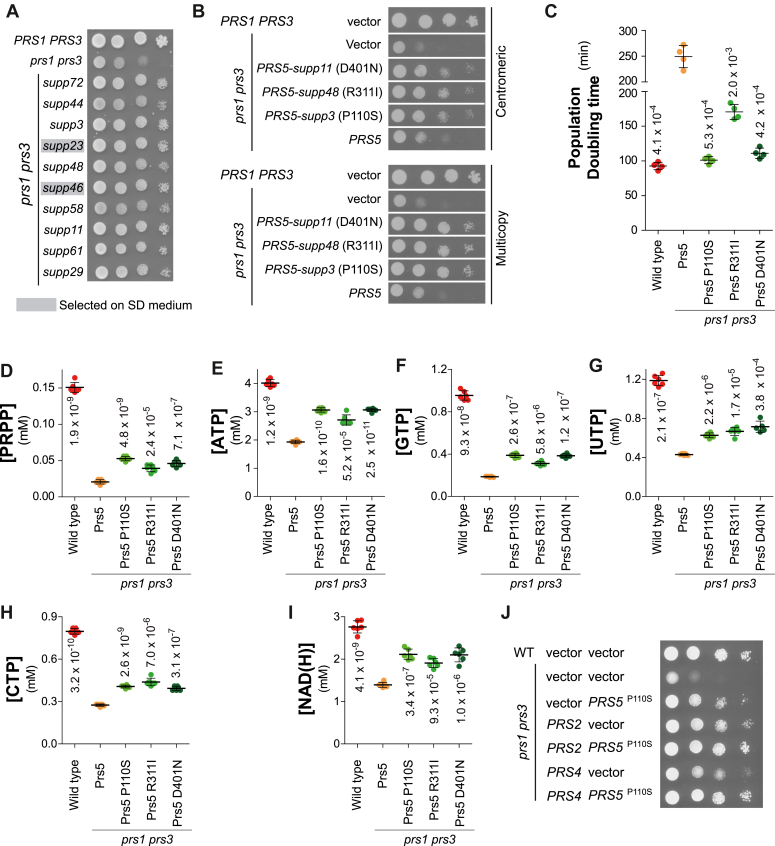
**Suppression of *prs1 prs3* growth and metabolic defects by mutations in the *PRS5* gene.***A*, suppressors were isolated from *prs1*Δ *prs3*Δ strains as described in the Experimental procedure section. Wild-type (FY4), *prs1*Δ *prs3*Δ (Y12562), and suppressors strains (Table S1) were serial diluted (1/10), spotted on SDcasaWAU medium, and imaged after 48 h at 30 °C. *B*, plasmid-driven expression of *PRS5* suppressor alleles is sufficient to alleviate the *prs1*Δ *prs3*Δ growth defect. Wild-type (Y11418) and *prs1*Δ *prs3* (Y11983) strains were transformed with empty vectors (YCplac33 (top) or YEplac195 (bottom)), or plasmids expressing the *PRS5* gene either wild-type (p6017 or p6019) or suppressing alleles (*supp11* (p6009 or p 6008); *supp48* (p6014 or p 6011) and *supp3* (p6016 or p 6015)). Transformants were serial diluted (1/10), spotted on SDcasaW medium, and imaged after 48 h at 30 °C. *C*-*I*, Wild-type (FY4) and *prs1*Δ *prs3*Δ strains expressing the *PRS5* gene either wild-type (Y12563) or mutated ((Y12214; Y12271; Y12272) were exponentially grown for 24 h in SDcasaWAU before determination of population doubling time (*C*) or nucleotides content (*D*–*I*). Numbers correspond to the *p*-values calculated from Welch’s unpaired *t* test. *J*, overexpression of *PRS5* in combination with either *PRS2* or *PRS4* is sufficient to restore a wild-type growth in *prs1*Δ *prs3*Δ genetic background. Wild-type (BY4741) or *prs1*Δ *prs3*Δ (Y2850) were co-transformed with empty vectors (Yeplac195; YEplac181) or plasmids expressing *PRS2* (p6070), *PRS4* (p6094) or *PRS5*^*P110S*^ (p6015). Transformants, selected on SC-Ura-Leu were serial diluted (1/10), spotted on the same medium and imaged after 48 h at 30 °C.

Having shown that supplementation or exogenous PRPS activity could rescue the *prs1prs3* mutant slow growth, we then asked whether this proliferation phenotype could be suppressed genetically. Starting from a *prs1 prs3* double KO mutant, we searched for suppressors that would alleviate the severe colony growth defect of the mutant and thereby possibly reveal metabolic processes limiting proliferation. Because PRPP is a central molecule in metabolism, the experiment was done on prototrophic yeast to avoid interference with auxotrophy markers. After UV mutagenesis, rapid growers were isolated from the *prs1 prs3* slow growers and further characterized. The experiment was run in parallel on “rich” (SDcasaWAU, containing amino-acids and nucleobases) and “poor” (SD, lacking amino-acids and nucleobases) media to establish whether different types of suppressors would be found when the salvage or *de novo* pathways are mostly challenged (Fig. 1), respectively. A total of 69 and 76 suppressors were respectively isolated from SD and SDcasaWAU medium, growth phenotypes of a subset of these mutants are shown in Figure 7*A*. Two mutants (supp3 and supp48) were backcrossed to a *prs1 prs3* parental strain and in both cases, the suppressor mutation was found to segregate as a single locus in the meiotic progeny. Sets of pooled segregants (27) carrying or not the suppressors were sequenced and, for both suppressors, a mutation at the *PRS5* locus was found in the suppressed segregant pools but not in the non-suppressed segregant pools. The *PRS5-C328T* (Pro110Ser) and *PRS5-G932T* (Arg311Ile) mutations were confirmed by resequencing the *PRS5* locus in the original supp3 and supp48 mutants, respectively, while none of these changes were found in the parental strains. Sequencing of the *PRS5* locus from 15 suppressor mutants obtained from SD medium and 15 from SDcasaWAU medium revealed that they all carry a mutation in *PRS5* (Table S1). Finally, the *PRS5-supp3*, *PRS5-supp11* and *PRS5-supp48* mutant alleles suppressed *prs1 prs3* when carried on either centromeric or multicopy plasmids, while the wild-type allele did not (Fig. 7*B*), further confirming that they are necessary and sufficient suppressor mutations and revealing their dominance over the wild-type *PRS5* allele. We conclude that several mutations in *PRS5* can efficiently suppress the growth defect of the *prs1 prs3* double mutant. All the tested *PRS5* suppressor mutations were found dominant. A subset of three suppressors (P110S, R311I, and D401N) was further analyzed revealing that the *PRS5* suppressors decreased the population doubling time (Fig. 7*C*) and had a significant effect on intracellular PRPP (Fig. 7) as well as nucleotide content (Fig. 7, *E*–*I*). Finally, we observed that overexpression of the mutated forms of *PRS5* on multicopy plasmids only slightly improved suppression compared to that obtained with centromeric plasmids (Fig. 7*B*). We hypothesized that this could be due to the limitation of one or both of Prs5 partners, namely, Prs2 and/or Prs4. We hence overexpressed *PRS2* or *PRS4* together with *PRS5*^*P110S*^ and found that indeed the co-overexpression of either *PRS2* or *PRS4* strongly stimulated growth (Fig. 7*J*), indicating that the *PRS5* suppressor acts through its interaction with its partners Prs2 or Prs4. Hence, suppressors of *prs1 prs3* most likely act by increasing PRPP synthesis by the Prs2/4/5 enzyme rather than modulating downstream consumption.

The results presented in this section and the previous one allow us to conclude that in the *prs1 prs3* mutant, PRPP is limiting for proliferation and that these effects can be alleviated by increasing PRPP (through *PRPS1*, *PRPS2* or *PrsA* expression or *PRS5* suppressors) or by bypassing its requirement (*via* supplementation with nucleosides and amino acids).

### PRPP is limiting for nucleotide synthesis but not for proliferation in wild-type yeast

**Figure 8:**
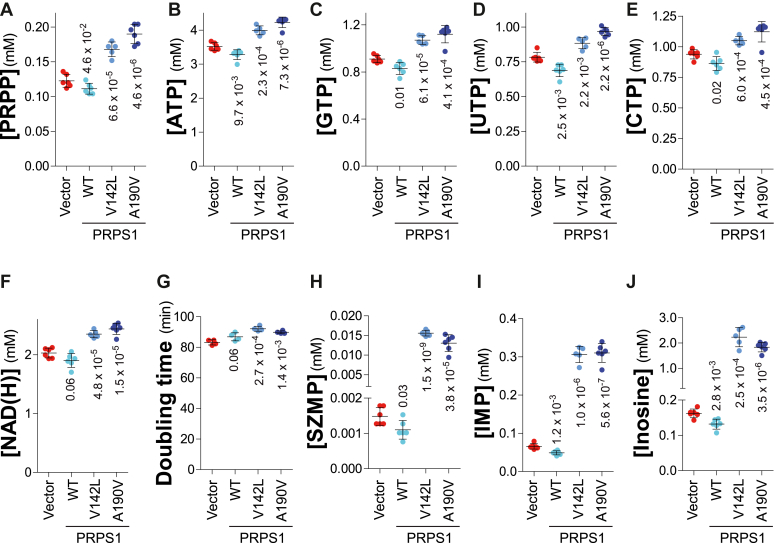
**PRPP and nucleotide content is increased by the expression of human *PRPS1* hyperactive variants.***A–J,* the wild-type strain (Y12599) was transformed with an empty vector (p2714) or plasmids expressing the *PRPS1* gene either wild-type (p5949) or hyperactive mutants (P5994; p5995). Transformants were exponentially grown for 24 h in SDcasaWA liquid medium before metabolite extraction and quantification. Each metabolite was quantified on at least five independent cultures. Numbers correspond to the *p*-values calculated from Welch’s unpaired *t* test.

We then asked whether PRPP is present in excess in yeast cells or whether it could be limiting for nucleotide synthesis even in wild-type cells. To address this question, we first expressed in wild-type yeast cells the *PRPS1* human enzyme that was able to suppress the growth defect of the *prs1 prs3* mutant (Fig. 5*A*). Expression of the human enzyme did not increase intracellular PRPP nor NTPs or NAD(H) in the wild-type cells (Fig. 8, *A*–*F*). This result suggested that ribose-5-phosphate, the substrate of PRPP-synthetase, could be limiting for the reaction to proceed or, alternatively, that the human enzyme is feedback inhibited by some end-product(s) (16). To settle this question, we expressed in yeast two previously described hyperactive variants of PRPS1 (V142L and A190V) (28). Clearly, the expression of these two PRPS1 mutants resulted in a very significant increase of intracellular PRPP (Fig. 8*A*) as well as of all NTPs and NAD(H) when expressed in wild-type yeast (Fig. 8, *B*–*F*). We conclude that PRPP-synthetase activity, rather than the ribose-5-phosphate substrate, is limiting PRPP synthesis and downstream reactions in a wild-type prototroph yeast strain. Furthermore, the expression of these hyperactive forms of PRPP-synthetase significantly affected the doubling time (Fig. 8*G*). Of note, the accumulation of purine intermediates, such as SZMP, IMP and inosine (Fig. 8, *H*–*J*) when the PRPS1 mutants are expressed in yeast, indicates that the flux in the purine *de novo* pathway is overloaded. This metabolic burden could be responsible for the higher doubling time by diverting a significant part of glucose from glycolysis to the PPP and nucleotide synthesis in the form of an unused excess of purines.

**Figure 9:**
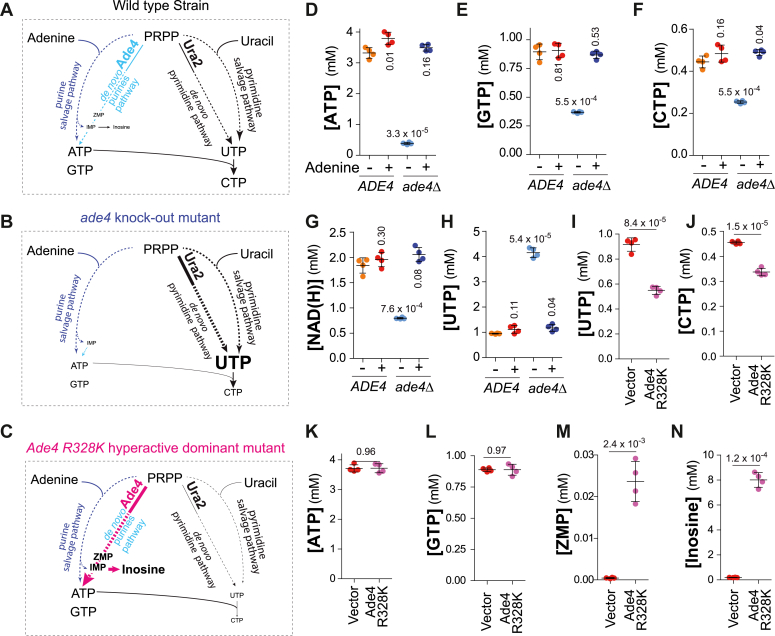
**Impairment of PRPP utilization *via* the purine *de novo* pathway reveals that PRPP is limiting for nucleotide synthesis.***A*, *B*, and *D–H*, Wild-type (FY4) and *ade4*Δ (Y12258) yeast strains were exponentially grown for 24 h in SDcasaWAU liquid medium. Cells were then harvested by filtration and grown for three more hours in SDcasaWU liquid medium supplemented (+) or not (−) with adenine before metabolite extraction, separation, and quantification. *C*, and *I*–*N*, the wild-type strain (Y11418) was transformed with an empty vector (pCM189) or a plasmid carrying the *ADE4* hyperactive R328K dominant mutant (p2048). Transformants were exponentially grown for 24 h in SDcasaWA medium before extraction, separation, and quantification of metabolites. *D–N*, metabolite measurements were performed on four independent cultures and numbers correspond to the *p*-values calculated from a Welch’s unpaired *t* test.

The increase in all NTPs and NAD(H) in yeast strains expressing the two *PRPS1* mutants (Fig. 8, *B*–*F*) suggested that, in wild-type yeast cells, PRPP-utilizing enzymes compete for PRPP as a limiting substrate. To address this question more directly, we asked whether affecting one PRPP-consuming pathway results in an effect on the products of other PRPP-consuming pathways. This was first done by blocking *de novo* synthesis of purine nucleotides, using a *ade4* knockout mutation that blocks the first step of the pathway (Fig. 9, *A* and *B*). This mutant strain can grow and proliferate as long as it is fed with a purine precursor such as adenine, utilizable through the salvage pathway. When shifted for 3 h in a medium lacking adenine, the *ade4* mutant showed a strong decrease of adenylic and guanylic nucleotides (Fig. 9, *D* and *E*) as expected but also CTP and NAD(H) (Fig. 9, *F* and *G*), most probably because their synthesis is affected at low ATP concentration (29, 30). By contrast to CTP, intracellular UTP showed a strong increase, about fourfold, in the *ade4* mutant (Fig. 9*H*), suggesting that the PRPP that was not used for purine, CTP, and pyridine synthesis due to the *ade4* block in the purine pathway could be used to make more UTP. In addition, the expression of an *ADE4* hyperactive dominant allele (Fig. 9, *A* and *C*) (31, 32) resulted in decreased intracellular UTP and CTP (Fig. 9, *I* and *J*). Of note, here UTP and CTP both decreased reflecting the fact that under these experimental conditions, CTP is synthesized *de novo* from UTP. In the same experiment, ATP and GTP were not significantly affected (Fig. 9, *K* and *L*), while the flux in the *de novo* purine pathway was high as attested by the accumulation of the metabolic intermediate ZMP and of the degradation product inosine (Fig. 9, *M* and *N*). These results indicate that the flux in the purine pathway has a strong impact on intracellular UTP. We conclude that, in wild-type yeast, PRPP-utilizing enzymes are competing for PRPP as a substrate which is limiting for the synthesis of downstream products.

This competition between PRPP-utilizing enzymes was further confirmed using a *ura2* block in the pyrimidine pathway (Fig. S3*A*). When these cells were grown in the absence of uracil, we observed a severe decrease of UTP and CTP and a small but significant increase of intracellular ATP (Fig. S3, *B*–*F*)). Due to the different intracellular abundance of ATP and UTP (3.8 and 1.1 mM, respectively. Fig. 9, *A* and *E*) it could be expected that a variation of intracellular ATP would much more severely affect UTP levels than the reverse. Finally, intracellular NAD(H) was tightly correlated to intracellular ATP as reported before (30), and intracellular CTP was correlated to intracellular ATP and UTP which are both required for its synthesis from UTP by CTP-synthase (suggesting that the K_M_ for ATP of this enzyme might be high).

Altogether, our results allow two conclusions. First, decreasing intracellular PRPP leads to a reduction of all downstream products, while increasing intracellular PRPP had the opposite effect, establishing that in wild-type yeast cells PRPP is limiting nucleotide synthesis. Second, when PRPP is over- or under-used in one pathway, it is compensated by its usage in other PRPP-utilizing pathways, indicating that phosphoribosyl transferases are competing for PRPP. We conclude that, in yeast, PRPP synthesis limits nucleotide synthesis but not cell proliferation. In addition, PRPP utilization appears to directly result from competition between the enzymes of the downstream pathways.

### Metabolic and proliferation consequences of PRPS hyperactivity in human cells

**Figure 10:**
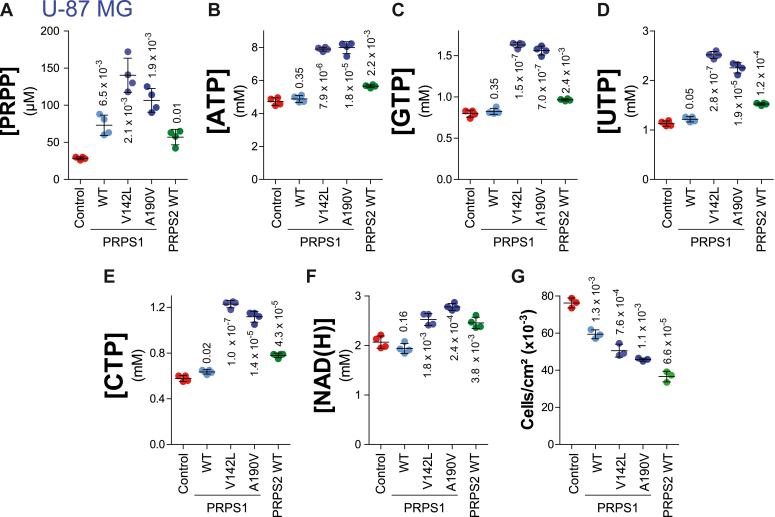
**Expression of human *PRPS1* hyperactive variants in human cells increases PRPP and nucleotide content and affects cell proliferation.** Lentiviral infection was used to express wild-type human *PRPS1* gene (WT, p6033), its two hyperactive mutants (V142L, p6034 and A190V, p6035), or wild-type human *PRPS2* gene (PRPS2 WT, p6030) in U-87 MG human cells. Infection with the pLenti-MND-IRES-PuroR-WPRE (p5769) empty vector was used as control. Metabolite content (*A–F*) was determined as described in “Experimental Procedures” and measurements were performed on four independent extractions. In cell proliferation experiments (*G*), cells were seeded at the density of 7000 cells/cm^2^ and grown for 4 days in a complete medium before trypsinization and counting. Numbers correspond to the *p*-values calculated from Welch’s unpaired *t* test.

Based on the conclusions obtained with yeast cells, we then questioned several aspects of the PRPP metabolism in human cells. We first aimed at evaluating the metabolic consequences of PRPS hyperactivity in the glioblastoma U-87 MG human cell line. *PRPS1* (wild-type, and the hyperactive mutants V142L and A190V) or *PRPS2* were expressed in U-87 MG cells using lentiviruses, and metabolic profiles were established. As in yeast cells, expression of the V142L and A190V *PRPS1* mutants in U-87 MG resulted in a significant increase of PRPP (Fig. 10*A*) as well as of all NTPs, from 1.6-fold for ATP to more than 2-fold for the other NTPs (Fig. 10, *B*–*E*) and of 1.2-fold for NAD(H) (Fig. 10*F*). A significant increase, although slightly lower, was also observed when these mutant PRPS1 alleles were expressed in the colon tumor cell line HCT116 (Fig. S4), indicating that this effect is not cell-line specific. Interestingly, expression of wild-type PRPS1 or PRPS2 increased intracellular PRPP in the U-87 MG cell line (Fig. 10*A*), while such an effect was not observed in the HCT116 cell line (Fig. S4*A*) as well as in yeast cells (Fig. 8*A*), the reasons for this discrepancy are not known. However, while PRPS1 expression had no significant effect on NTPs (Fig. 10, *B*–*E*) or NAD(H) (Fig. 10*F*), expression of PRPS2 resulted in a significant increase of all four NTPs and NAD(H) (Fig. 10, *B*–*F*).

In all experiments, cell proliferation was not accelerated by conditions resulting in higher intracellular NTP content (Fig. 10*G*), indicating that, as observed in yeast, PRPP is limiting for nucleotide synthesis but not for cell proliferation. Actually, in all cases where intracellular NTPs were high, cell proliferation was severely slowed down (Fig. 10*G*), indicating that more NTPs is not “better” for human cells, although the precise reasons for this inhibitory effect are not known.

**Figure 11:**
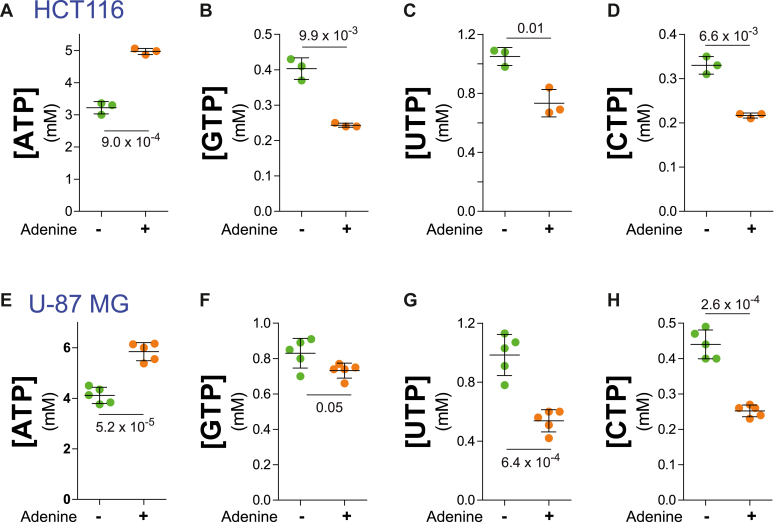
**Addition of a purine precursor reveals that PRPP is limiting for synthesis of nucleotides in human cells.** The human HCT116 (*A–D*) and U-87 MG (*E–H*) cell lines were seeded in 6-well plates and grown up to half-confluency in the complete medium. Cells were then washed and incubated for 2 h in DMEM without serum, and incubated for 24 h in DMEM containing 10% dialyzed serum supplemented (+) or not (−) with 50 μM adenine. Nucleotides content was determined from 3 (*A–D*) to 5 (*E–H*) independent extractions and numbers correspond to the *p*-values calculated from a Welch’s unpaired *t* test.

We then investigated whether increasing the flux in one PRPP consumption pathway affects the others, as observed in yeast cells. This was done by growing the HCT116 or U-87 MG cells in dialyzed serum supplemented or not with adenine, which combined with PRPP can be metabolized to AMP by adenine phosphoribosyl transferase and then to ATP. The addition of adenine indeed increased intracellular ATP (Fig. 11, *A* and *E*) and conjointly decreased all other triphosphate nucleotides (Fig. 11, *B*–*D* and *F*–*H*), suggesting that the PRPP utilized to synthesize ATP from adenine in excess, is limiting the synthesis of other PRPP derived metabolites. We conclude that PRPP utilization in human cells (Figs. 10 and 11), similar to what was obtained in yeast (Fig. 9), is the direct result of a competition between several downstream pathways.

## Discussion

Our work on PRPP synthesis and consumption in yeast gives important clues on upstream and downstream pathways. In particular, it allowed us to establish that ribose-5-phosphate synthesis by Rki1 is essential because of the role of ribose-5-phosphate in PRPP synthesis. Interestingly, this indicates that the alternative synthesis of ribose-5-phosphate from G3P and sedoheptulose-7-phosphate cannot fully compensate for the loss of Rki1 activity to support the growth of yeast cells (Fig. 1).

Clearly, *PRPS1* hyperactive mutants expressed in yeast or human cells increase both intracellular PRPP and downstream products such as nucleotides. These results establish that ribose-5-phosphate, just upstream of PRPP synthesis in the PPP (Fig. 1), is not limiting for PRPP synthesis. From these experiments, we propose that ribose-5-phosphate is always in excess and that the portion not used for PRPP synthesis is recycled in the glycolysis. Accordingly, experiments with wild-type PRPS1 suggest that PRPP synthesis is limited by PRPP synthetase regulation. Indeed, by contrast to the hyperactive mutant forms of PRPS1, expression of wild-type PRPS1 has no effect on the concentration of PRPP (except in the U-87 MG cell line Fig. 10), and no effect either on intracellular nucleotides (Figs. 8, *B*–*F*, 10, *B*–*F*, and S4, *B*–*F*). Importantly, the overproduction of PRPP decreased the proliferation of both the human cells (Figs. 10*G* and S4*G*) and the yeast cells (Fig. 8*G*). The reason for this defect is not established, but it could reflect the metabolic burden associated with the production of PRPP, and most importantly nucleotides, that are not further used for growth but degraded and excreted in the form of inosine in yeast and uric acid in humans.

Additionally, our results in yeast show that PRPP is synthesized at the minimal flow allowing maximal biomass production. Slightly decreasing PRPP concentration (*prs2 prs4 prs5* triple mutant, Fig. 2*A*) results in a lower biomass production rate (Fig. 2*C*), while doubling time is not affected (Fig. 2*E*), indicating that in this condition, maintaining the doubling time is preferred over maintaining cell size. However, further decreasing intracellular PRPP (*prs1 prs3*) double mutant (Fig. 2*A*) affected both biomass production rate (Fig. 2*C*) and population doubling time (Fig. 2*E*). Strikingly, overexpression of *PRS1/PRS3* resulting in increased PRPP concentration also diminished the biomass production rate and increased doubling time (Fig. 4, *F* and *H*). These results parallel the fact that both gain and loss of function, in PRPS1, are associated with human diseases (18). We interpret these results as an indication that, under standard conditions in wild-type yeast, the PRPP concentration is optimal for growth and division and that an increase or decrease of PRPP synthesis capacity affects these parameters (with doubling time taking precedence over cell volume when PRPP is only slightly limiting, see Fig. 2). These results fit well with the central role of PPP in anabolism and with the key role of PRPP as a product of PPP.

Most importantly, PRPP consumption, in yeast and human cells, appears to take place “on demand” by the various PRPP-utilizing pathways. This was shown by blocking or increasing the flux in specific PRPP-consuming metabolic routes (Figs. 9, 11 and S3). The results show that there is apparently no regulation of the utilization of PRPP between the various pathways using it, just competition for a common substrate.

Together, our results show that PRPP metabolism responds to an action mass law where the ribose-5-phosphate provided by the PPP is not limiting for PRPP synthesis and where PRPP utilization is an on-demand process depending most probably on the amount and kinetic parameters of the various involved PRPP-utilizing enzymes.

## Experimental procedures

### Yeast media and strains

SD is a synthetic minimal medium containing 0.5% ammonium sulfate, 0.67% yeast nitrogen base (Difco), 2% glucose. SDcasaW is an SD medium supplemented with 0.2% casamino acids (Difco) and tryptophan (0.2 mM). When indicated, adenine (0.3 mM) and/or uracil (0.3 mM) were added to SDcasaW medium, resulting in a medium named SDcasaWA (+adenine), SDcasaWU (+uracil) and SDcasaWAU (+ adenine + uracil). SC -Ura -Leu medium is SC medium supplemented with adenine (0.3 mM), histidine (0.06 mM), lysine (0.06 mM), and tryptophan (0.2 mM). The YPD medium contained 1% yeast extract, 2% peptone, and 2% glucose. Yeast cells were grown in liquid or solid media at 30 °C. Yeast strains (listed in Table S2) belong to, or are derived from, FY4 and FY5 prototrophic strains (33) or disrupted strains isogenic to BY4741 or BY4742 purchased from Euroscarf (Frankfurt, Germany). Multi-mutant strains were obtained by crossing, sporulation, and micromanipulation of meiosis progeny.

### Human cell culture, viral infection, and proliferation assays

The human tumor cell lines HCT116 (CCL-247) and U-87 MG (HTB-14) were obtained from the ATCC. Cells were grown at 37 °C, 5% CO_2_ in a complete DMEM medium containing 4.5 g/L glucose and supplemented with 10% fetal bovine serum (FBS; DUTSCHER #S1810), L-glutamine, penicillin, and streptomycin. Dialyzed serum (same FBS as above) was obtained after dialysis in PBS (3 kDa-molecular weight cutoff) and was filtered at 0.1 μm. Lentiviruses expressing *PRPS1*, *PRPS2*, and mutants were obtained at the Vect’UB platform (TBMCore Bordeaux University, see Table S3). Titers were given in Transduction Units (TU) and were obtained by qPCR. Cells in suspension in culture medium were incubated with lentiviruses at 0, 1, 3, 5, 10, and 20 TU/cell and then inoculated at the cell density of 10,000 cells/cm^2^ in 6-well culture dishes. Cells were allowed to settle down at 37 °C for 24 h and 0.5 μg/ml puromycin was then added for 7 days with a medium change every 2 days. TU values retained for the following analyses were in the range of 5 to 20 depending on the cell type and transduction experiment. Transduction with the different constructs were performed in paralleled experiments, for the sake of better comparisons. Two independent productions of lentivirus were used and gave similar results.

For metabolites quantifications, cells were seeded in 6-well plates and grown up to half confluency. Cells were then washed and incubated for 24 h in the complete medium before metabolic extractions were performed as described in (34). Tri-, tetra- or penta-plicate conditions were used. For proliferation experiments, cells were seeded in 24-well plates at 7000 cells/cm^2^ and grown for 4 days at 37 °C with medium changes every 2 days. Triplicate or tetraplicate conditions were used. Cells were harvested by trypsinization and were counted using a Multisizer four Coulter counter (Beckman).

### Plasmids

All plasmids and oligonucleotides are listed in Tables S3 and S4, respectively. Plasmids allowing expression of human wild-type *PRPS1* and *PRPS2* in yeast were obtained by PCR amplification of the open reading frames (ORF) with the following pairs of oligonucleotides (5562 + 5563 for *PRPS1* and 5763 + 5764 for *PRPS2*) using, respectively, cDNA IRAUp969H0616D and IRCMp5012H0142D from Source Biosciences as templates. *PRPS1* Hyperactive mutants were obtained by site-directed mutagenesis. PCR products were cloned in the pCM189 or its derivative p2714 plasmids in which gene expression is under the control of tetracycline-repressible promoter. For expression of the same genes in mammalian cells, ORF were amplified with the 5678 + 5563 (*PRPS1*) and 5763 + 5764 (*PRPS2*) and cloned in the pLenti-MND-IRES-Puro^R^-WPRE. The *PrsA* expression plasmid was obtained by PCR amplification of *PrsA* ORF with oligonucleotides 2278 + 2279 on *E. coli* genomic DNA as a template and cloning in pCM189. The h*ENT1* encoding plasmid was a generous gift from Drs. Cass, Damaraju, and Sawyer. The plasmid expressing the hyperactive form of *ADE4* gene (*ADE4-1* mutant R328K) was obtained by cloning in pCM189 the PCR fragment amplified with oligonucleotides 48 + 429 on genomic DNA of the m131 *ADE4* mutant described in (31, 32). All plasmids used to express *PRS1*, *PRS2*, *PRS3*, *PRS4*, and *PRS5* (either wild-type or mutated alleles) genes were obtained by PCR amplification of ORFs using yeast genomic DNA as template (FY4 for wild-type genes and *PRS5* suppressors indicated in Table S1) and the following couples of oligonucleotides 2032 + 2033, 5847 + 5848, 5082 + 5083, 5849 + 5850 and 2040 + 2041 for *PRS1*, *PRS2*, *PRS3*, *PRS4*, and *PRS5*, respectively. PCR products were then clones in either centromeric (YCplac33) or multicopy (Yeplac181 or Yeplac195) plasmids as indicated in Table S3. For all plasmids, further construction details are available upon request.

### Yeast population doubling time

Cells were kept in exponential growing phase (<1.5 10^7^ cells/ml) for at least 24 h hours by successive dilutions before any measurement. Population doubling time was then determined by following cell number during 8 h on at least four independent exponential growth culture using the Multisizer four Coulter counter (Beckman). Population doubling time was then determined by exponential growth regression of the proliferation curves using GraphPad Prism 5.

### Biomass production rate

Cells were exponentially (<1.5 10^7^ cells/ml) grown in indicated medium for at least 24 h hours before measurement. Biomass was determined by filtration of aliquots of the cell culture (starting at comparable biomass for each strain around 10 mg corresponding to 50–100 ml at 1–2 × 10^6^ cells/ml) on 0.45 μm polyamid pre-weighted filters (25006–47-N; Sartorius) followed by two 10 ml washes with room temperature milliQ water and drying of the filter with successive 30 s cycles (until the mass of filter + cells is constant) in a microwave oven at 800 W. Dried biomass was measured in exponential growth conditions during 4 h and exponential biomass production rate was determined by linear regression of the experimental points (see Fig. S5 as an example).

### Metabolic analyses

Metabolic extractions of yeast or mammalian cells were performed using the EtOH boiling methodology as described in (34). Metabolite separation by ionic chromatography was either performed on an ICS3000 chromatography station (Dionex, Sunnyvale, CA) using a CarboPac PA1 column (250 × 2 mm; Dionex) as described in (35), or using the Integrion chromatography station (Thermo Electron) equipped with RFIC eluant generator and eluant suppressor (ADRS 600) and coupled to both a Vanquish diode array detector and a conductivity detector. Metabolites separation was achieved at 0.38 ml/min and 30 °C on an AS11-HC-4 μm (250 × 2 mm, Thermo Electron) with a Potassium hydroxide discontinuous gradient, provided by the eluant generator, starting at 1 mM for 7 min, rising up to 15 mM in 9 min, 30 mM in 9 min and 60 mM in 13 min, followed by a step at 60 mM for 7 min and finally increasing up to 80 mM in 8 min and stay at 80 mM for 29 min. The resin was then re-equilibrated at 1 mM KOH for 10 min before injection of a new sample. Metabolites were detected by conductimetry (PRPP) and by UV absorbance (nucleotides and derivatives) and identified by their UV spectrum signature and/or by injection with standards. For each yeast strain or human cell line, sample normalization was achieved based on cell number and median cell volume using a Multisizer 4 (Beckman Coulter). Peak area quantification was done on conductimetric recording for PRPP and with UV absorbance at 260 nm for all nucleotides and derivatives except for Cytidylic nucleotides and NADH which were quantified at 280 nm et 340 nm, respectively. The intracellular concentration of metabolites has been determined using standard curves obtained with pure compounds. The sum of each type of purine (Adenylic and Guanylic) and pyrimidine (Uridylic and Cytidylic) nucleotides is defined as [NXP] = [NMP] + [NDP] + [NTP]. All metabolic raw data are presented in Tables S5–S17.

### Statistical analyses

For yeast strains and/or human cell lines, growth and metabolites extraction were performed on three to eight biologically independent samples, as detailed in each figure legend. Statistics, given as *p*-values noted in each figure, were determined by Welch’s unpaired *t* test. Welch's *t* test is more robust than Student's *t* test and maintains type I error (rejection of the true null hypothesis) rates close to nominal for unequal variances.

## Data availability

All data are contained within the manuscript.

## Supporting information

This article contains supporting information (36, 37).

## Conflict of interest

The authors declare that they have no conflicts of interest with the content of this article.
