# Supplementary material for: On-demand utilization of phosphoribosyl pyrophosphate by downstream anabolic pathways
Source: J Biol Chem. 2023 Jul 4;299(8):105011. doi: 10.1016/j.jbc.2023.105011 (PMC10413152; doi:10.1016/j.jbc.2023.105011)
Supplement: Supporting information [file mmc1.pdf]

Supporting Information

On demand utilization of phosphoribosyl pyrophosphate by downstream anabolic pathways

Pinson, B., Moenner, M., Saint-Marc, C., Granger-Farbos, A and Daignan-Fornier B.

Supplementary Figures

Figure S1: Consequences of PRPP variations on mono-, di- and tri-phosphate nucleotide content. **p. 2**

Figure S2: Addition of all nucleotide precursors is required to suppress the growth defect of the *prs1 prs3* double mutant. **p. 3**

Figure S3: Modulation of PRPP utilization by the pyrimidine *de novo* pathway reveals that PRPP is limiting for nucleotide synthesis. **p. 4**

Figure S4: Expression of human *PRPS1* hyperactive variants in HCT116 human cells increases both PRPP and nucleotides content and affects cell proliferation. **p. 5**

Figure S5: Determination of biomass production rate in yeast. **p. 6**

Supplementary Tables

Table S1: List of the *prs1prs3* slow growth phenotype suppressors **p. 7**

Table S2: Yeast strains **p. 8**

Table S3: Plasmids **p. 9**

Table S4: Oligonucleotides **p. 10**

Table S5: Raw data for Figure 2: phenotypic and metabolic consequences of PRPP decrease in yeast. **p. 11**

Table S6: Raw data for Figure 3: Consequences of PRPP variations on nucleotide synthesis and precursor accumulation. **p. 12-13**

Table S7: Raw data for Figure 4: Metabolic and phenotypic consequences of *PRS1-PRS3* overexpression.**p.14**

Table S8: Raw data for Figure 5: PRPP content is limiting for yeast proliferation and nucleotide synthesis.**p.15**

Table S9: Raw data for Figure 6: Growth and metabolic defects of the *prs1 prs3* mutant are rescued by supplementation with PRPP-not-requiring precursors. **p. 16**

Table S10: Raw data for Figure 7: Suppression of *prs1 prs3* growth and metabolic defects by mutations in the *PRS5* gene. **p. 17**

Table S11: Raw data for Figure 8: PRPP and nucleotide content is increased by expression of human PRPS1 hyperactive variants. **p. 18-19**

Table S12: Raw data for Figure 9: Impairment of PRPP utilization by the purine de novo pathway reveals that PRPP is limiting for nucleotide synthesis. **p. 20**

Table S13: Raw data for Figure 10: Expression of human *PRPS1* hyperactive variants in human cells increases PRPP and nucleotide content and affects cell proliferation. **p. 21**

Table S14: Raw data for Figure 11: Addition of a purine precursor reveals that PRPP is limiting for synthesis of nucleotides in human cells. **p. 22**

Table S15: Raw data for Figure S1: Consequences of PRPP variations on mono-, di- and tri-phosphate nucleotide content. **p. 23-25**

Table S16: Raw data for Figure S3: Modulation of PRPP utilization by the pyrimidine *de novo* pathway reveals that PRPP is limiting for nucleotide synthesis. **p. 26**

Table S17: Raw data for Figure S4: Expression of human *PRPS1* hyperactive variants in HCT116 human cells increases PRPP and nucleotide content and affects cell proliferation. **p. 27**

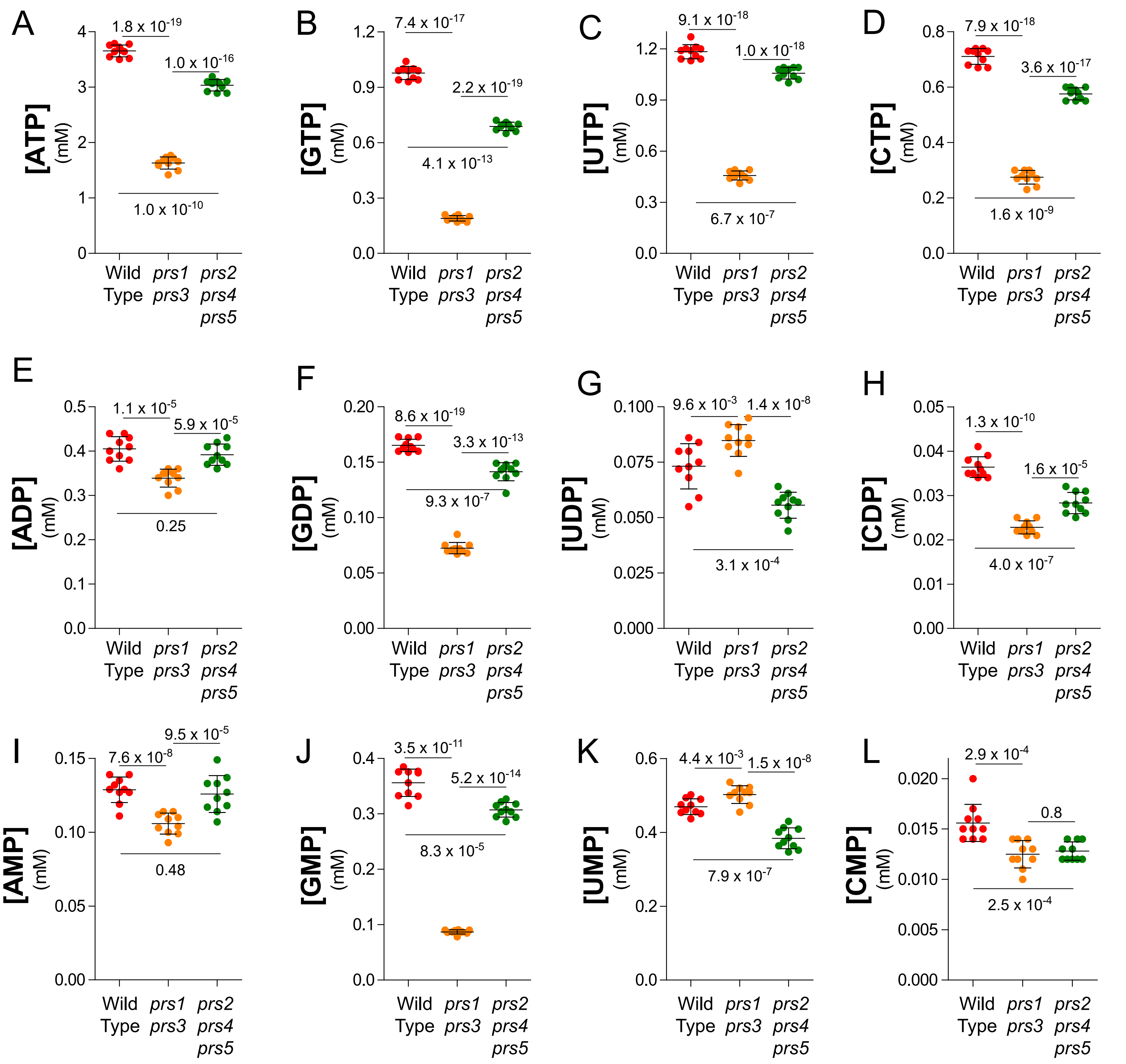

Figure S1. Consequences of PRPP variations on mono-, di- and tri-phosphate nucleotide content. Nucleotide content determination on metabolites extractions from Fig. 2 on the wild-type (Y12325), *prs1*Δ *prs3*Δ (Y12562) and *prs2*Δ *prs4*Δ *prs5*Δ (Y12649). These mono-, di- and triphosphate nucleotides content determinations were used to calculate the NXP content presented in Fig. 3. Each metabolite was quantified from 8 independent metabolite extractions. Numbers on each panels correspond to p-values calculated from a Welch's unpaired t-test.

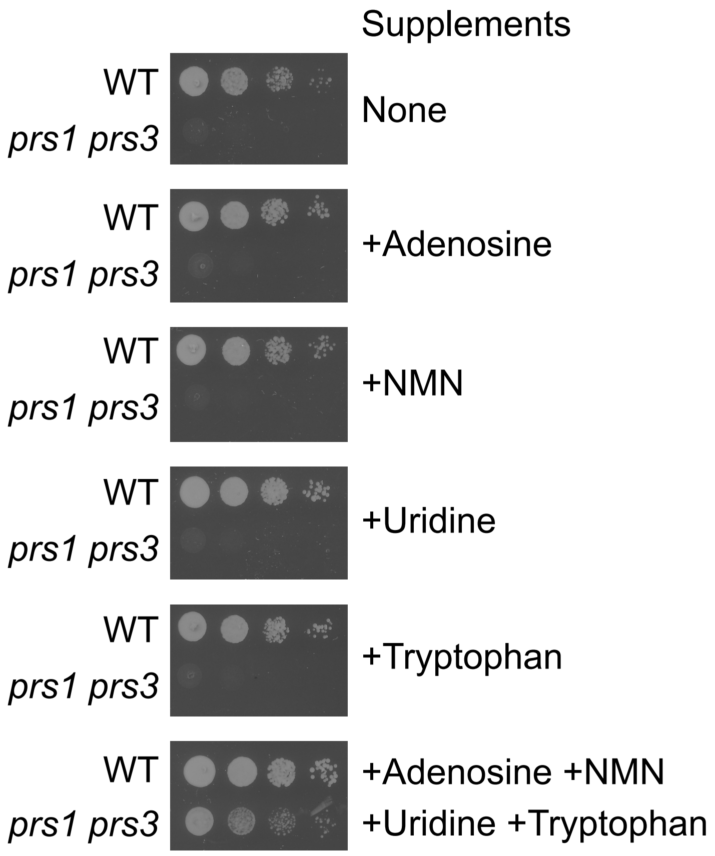

Figure S2. Addition of all nucleotide precursors is required to suppress the growth defect of *prs1 prs3* double mutant. Wild-type (Y11418) and *prs1*Δ *prs3*Δ (Y11985) strains were transformed with the h*ENT1* expressing plasmid (p4991). Transformants were selected on SDcasaW medium supplemented with adenosine (Ado, 300 µM), Uridine (Uri, 300 µM), Tryptophan (300 µM) and Nicotinamide mononucleotide (NMN, 100 µM). Transformants were serial diluted (1/10) and spotted on SDcasaW supplemented or not (None) with either Ado, Uri, Trp and NMN or the mix of all precursors. Plates were imaged after 36 h at 30°C.

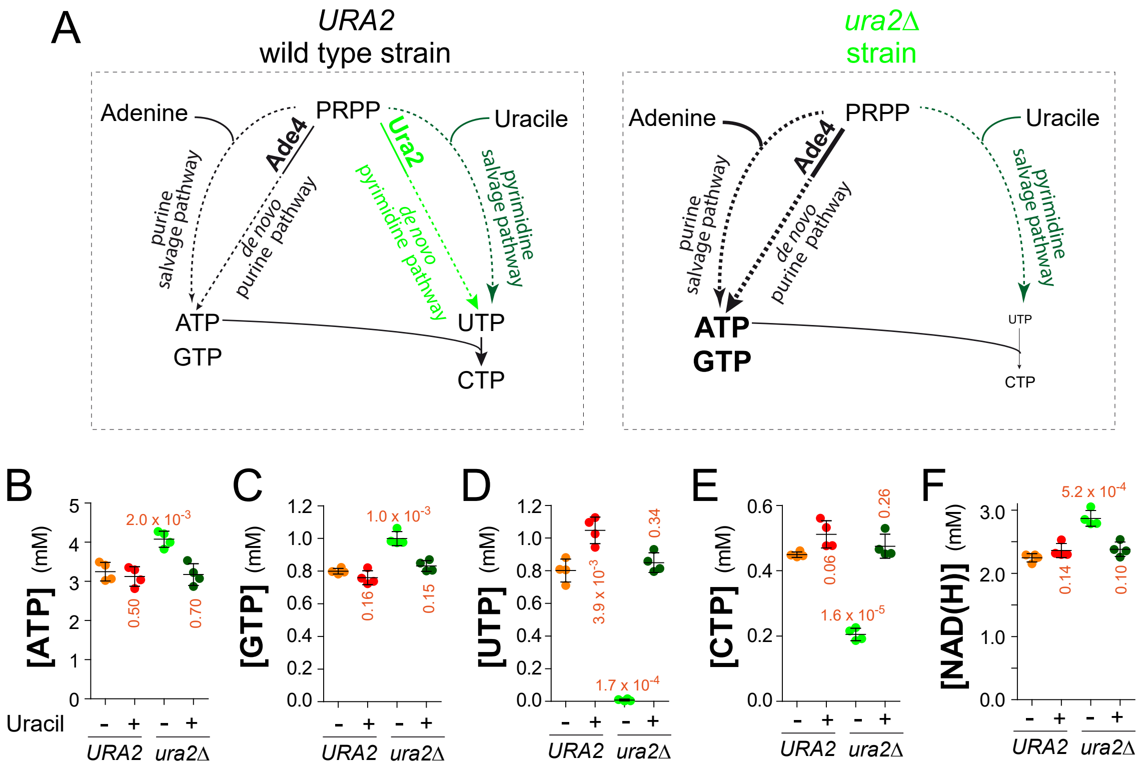

Figure S3. Modulation of PRPP utilization by the pyrimidine *de novo* pathway reveals that PRPP is limiting for nucleotide synthesis. *A*, Schematic representation of the *URA2* gene deletion on purine and pyrimidine nucleotides content. Ade4 and Ura2 correspond to the PRPP-dependent enzymes in the *de novo* purine and pyrimidine pathways, respectively. *B-F*, Determination of nucleotides content in yeast cells expressing or not *URA2* gene. Wild-type (FY4) and *ura2*Δ (Y12253) strains were exponentially grown for 24 h in SDcasaWAU liquid medium. Cells were then harvested by filtration and grown for 3 more hours in SDcasaWA liquid medium supplemented (+) or not (-) with uracil before metabolite extraction, separation and quantification. Metabolite measurements were performed on 4 independent extractions and red numbers correspond to the p-values calculated from a Welch's unpaired t-test.

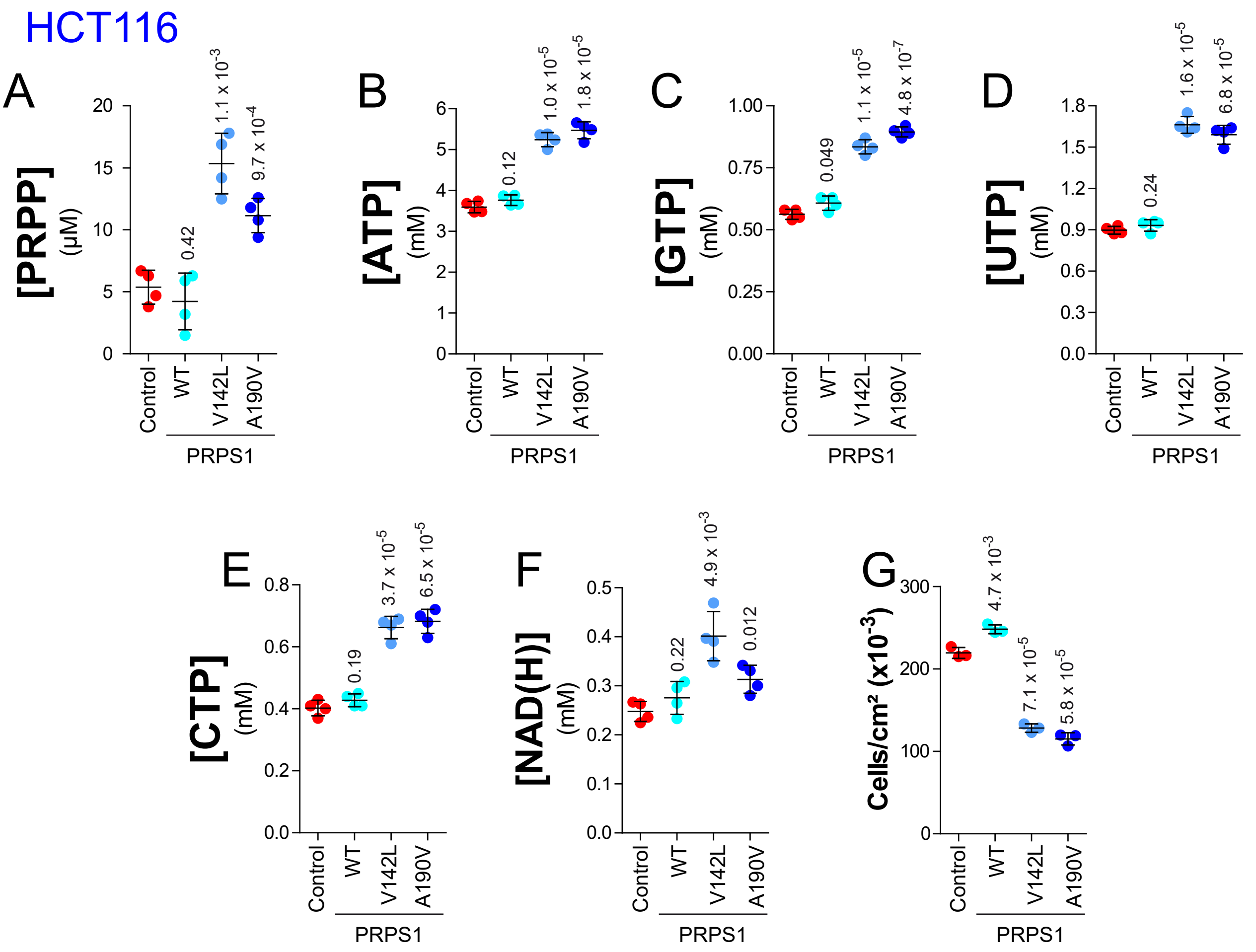

Figure S4. Expression of human *PRPS1* hyperactive variants in HCT116 human cells increases both PRPP and nucleotide content and affects cell proliferation. Lentiviral infection was used to express either wild-type human *PRPS1* (WT, p6033) or its hyperactive mutants (V142L, p6034 and A190V, p6035) in the human HCT116 cell line. Infection with the pLenti-MND-IRES-PuroR-WPRE (p5769) empty vector was used as control. Cells were grown in the complete medium. Metabolite content (A-F) was determined as described in "Experimental Procedures" and measurements were performed on 4 independent extractions. In cell proliferation experiments (G), cells were seeded at the density of 7,000 cells/cm^2 and grown for 4 days in complete medium before trypsinization and counting. Measurements were performed on 4 independent extractions and numbers correspond to the p-values calculated from a Welch's unpaired t-test.

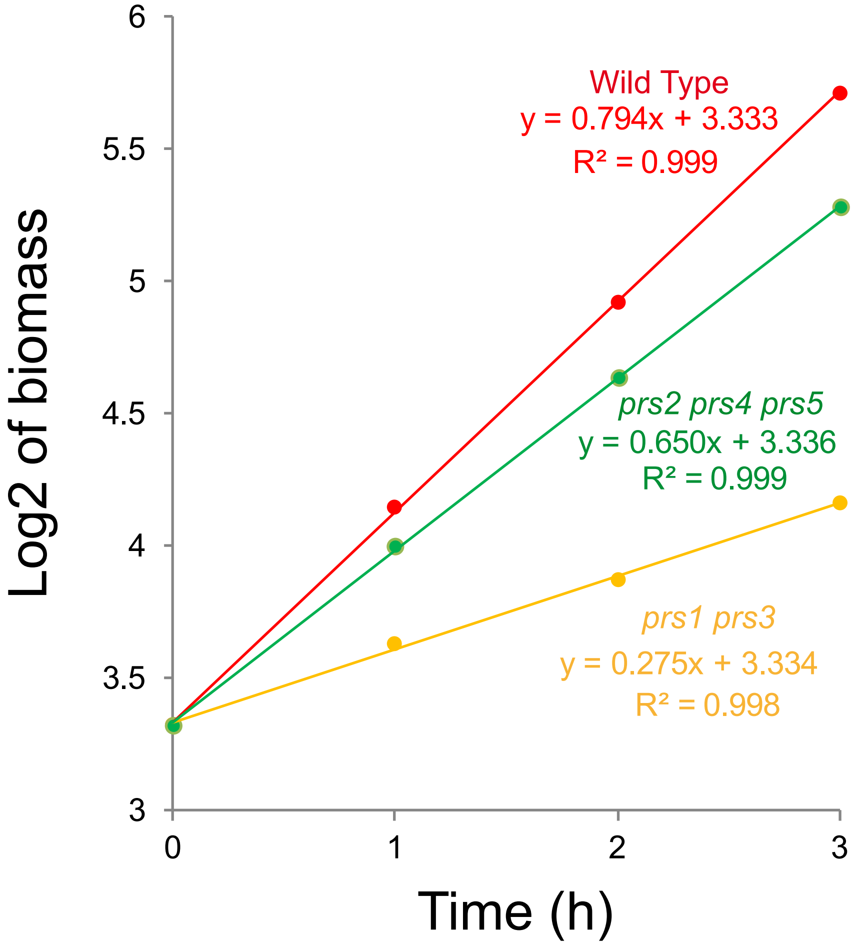

Figure S5. Determination of biomass production rate in yeast. Wild-type (Y12325), *prs1*Δ *prs3*Δ (12562) and *prs2*Δ *prs4*Δ *prs5*Δ (12649) were exponentially grown in SDcasaWAU medium for 24 h. Dry biomass was measured during 4 hours as described in "Experimental Procedures". This figure shows a representative measurement obtain for each strain. Exponential biomass production was determined by linear regression of the experimental points. Relative biomass production presented in Figure 2 corresponds ratio of the slopes obtained for four independent cultures for each strain.

Table S1: List of the *prs1prs3* suppressors either obtained on SD minimal medium or on SDcasaWAU rich medium

**Suppressor name**	**Nucleotide change in *PRS5***	**Amino acid change in Prs5**	**Selection Medium**
supp48	C299T	Pro100Leu	SD minimal medium
supp72	C298T	Pro100Ser	SDcasaWAU
supp44	G322A	Gly108Ser	SDcasaWAU
supp9	C328T	Pro110Ser	SDcasaWAU
supp3	C328T	Pro110Ser	SDcasaWAU
supp23	G655A-T656A	Val219Lys	SD minimal medium
supp16	A920T-T921C	His307Leu	SD minimal medium
supp36	A923C	Lys308Thr	SDcasaWAU
supp24	A922G	Lys308Glu	SD minimal medium
supp47	A924C	Lys317Asn	SD minimal medium
supp48	G932T	Arg311Ile	SDcasaWAU
supp56	G932T	Arg311Ile	SDcasaWAU
supp22	T934	Ser312Pro	SD minimal medium
supp46	T941C	Leu314Ser	SD minimal medium
supp22	A942T	Leu314Phe	SDcasaWAU
supp26	C953T	Pro318Leu	SD minimal medium
supp58	G1192A	Asp398Asn	SDcasaWAU
supp12	G1201A	Asp401Asn	SDcasaWAU
supp11	G1201A	Asp401Asn	SDcasaWAU
supp27	G1201A	Asp401Asn	SD minimal medium
supp8	T1207C	Ser403Pro	SDcasaWAU
supp6	T1207C	Ser403Pro	SDcasaWAU
supp24	T1207C	Ser403Pro	SDcasaWAU
supp27	C1214T	Thr405Ile	SDcasaWAU
supp61	T1291C	Ser431Pro	SDcasaWAU
supp34	T1291C	Ser431Pro	SD minimal medium
supp32	A1490G-G1491T	End497Cys	SD minimal medium
supp33	A1490G-G1491T	End497Cys	SD minimal medium
supp38	A1490G-G1491T	End497Cys	SD minimal medium
supp39	A1490G-G1491T	End497Cys	SD minimal medium

Table S2: Yeast strains

Strain Name	Genotype
BY4741	*MAT* **a** *ura3Δ0 leu2Δ0 his3Δ1 met15Δ0*
BY4742	*MAT* alpha *ura3Δ0 leu2Δ0 his3Δ1 lys2Δ0*
FY4	*MAT* **a**
FY5	*MAT* alpha
Y2850	*MAT* **a** *prs1::KanMX4 prs3::KanMX4 ura3Δ0 leu2ΔO his3Δ1 met15Δ0*
Y5217	*MAT* **a**/ *Mat* alpha *rki1::kanMX4/RKI1 ura3Δ0/ ura3Δ0 leu2Δ0/ leu2Δ0 his3Δ1/ his3Δ1 lys2Δ0/LYS2 met15Δ0/MET15*
Y9469	*pYPhENT1 fui1::TRP1 gal, ura3-52, trp1, lys2, ade2,hisΔ2000*
Y11418	*MAT* alpha *ura3ΔO*
Y11451	*MAT* alpha *ura3ΔO leu2Δ0*
Y11983	*MAT* **a** *ura3Δ0 prs1::KanMX4 prs3::KanMX4*
Y11985	*MAT* alpha *ura3Δ0 prs1::KanMX4 prs3::KanMX4*
Y12149	*MAT* alpha *prs1::KanMX4 prs3::KanMX4*
Y12204	*MAT* **a** *prs1::KanMX4 prs3::KanMX4 PRS5supp44 (G322A* : Gly108Ser)
Y12206	*MAT* **a** *prs1::KanMX4 prs3::KanMX4 PRS5supp58 (G1192A* : Asp398Asn)
Y12207	*MAT* **a** *prs1::KanMX4 prs3::KanMX4 PRS5supp61 (T1291C* : Ser431Pro)
Y12211	*MAT* alpha *prs1::KanMX4 prs3::KanMX4 PRS5supp29 (C1369T* : Pro457Ser)
Y12214	*MAT* **a** *prs1::KanMX4 prs3::KanMX4 PRS5supp3 (C328T* : Pro110Ser)
Y12253	*MAT* alpha *ura2 ::KanMX4 ura3ΔO*
Y12258	*MAT* alpha *ade4 ::KanMX4*
Y12269	*MAT* **a** *prs1::KanMX4 prs3::KanMX4 PRS5supp72 (C298T* : Pro100Ser)
Y12271	*MAT* alpha *prs1::KanMX4 prs3::KanMX4 PRS5supp48 (G932T* : Arg311Ile)
Y12272	*MAT* **a** *prs1::KanMX4 prs3::KanMX4 PRS5supp11 (G1201A* : Asp401Asn)
Y12325	*MAT* **a**
Y12469	*MAT* **a** *prs2::KanMX4 prs4::KanMX4 prs5::KanMX4*
Y12558	*MAT* **a** *prs1::KanMX4 prs3::KanMX4 PRS5supp23 (G665A T656A* : Val219Lys)
Y12560	*MAT* alpha *prs1::KanMX4 prs3::KanMX4 PRS5supp46 (T941C* : Leu314Ser)
Y12562	*MAT* **a** *prs1::KanMX4 prs3::KanMX4*
Y12563	*MAT* **a** *prs1::KanMX4 prs3::KanMX4*
Y12599	*MAT* **a** *ura3ΔO*

Table S3: Plasmids

Plasmid Name	Relevant description	Source
pCM189	*tetO$_7$-promoterURA3 CEN AmpR*	Gari *et al*
YCpLac33	*URA3 CEN AmpR*	Gietz and Sugino
YEpLac181	*LEU2 2µ AmpR*	Gietz and Sugino
YEPlac195	*URA3 2µ AmpR*	Gietz and Sugino
P2048	*tetO$_7$-promoter-ADE4-1(Arg328Lys) URA3 CEN AmpR*	This study
P2714	*tetO$_7$-promoter-HA URA3 CEN AmpR*	This study
P4991	*hENT1 URA3 2µ*	This study
P5671	*PRS3* in YEPlac195	This study
P5769	*pLenti-MND-IRES-PuroR-WPRE*	Vect'UB
P5909	*tetO$_7$-promoter-PrsA URA3 CEN AmpR*	This study
P5949	*tetO$_7$-promoter-HA-PRPS1 URA3 CEN AmpR*	This study
P5994	*tetO$_7$-promoter-HA-PRPS1* Val142Leu *URA3 CEN AmpR*	This study
P5995	*tetO$_7$-promoter-HA-PRPS1* Ala190Val *URA3 CEN AmpR*	This study
P6001	*tetO$_7$-promoter-PRPS1 URA3 CEN AmpR*	This study
P6008	*PRS5supp11* (D401N) in YEPlac195	This study
P6009	*PRS5supp11* (D401N) in YCpLac33	This study
P6011	*PRS5supp48* (R311I) in YEPlac195	This study
P6014	*PRS5supp48* (R311I) in YCpLac33	This study
P6015	*PRS5supp3* (P110S) in YEPlac195	This study
P6016	*PRS5supp3* (P110S) in YCpLac33	This study
P6017	*PRPS5* in YCpLac33	This study
P6019	*PRPS5* in YEPlac195	This study
P6020	*tetO$_7$-promoter-PRPS2 URA3 CEN AmpR*	This study
P6030	*PRPS2 in P5769*	This study
P6033	*PRPS1 in P5769*	This study
P6034	*PRPS1* Val142Leu *in P5769*	This study
P6035	*PRPS1* Ala190Val *in P5769*	This study
P6070	*PRS2* in YEpLac181	This study
P6094	*PRS4* in YEpLac181	This study
P6160	*PRS1* in YEpLac181	This study

E Garí, L Piedrafita, M Aldea and E Herrero A set of vectors with a tetracycline-regulatable promoter system for modulated gene expression in *Saccharomyces cerevisiae*. (1997) *Yeast* . **13**(9):837-48. doi: 10.1002/(SICI)1097-0061(199707)13:9<837::AID-YEA145>3.0.CO;2-T.

R D Gietz, A Sugino New yeast-*Escherichia coli* shuttle vectors constructed with *in vitro* mutagenized yeast genes lacking six-base pair restriction sites. (1988) *Gene* **74**(2):527-34. doi: 10.1016/0378-1119(88)90185-0

Vect'UB: Vectorology and virus production platform from TBMCore Bordeaux University, CNRS UAR 3427, INSERM US005

Table S4: Oligonucleotides

Oligonucleotide	5'- 3' Sequence
48	CGCGGATCCAAATGTGTGGTATTTTAG
429	AAACTGCAGTCGATAATGTGCACAATTATATAATC
2032	TCTTTGGAAGAAGTTGAAGG
2033	GATCGAATTCGGATGTTGTAATGTGTTTGG
2040	CGCGGATCCTTATATCCAGCATCTCATGC
2041	GATCGGTACCCCAACGGCTCCTCGTCGACC
2278	CTCAATGCGGCCGCTTAGTGTTCGAACATGGCAG
2279	CGCGGATCCAAGATGCCTGATATGAAGCTTTTTGC
5082	CGCGGATCCGGCCCAGAAATCTAAGCTTGAGG
5083	AAAACTGCAGGTTCCCGATGGCTCTATTTTGG
5562	CGCGGATCCAGGATGCCGAATATCAAAATCTTCAG
5563	CCAATGCATCTATTATAAAGGGACATGGCTGAATAGG
5678	CGCGGATCCATGCCGAATATCAAAATCTTCAGC
5763	GCCGGATCCACGCGTATGCCCAACATCGTGCTGTTC
5764	GGCCATATGATGCATTTATAGCGGGACATGGCTGAAC
5847	GCGGGATCCTTGCAATTTGCGAATGCGG
5848	AAAACTGCAGCATATTTTCCAAAAGCAAGGGCG
5849	GCGGGATCCGTACTGTAAAATGTGTCGTCGAC
5850	AAAACTGCAGGTAAAGGTTCACCGTCTCCT

Table S5: Raw data Figure 2

A: [PRPP]

	CD Peak area	CD Peak area	CD Peak area	µM in cells	µM in cells	µM in cells
**Metabolite**	**PRPP**	**PRPP**	**PRPP**	**PRPP**	**PRPP**	**PRPP**
**Strain**	**WT**	***prs1 prs3***	***prs2 prs4 prs5***	**WT**	***prs1 prs3***	***prs2 prs4 prs5***
sample 1	0,030	0,004	0,025	0,100	0,013	0,083
sample 2	0,031	0,003	0,024	0,103	0,010	0,080
sample 3	0,036	0,005	0,025	0,119	0,016	0,083
sample 4	0,034	0,005	0,021	0,115	0,015	0,071
sample 5	0,035	0,004	0,020	0,116	0,014	0,067
sample 6	0,033	0,006	0,022	0,111	0,019	0,073
sample 7	0,038	0,004	0,021	0,128	0,014	0,071
sample 8	0,034	0,004	0,024	0,113	0,014	0,082
sample 9	0,034	0,004	0,020	0,112	0,013	0,066
sample 10	0,034	0,004	0,022	0,113	0,014	0,072
**Mean**	0,034	0,004	0,022	0,113	0,014	0,075
**SD**	0,002	0,001	0,002	0,008	0,002	0,007
Unpaired t-test vsWT		8,1E-13	8,4E-10		8,1E-13	8,4E-10
Unpaired t-test vs *prs1prs3*	8,1E-13		9,1E-12	8,1E-13		9,1E-12

B: Adenylic nucleotides content: [AXP] = [ATP] +[ADP] + [AMP]

	mM in cells	mM in cells	mM in cells
**Metabolite**	**AXP**	**AXP**	**AXP**
**Strain**	**WT**	***prs1 prs3***	***prs2 prs4 prs5***
sample 1	4,08	1,90	3,40
sample 2	4,01	1,82	3,40
sample 3	4,02	2,06	3,70
sample 4	4,14	2,12	3,38
sample 5	4,26	2,18	3,65
sample 6	4,21	2,06	3,62
sample 7	4,33	2,20	3,57
sample 8	4,36	2,09	3,59
sample 9	4,34	2,22	3,66
sample 10	4,19	2,14	3,55
**Mean**	4,19	2,08	3,55
**SD**	0,13	0,13	0,12
Unpaired t-test vsWT		2,2E-18	1,1E-09
Unpaired t-test vs *prs1prs3*	2,2E-18		7,2E-16

C: Relative biomass production rate

	Relative Biomass Production	Relative Biomass Production	Relative Biomass Production
	**WT**	***prs1 prs3***	***prs2 prs4 prs5***
sample 1	1,023	0,609	0,848
sample 2	1,026	0,641	0,816
sample 3	0,960	0,624	0,800
sample 4	0,990	0,634	0,827
sample 5			0,831
Unpaired t-test vsWT		2,1E-05	2,9E-04
Unpaired t-test vs *prs1prs3*	2,1E-05		3,2E-07

D: Median volume

	Median volume (fl)	Median volume (fl)	Median volume (fl)
	**WT**	***prs1 prs3***	***prs2 prs4 prs5***
sample 1	53,8	40	51,5
sample 2	53,4	41	51,3
sample 3	54,1	40,5	52,1
sample 4	55,8	41,1	52
sample 5	53,3	38,8	50,4
sample 6	53,9	39,7	50
sample 7	55,3	38,9	50
sample 8	54,2	39,02	51,6
sample 9	55	36,9	50,6
sample 10	56	38,1	50,6
**Mean**	54,5	39,4	51,0
**SD**	1,0	1,3	0,8
Unpaired t-test vsWT		1,3E-15	9,5E-08
Unpaired t-test vs *prs1prs3*	1,3E-15		4,0E-13

E: Population doubling Time

	Doubling Time (min)	Doubling Time (min)	Doubling Time (min)
	**WT**	***prs1 prs3***	***prs2 prs4 prs5***
sample 1	78,5	235,2	85,1
sample 2	79,6	215,7	83,3
sample 3	84,4	210,6	76,3
sample 4	77,0	218,8	79,4
Unpaired t-test vsWT		4,1E-05	0,67
Unpaired t-test vs *prs1prs3*	4,1E-05		2,4E-05

Table S6: Raw data Figure 3 (part 1/2)

A: [GXP] in mM — [GXP]= [GTP] + [GDP] + [GMP]

	µM in extract	µM in extract	µM in extract	mM in cells	mM in cells	mM in cells
**Metabolite**	**GXP**	**GXP**	**GXP**	**GXP**	**GXP**	**GXP**
**Strain**	**WT**	***prs1 prs3***	***prs2 prs4 prs5***	**WT**	***prs1 prs3***	***prs2 prs4 prs5***
sample 1	49,47	10,89	37,27	1,48	0,33	1,12
sample 2	49,51	10,73	36,93	1,49	0,32	1,11
sample 3	47,31	11,17	39,49	1,42	0,34	1,18
sample 4	47,93	11,82	35,70	1,44	0,35	1,07
sample 5	51,17	11,36	39,17	1,54	0,34	1,18
sample 6	50,75	11,30	38,34	1,52	0,34	1,15
sample 7	51,10	12,81	37,60	1,53	0,38	1,13
sample 8	49,83	12,41	37,93	1,50	0,37	1,14
sample 9	51,45	12,09	38,92	1,54	0,36	1,17
sample 10	50,13	11,97	37,97	1,50	0,36	1,14
**Mean**	49,865	11,657	37,931	1,50	0,35	1,14
**SD**	1,380	0,677	1,137	0,04	0,02	0,03
Unpaired t-test vs WT		6,6E-19	8,0E-14		6,6E-19	8,0E-14
Unpaired t-test vs *prs1 prs3*	6,6E-19		3,0E-19	6,6E-19		3,0E-19

B: [UXP] in mM — [UXP]= [UTP] + [UDP] + [UMP]

	µM in extract	µM in extract	µM in extract	mM in cells	mM in cells	mM in cells
**Metabolite**	**UXP**	**UXP**	**UXP**	**UXP**	**UXP**	**UXP**
**Strain**	**WT**	***prs1 prs3***	***prs2 prs4 prs5***	**WT**	***prs1 prs3***	***prs2 prs4 prs5***
sample 1	56,040	32,973	48,196	1,681	0,989	1,446
sample 2	55,197	32,181	49,817	1,656	0,965	1,495
sample 3	54,798	33,728	52,625	1,644	1,012	1,579
sample 4	57,683	35,312	47,454	1,730	1,059	1,424
sample 5	58,523	35,217	51,373	1,756	1,057	1,541
sample 6	58,443	34,272	49,669	1,753	1,028	1,490
sample 7	60,400	36,739	50,148	1,812	1,102	1,504
sample 8	56,683	35,184	50,153	1,700	1,056	1,505
sample 9	59,199	37,132	50,857	1,776	1,114	1,526
sample 10	58,292	35,478	48,329	1,749	1,064	1,450
**Mean**	57,526	34,822	49,862	1,73	1,04	1,50
**SD**	1,804	1,559	1,565	0,05	0,05	0,05
Unpaired t-test vs WT		1,3E-16	8,6E-09		1,3E-16	8,6E-09
Unpaired t-test vs *prs1 prs3*	1,3E-16		2,7E-14	1,3E-16		2,7E-14

C: [CXP] in mM — [CXP]= [CTP] + [CDP] + [CMP]

	µM in extract	µM in extract	µM in extract	mM in cells	mM in cells	mM in cells
**Metabolite**	**CXP**	**CXP**	**CXP**	**CXP**	**CXP**	**CXP**
**Strain**	**WT**	***prs1 prs3***	***prs2 prs4 prs5***	**WT**	***prs1 prs3***	***prs2 prs4 prs5***
sample 1	24,212	9,268	19,723	0,726	0,278	0,592
sample 2	24,354	9,000	19,873	0,731	0,270	0,596
sample 3	23,919	9,949	21,362	0,718	0,298	0,641
sample 4	26,286	11,194	21,187	0,789	0,336	0,636
sample 5	26,182	10,262	21,354	0,785	0,308	0,641
sample 6	25,569	10,136	20,033	0,767	0,304	0,601
sample 7	26,416	11,044	20,666	0,792	0,331	0,620
sample 8	25,329	10,340	20,680	0,760	0,310	0,620
sample 9	26,152	11,146	21,103	0,785	0,334	0,633
sample 10	26,135	10,696	19,788	0,784	0,321	0,594
**Mean**	25,455	10,304	20,577	0,76	0,31	0,62
**SD**	0,957	0,755	0,669	0,03	0,02	0,02
Unpaired t-test vs WT		3,4E-18	4,6E-10		3,4E-18	4,6E-10
Unpaired t-test vs *prs1 prs3*	3,4E-18		3,4E-17	3,4E-18		3,4E-17

D: [NAD(H)] in mM — [NAD(H)]= [NAD$^+$] + [NADH]

	µM in extracts	µM in extracts	µM in extracts	mM in cells	mM in cells	mM in cells
**Metabolite**	**NAD(H)**	**NAD(H)**	**NAD(H)**	**NAD(H)**	**NAD(H)**	**NAD(H)**
**Strain**	**WT**	***prs1 prs3***	***prs2 prs4 prs5***	**WT**	***prs1 prs3***	***prs2 prs4 prs5***
sample 1	65,569	25,579	58,267	1,967	0,767	1,748
sample 2	64,564	23,622	59,418	1,937	0,709	1,783
sample 3	63,677	26,726	67,591	1,910	0,802	2,028
sample 4	68,003	30,409	60,689	2,040	0,912	1,821
sample 5	72,037	32,555	61,811	2,161	0,977	1,854
sample 6	65,645	30,129	63,264	1,969	0,904	1,898
sample 7	70,663	34,888	62,450	2,120	1,047	1,873
sample 8	67,335	32,168	60,773	2,020	0,965	1,823
sample 9	65,470	36,792	63,601	1,964	1,104	1,908
sample 10	68,388	36,886	60,739	2,052	1,107	1,822
**Mean**	67,135	30,975	61,861	2,01	0,93	1,86
**SD**	2,684	4,598	2,601	0,08	0,14	0,08
Unpaired t-test vs WT		2,1E-12	3,0E-04		2,1E-12	3,0E-04
Unpaired t-test vs *prs1 prs3*	2,1E-12		2,4E-11	2,1E-12		2,4E-11

E: [Uracil] in mM

	µM in extracts	µM in extracts	µM in extracts	mM in cells	mM in cells	mM in cells
**Metabolite**	**Uracil**	**Uracil**	**Uracil**	**Uracil**	**Uracil**	**Uracil**
**Strain**	**WT**	***prs1 prs3***	***prs2 prs4 prs5***	**WT**	***prs1 prs3***	***prs2 prs4 prs5***
sample 1	8,148	25,407	15,385	0,244	0,762	0,462
sample 2	8,148	22,481	14,944	0,244	0,674	0,448
sample 3	8,185	24,093	17,470	0,246	0,723	0,524
sample 4	8,578	26,156	15,495	0,257	0,785	0,465
sample 5	8,900	25,828	17,319	0,267	0,775	0,520
sample 6	10,226	24,568	16,080	0,307	0,737	0,482
sample 7	7,037	25,132	15,565	0,211	0,754	0,467
sample 8	8,137	24,253	14,857	0,244	0,728	0,446
sample 9	7,893	29,397	16,402	0,237	0,882	0,492
sample 10	8,574	24,532	14,260	0,257	0,736	0,428
**Mean**	8,383	25,185	15,778	0,25	0,76	0,47
**SD**	0,817	1,805	1,046	0,02	0,05	0,03
Unpaired t-test vs WT		1,9E-12	2,3E-12		1,9E-12	2,3E-12
Unpaired t-test vs *prs1 prs3*	1,9E-12		6,7E-10	1,9E-12		6,7E-10

Table S6: Raw data Figure 3 (part 2/2)

F: [Adenine]in mM

	µM in extracts	µM in extracts	µM in extracts	mM in cells	mM in cells	mM in cells
**Metabolite**	**Adenine**	**Adenine**	**Adenine**	**Adenine**	**Adenine**	**Adenine**
**Strain**	**WT**	***prs1 prs3***	***prs2 prs4 prs5***	**WT**	***prs1 prs3***	***prs2 prs4 prs5***
sample 1	1,172	5,325	1,169	0,035	0,160	0,035
sample 2	2,272	3,059	1,384	0,068	0,092	0,042
sample 3	1,602	2,185	1,189	0,048	0,066	0,036
sample 4	1,123	4,156	1,267	0,034	0,125	0,038
sample 5	1,405	3,635	1,026	0,042	0,109	0,031
sample 6	2,092	2,999	0,844	0,063	0,090	0,025
sample 7	1,534	3,920	1,622	0,046	0,118	0,049
sample 8	2,334	3,391	2,461	0,070	0,102	0,074
sample 9	2,131	5,116	2,343	0,064	0,153	0,070
sample 10	2,501	4,636	1,450	0,075	0,139	0,043
**Mean**	1,817	3,842	1,476	0,05	0,12	0,04
**SD**	0,507	0,993	0,535	0,02	0,03	0,02
Unpaired t-test vs WT		6,0E-05	1,6E-01		6,0E-05	0,16
Unpaired t-test vs *prs1 prs3*	6,0E-05		1,2E-05	6,0E-05		1,2E-05

G: [Hypoxanthine] in mM

	µM in extracts	µM in extracts	µM in extracts	mM in cells	mM in cells	mM in cells
**Metabolite**	**Hypoxanthine**	**Hypoxanthine**	**Hypoxanthine**	**Hypoxanthine**	**Hypoxanthine**	**Hypoxanthine**
**Strain**	**WT**	***prs1 prs3***	***prs2 prs4 prs5***	**WT**	***prs1 prs3***	***prs2 prs4 prs5***
sample 1	18,085	41,274	15,389	0,543	1,238	0,462
sample 2	17,604	41,718	15,674	0,528	1,252	0,470
sample 3	17,005	40,155	13,765	0,510	1,205	0,413
sample 4	17,273	40,680	16,997	0,518	1,220	0,510
sample 5	17,411	42,033	17,643	0,522	1,261	0,529
sample 6	17,315	40,724	14,890	0,519	1,222	0,447
sample 7	16,266	43,120	14,311	0,488	1,294	0,429
sample 8	16,103	40,569	14,498	0,483	1,217	0,435
sample 9	17,841	39,830	16,133	0,535	1,195	0,484
sample 10	17,137	40,368	15,575	0,514	1,211	0,467
**Mean**	17,204	41,047	15,487	0,52	1,23	0,46
**SD**	0,627	0,999	1,206	0,02	0,03	0,04
Unpaired t-test vs WT		7,7E-20	1,4E-03		7,7E-20	1,4E-03
Unpaired t-test vs *prs1 prs3*	7,7E-20		1,8E-20	7,7E-20		1,8E-20

Table S7: Raw data Figure 4

A: [PRPP] in mM

			mM	mM
	Peak area	Peak area	in cells	in cells
Metabolite	PRPP	PRPP	PRPP	PRPP
Strain	WT/vectors	WT/*PRS1* + *PRS3*	WT/vectors	WT/*PRS1* + *PRS3*
sample 1	0,014	0,024	0,074	0,129
sample 2	0,015	0,029	0,079	0,154
sample 3	0,017	0,028	0,088	0,147
sample 4	0,015	0,025	0,079	0,134
sample 5	0,014	0,025	0,077	0,132
sample 6	0,012	0,026	0,065	0,137
sample 7	0,016	0,025	0,085	0,133
sample 8	0,015	0,024	0,079	0,127
Mean	0,015	0,026	0,007	0,137
SD	0,001	0,002	0,008	0,009
Unpaired t-test	2,4E-09		2,4E-09	

B: [ATP] in mM

			mM	mM
	Peak area	Peak area	in cells	in cells
Metabolite	ATP	ATP	ATP	ATP
Strain	WT/vectors	WT/*PRS1* + *PRS3*	WT/vectors	WT/*PRS1* + *PRS3*
sample 1	17,96	19,84	3,52	3,89
sample 2	16,45	21,93	3,23	4,30
sample 3	17,44	21,29	3,42	4,17
sample 4	17,43	21,38	3,42	4,19
sample 5	17,68	20,64	3,47	4,05
sample 6	16,97	21,08	3,33	4,13
sample 7	17,21	20,78	3,38	4,08
sample 8	18,13	21,68	3,55	4,25
Mean	17,41	21,08	3,41	4,13
SD	0,54	0,66	0,11	0,13
Unpaired t-test	1,1E-08		1,1E-08	

C: [GTP] in mM

			mM	mM
	Peak area	Peak area	in cells	in cells
Metabolite	GTP	GTP	GTP	GTP
Strain	WT/vectors	WT/*PRS1* + *PRS3*	WT/vectors	WT/*PRS1* + *PRS3*
sample 1	3,09	3,51	0,82	0,94
sample 2	2,70	3,91	0,72	1,04
sample 3	3,00	3,86	0,80	1,03
sample 4	2,95	3,93	0,78	1,05
sample 5	3,02	3,81	0,80	1,02
sample 6	2,95	3,81	0,79	1,01
sample 7	2,92	3,77	0,78	1,00
sample 8	3,13	3,94	0,83	1,05
Mean	2,97	3,82	0,79	1,02
SD	0,13	0,14	0,04	0,04
Unpaired t-test	5,7E-09		5,7E-09	

D: [UTP] in mM

			mM	mM
	Peak area	Peak area	in cells	in cells
Metabolite	UTP	UTP	UTP	UTP
Strain	WT/vectors	WT/*PRS1* + *PRS3*	WT/vectors	WT/*PRS1* + *PRS3*
sample 1	2,11	2,42	0,85	0,98
sample 2	1,78	2,58	0,72	1,04
sample 3	1,93	2,52	0,78	1,02
sample 4	1,81	2,63	0,73	1,06
sample 5	2,11	2,51	0,85	1,01
sample 6	1,82	2,74	0,74	1,11
sample 7	1,85	2,48	0,75	1,00
sample 8	1,96	2,55	0,79	1,03
Mean	1,92	2,55	0,78	1,03
SD	0,13	0,10	0,05	0,04
Unpaired t-test	6,8E-08		6,8E-08	

E: [CTP] in mM

			mM	mM
	Peak area	Peak area	in cells	in cells
Metabolite	CTP	CTP	CTP	CTP
Strain	WT/vectors	WT/*PRS1* + *PRS3*	WT/vectors	WT/*PRS1* + *PRS3*
sample 1	2,08	2,31	0,93	1,03
sample 2	1,92	2,58	0,86	1,16
sample 3	1,97	2,49	0,88	1,12
sample 4	2,03	2,49	0,91	1,12
sample 5	2,08	2,42	0,93	1,08
sample 6	1,98	2,45	0,89	1,10
sample 7	1,99	2,45	0,89	1,10
sample 8	2,08	2,55	0,93	1,14
Mean	2,02	2,47	0,91	1,11
SD	0,06	0,09	0,03	0,04
Unpaired t-test	2,4E-08		2,4E-08	

F: [NAD(H)] in mM

			mM	mM
	µM in sample	µM in sample	in cells	in cells
Metabolite	NAD(H)	NAD(H)	NAD(H)	NAD(H)
Strain	WT/vectors	WT/*PRS1* + *PRS3*	WT/vectors	WT/*PRS1* + *PRS3*
sample 1	45,31	51,44	1,81	2,06
sample 2	45,80	57,23	1,83	2,29
sample 3	47,71	56,28	1,91	2,25
sample 4	49,40	57,54	1,98	2,30
sample 5	49,89	55,49	2,00	2,22
sample 6	50,85	59,97	2,03	2,40
sample 7	50,31	58,10	2,01	2,32
sample 8	51,96	61,24	2,08	2,45
Mean	48,90	57,16	1,96	2,29
SD	2,40	2,97	0,10	0,12
Unpaired t-test	3,2E-05		3,2E-05	

G: [IMP] in mM

			mM	mM
	Peak area	Peak area	in cells	in cells
Metabolite	IMP	IMP	IMP	IMP
Strain	WT/vectors	WT/*PRS1* + *PRS3*	WT/vectors	WT/*PRS1* + *PRS3*
sample 1	0,09	0,29	0,05	0,17
sample 2	0,06	0,34	0,04	0,20
sample 3	0,05	0,26	0,03	0,15
sample 4	0,05	0,26	0,03	0,15
sample 5	0,06	0,26	0,03	0,15
sample 6	0,04	0,30	0,03	0,18
sample 7	0,04	0,27	0,02	0,16
sample 8	0,04	0,29	0,02	0,17
Mean	0,05	0,28	0,03	0,17
SD	0,02	0,03	0,01	0,02
Unpaired t-test	7,1E-10		7,1E-10	

H: [Inosine] in mM

			mM	mM
	Peak area	Peak area	in cells	in cells
Metabolite	Inosine	Inosine	Inosine	Inosine
Strain	WT/vectors	WT/*PRS1* + *PRS3*	WT/vectors	WT/*PRS1* + *PRS3*
sample 1	0,79	5,83	0,20	1,51
sample 2	0,83	7,04	0,21	1,82
sample 3	0,83	5,94	0,21	1,54
sample 4	0,93	6,16	0,24	1,60
sample 5	0,75	5,83	0,19	1,51
sample 6	0,75	6,83	0,19	1,77
sample 7	0,82	5,99	0,21	1,55
sample 8	1,04	6,34	0,27	1,64
Mean	0,84	6,25	0,22	1,62
SD	0,10	0,46	0,03	0,12
Unpaired t-test	1,9E-09		1,9E-09	

I: Population doubling Time (min)

	Doubling Time (min)	Doubling Time (min)
	WT/vectors	WT/*PRS1* + *PRS3*
sample 1	106,3	120,5
sample 2	107,1	117,0
sample 3	111,7	114,3
sample 4	106,8	116,5
Mean	108,0	117,1
SD	2,5	2,6
Unpaired t-test	2,3E-03	

J: Relative biomass production

	Relative biomass production rate	Relative biomass production rate
	WT/vectors	WT/*PRS1* + *PRS3*
sample 1	0,998	0,922
sample 2	1,017	0,941
sample 3	0,998	0,941
sample 4	0,988	0,941
Mean	1,000	0,936
SD	0,012	0,010
Unpaired t-test	2,0E-04	

K: Median volume (fl)

	Median Volume (fl)	Median Volume (fl)
	WT/vectors	WT/*PRS1* + *PRS3*
sample 1	48,2	52,1
sample 2	49,5	50,1
sample 3	50,4	53,2
sample 4	48,2	50,9
sample 5	51,4	49,6
sample 6	51,1	50,5
sample 7	50,3	52,6
sample 8	48,5	49,1
Mean	49,7	51,0
SD	1,3	1,5
Unpaired t-test	7,9E-02	

Table S8: Raw data Figure 5

B: Population doubling Time (min)

	Doubling Time (min)	Doubling Time (min)	Doubling Time (min)	Doubling Time (min)	Doubling Time (min)
	WT/vector	*prs1 prs3/vector*	*prs1 prs3/PRPS1*	*prs1 prs3/PRPS2*	*prs1 prs3/PrsA*
Strain 1	100,5	405,1	95,4	101,3	99,6
Strain 2	96,1	335,6	103,8	106,6	121,4
Strain 3	92,3	357,5	101,2	101,6	107,9
Strain 4	96,3	355,3	99,6	103,8	110,1
Strain 5	93,7	398,7			
Unpaired t-test *vs prs1 prs3*	2,9E-05		2,9E-05	3,4E-05	1,1E-05

C: [PRPP] in mM

						mM	mM	mM	mM	mM
	Peak area	Peak area	Peak area	Peak area	Peak area	in cells	in cells	in cells	in cells	in cells
**Metabolite**	PRPP	PRPP	PRPP	PRPP	PRPP	PRPP	PRPP	PRPP	PRPP	PRPP
**Strain**	WT/vector	*prs1 prs3/vector*	*prs1 prs3/PRPS1*	*prs1 prs3/PRPS2*	*prs1 prs3/PRSA*	WT/vector	*prs1 prs3/vector*	*prs1 prs3/PRPS1*	*prs1 prs3/PRPS2*	*prs1 prs3/PrsA*
sample 1	0,034	0,004	0,017	0,016	0,012	0,14	0,02	0,07	0,06	0,05
sample 2	0,031	0,006	0,015	0,015	0,013	0,12	0,02	0,06	0,06	0,05
sample 3	0,033	0,007	0,016	0,017	0,013	0,13	0,03	0,06	0,07	0,05
sample 4	0,033	0,006	0,019	0,013	0,011	0,13	0,02	0,08	0,05	0,04
sample 5	0,036	0,010	0,017	0,015	0,015	0,14	0,04	0,07	0,06	0,06
sample 6	0,032	0,006	0,016	0,015	0,015	0,13	0,02	0,06	0,06	0,06
**Mean**	0,03	0,01	0,02	0,02	0,01	0,13	0,03	0,07	0,06	0,05
**SD**	0,00	0,00	0,00	0,00	0,00	0,01	0,01	0,01	0,01	0,01
Unpaired t-test *vs prs1 prs3*	3,3E-10		2,9E-06	1,1E-05	9,2E-05	3,3E-10		2,9E-06	1,1E-05	9,2E-05

D: [ATP] in mM

						mM	mM	mM	mM	mM
	Peak area	Peak area	Peak area	Peak area	Peak area	in cells	in cells	in cells	in cells	in cells
**Metabolite**	ATP	ATP	ATP	ATP	ATP	ATP	ATP	ATP	ATP	ATP
**Strain**	WT/vector	*prs1 prs3/vector*	*prs1 prs3/PRPS1*	*prs1 prs3/PRPS2*	*prs1 prs3/PRSA*	WT/vector	*prs1 prs3/vector*	*prs1 prs3/PRPS1*	*prs1 prs3/PRPS2*	*prs1 prs3/PRSA*
sample 1	22,82	11,32	14,98	16,46	14,61	3,36	1,66	2,20	2,42	2,15
sample 2	21,79	10,26	13,41	14,53	14,19	3,20	1,51	1,97	2,14	2,09
sample 3	22,07	11,85	15,09	15,30	14,20	3,25	1,74	2,22	2,25	2,09
sample 4	22,25	10,30	13,27	15,00	14,63	3,27	1,51	1,95	2,21	2,15
sample 5	23,05	10,89	14,02	15,64	14,99	3,39	1,60	2,06	2,30	2,20
sample 6	23,98	9,89	13,75	15,42	15,34	3,53	1,45	2,02	2,27	2,26
**Mean**	22,66	10,75	14,09	15,39	14,66	3,33	1,58	2,07	2,26	2,16
**SD**	0,80	0,74	0,78	0,65	0,45	0,12	0,11	0,11	0,10	0,07
Unpaired t-test *vs prs1 prs3*	1,3E-10		1,9E-05	4,9E-07	3,1E-06	1,3E-10		1,9E-05	4,9E-07	3,1E-06

E: [GTP] in mM

						mM	mM	mM	mM	mM
	Peak area	Peak area	Peak area	Peak area	Peak area	in cells	in cells	in cells	in cells	in cells
**Metabolite**	GTP	GTP	GTP	GTP	GTP	GTP	GTP	GTP	GTP	GTP
**Strain**	WT/vector	*prs1 prs3/vector*	*prs1 prs3/PRPS1*	*prs1 prs3/PRPS2*	*prs1 prs3/PRSA*	WT	*prs1 prs3*	*prs1 prs3/PRPS1*	*prs1 prs3/PRPS2*	*prs1 prs3/PrsA*
sample 1	4,74	0,89	2,04	2,25	1,67	0,95	0,18	0,41	0,45	0,33
sample 2	4,44	0,81	1,81	1,82	1,68	0,89	0,16	0,36	0,36	0,34
sample 3	4,40	0,93	2,02	2,10	1,63	0,88	0,19	0,40	0,42	0,33
sample 4	4,35	0,80	1,98	1,81	1,69	0,87	0,16	0,40	0,36	0,34
sample 5	4,63	0,86	1,78	2,02	1,83	0,92	0,17	0,36	0,40	0,37
sample 6	4,58	0,82	1,79	1,95	1,89	0,91	0,16	0,36	0,39	0,38
**Mean**	4,52	0,85	1,90	1,99	1,73	0,90	0,17	0,38	0,40	0,35
**SD**	0,15	0,05	0,12	0,17	0,10	0,03	0,01	0,02	0,03	0,02
Unpaired t-test *vs prs1 prs3*	1,6E-09		4,0E-07	4,9E-06	2,1E-07	1,6E-09		4,0E-07	4,9E-06	2,1E-07

E: [UTP] in mM

						mM	mM	mM	mM	mM
	Peak area	Peak area	Peak area	Peak area	Peak area	in cells	in cells	in cells	in cells	in cells
**Metabolite**	UTP	UTP	UTP	UTP	UTP	UTP	UTP	UTP	UTP	UTP
**Strain**	WT/vector	*prs1 prs3/vector*	*prs1 prs3/PRPS1*	*prs1 prs3/PRPS2*	*prs1 prs3/PRSA*	WT/vector	*prs1 prs3/vector*	*prs1 prs3/PRPS1*	*prs1 prs3/PRPS2*	*prs1 prs3/PrsA*
sample 1	2,61	1,23	1,35	1,57	1,28	0,79	0,37	0,41	0,48	0,39
sample 2	2,65	1,22	1,28	1,28	1,18	0,80	0,37	0,39	0,39	0,36
sample 3	2,49	1,34	1,40	1,52	1,07	0,75	0,41	0,42	0,46	0,32
sample 4	2,39	1,23	1,43	1,24	1,14	0,72	0,37	0,43	0,38	0,35
sample 5	2,68	1,15	1,25	1,38	1,26	0,81	0,35	0,38	0,42	0,38
sample 6	2,55	1,13	1,26	1,31	1,33	0,77	0,34	0,38	0,40	0,40
**Mean**	2,56	1,22	1,33	1,38	1,21	0,78	0,37	0,40	0,42	0,37
**SD**	0,11	0,07	0,08	0,13	0,10	0,03	0,02	0,02	0,04	0,03
Unpaired t-test *vs prs1 prs3*	1,6E-09		0,03	0,03	0,90	1,6E-09		0,03	0,03	0,90

E: [CTP] in mM

						mM	mM	mM	mM	mM
	Peak area	Peak area	Peak area	Peak area	Peak area	in cells	in cells	in cells	in cells	in cells
**Metabolite**	CTP	CTP	CTP	CTP	CTP	CTP	CTP	CTP	CTP	CTP
**Strain**	WT/vector	*prs1 prs3/vector*	*prs1 prs3/PRPS1*	*prs1 prs3/PRPS2*	*prs1 prs3/PRSA*	WT/vector	*prs1 prs3/vector*	*prs1 prs3/PRPS1*	*prs1 prs3/PRPS2*	*prs1 prs3/PrsA*
sample 1	1,51	0,73	0,85	0,93	0,81	0,51	0,25	0,29	0,31	0,27
sample 2	1,45	0,72	0,76	0,82	0,76	0,49	0,24	0,26	0,28	0,26
sample 3	1,47	0,78	0,89	0,88	0,82	0,49	0,26	0,30	0,30	0,28
sample 4	1,49	0,75	0,78	0,89	0,78	0,50	0,25	0,26	0,30	0,26
sample 5	1,53	0,74	0,80	0,89	0,84	0,52	0,25	0,27	0,30	0,28
sample 6	1,59	0,70	0,81	0,89	0,86	0,53	0,24	0,27	0,30	0,29
**Mean**	1,51	0,74	0,81	0,89	0,81	0,51	0,25	0,27	0,30	0,27
**SD**	0,05	0,03	0,05	0,03	0,04	0,02	0,01	0,02	0,01	0,01
Unpaired t-test *vs prs1 prs3*	1,3E-09		8,6E-03	1,1E-05	3,1E-03	1,3E-09		8,6E-03	1,1E-05	3,1E-03

H: [NAD^{+}] + [NADH] in mM

						mM	mM	mM	mM	mM
	µM in sample	µM in sample	µM in sample	µM in sample	µM in sample	in cells	in cells	in cells	in cells	in cells
**Metabolite**	NAD(H)	NAD(H)	NAD(H)	NAD(H)	NAD(H)	NAD(H)	NAD(H)	NAD(H)	NAD(H)	NAD(H)
**Strain**	WT/vector	*prs1 prs3/vector*	*prs1 prs3/PRPS1*	*prs1 prs3/PRPS2*	*prs1 prs3/PRSA*	WT/vector	*prs1 prs3/vector*	*prs1 prs3/PRPS1*	*prs1 prs3/PRPS2*	*prs1 prs3/PrsA*
sample 1	46,28	20,05	27,82	33,60	29,32	1,39	0,60	0,83	1,01	0,88
sample 2	46,33	20,33	29,81	30,55	31,08	1,39	0,61	0,89	0,92	0,93
sample 3	45,29	22,70	31,95	34,64	31,34	1,36	0,68	0,96	1,04	0,94
sample 4	43,84	20,97	31,74	31,31	32,05	1,32	0,63	0,95	0,94	0,96
sample 5	47,69	22,62	31,83	32,36	31,63	1,43	0,68	0,95	0,97	0,95
sample 6	48,35	23,10	32,81	33,30	33,38	1,45	0,69	0,98	1,00	1,00
**Mean**	46,30	21,63	30,99	32,63	31,47	1,39	0,65	0,93	0,98	0,94
**SD**	1,63	1,34	1,84	1,52	1,33	0,05	0,04	0,06	0,05	0,04
Unpaired t-test *vs prs1 prs3*	1,2E-10		3,0E-06	1,3E-07	1,6E-07	1,2E-10		3,0E-06	1,3E-07	1,6E-07

Table S9: Raw data Figure 6

B : Doubling Time

	Doubling Time (min)	Doubling Time (min)	Doubling Time (min)	Doubling Time (min)
	WT	*prs1 prs3*	WT + Supplements	*prs1 prs3* + supplements
sample 1	98,7	333,7	85,8	103,4
sample 2	94,6	317,2	92,7	110,4
sample 3	97,5	339,1	91,9	102,4
Unpaired t-test *vs* WT		5,6E-04	0,07	0,06
Unpaired t-test vs *prs1prs3*	5,6E-04		2,5E-04	2,1E-04

C: [PRPP] in mM

					mM	mM	mM	mM
	Peak area	Peak area	Peak area	Peak area	in cells	in cells	in cells	in cells
Metabolite	PRPP	PRPP	PRPP	PRPP	PRPP	PRPP	PRPP	PRPP
Strain	WT	*prs1 prs3*	WT + Supplements	*prs1 prs3* + supplements	WT	*prs1 prs3*	WT + Supplements	*prs1 prs3* + supplements
sample 1	0,0220	0,0030	0,0200	0,0002	0,117	0,016	0,107	0,001
sample 2	0,0220	0,0024	0,0210	0,0003	0,117	0,013	0,112	0,002
sample 3	0,0199	0,0029	0,0201	0,0001	0,106	0,015	0,107	0,001
sample 4	0,0201	0,0029	0,0177	0,0001	0,107	0,015	0,094	0,001
**Mean**	0,0210	0,0028	0,0197	0,0002	0,112	0,015	0,105	0,001
**SD**	0,0012	0,0003	0,0014	0,0001	0,006	0,001	0,008	0,001
Unpaired t-test vsWT		3,4E-05	0,21	4,3E-05		3,4E-05	0,21	4,3E-05
Unpaired t-test vs *prs1prs3*	3,4E-05		1,0E-04	8,6E-05	3,4E-05		1,0E-04	8,6E-05

C: [ATP] in mM

					mM	mM	mM	mM
	Peak area	Peak area	Peak area	Peak area	in cells	in cells	in cells	in cells
Metabolite	ATP	ATP	ATP	ATP	ATP	ATP	ATP	ATP
Strain	WT	*prs1 prs3*	WT + Supplements	*prs1 prs3* + supplements	WT	*prs1 prs3*	WT + Supplements	*prs1 prs3* + supplements
sample 1	14,6	3,2	13,9	21,5	3,21	0,70	3,07	4,74
sample 2	14,5	3,0	14,5	21,6	3,19	0,67	3,19	4,76
sample 3	14,9	4,3	14,1	21,4	3,29	0,96	3,11	4,72
sample 4	15,1	4,1	13,3	20,8	3,33	0,90	2,94	4,58
**Mean**	14,8	3,6	14,0	21,3	3,26	0,80	3,08	4,70
**SD**	0,3	0,6	0,5	0,4	0,07	0,14	0,10	0,08
Unpaired t-test vsWT		3,8E-06	0,03	3,1E-07		3,8E-06	3,2E-02	3,1E-07
Unpaired t-test vs *prs1prs3*	3,8E-06		6,7E-07	1,3E-07	3,8E-06		6,7E-07	1,3E-07

E: [GTP] in mM

					mM	mM	mM	mM
	Peak area	Peak area	Peak area	Peak area	in cells	in cells	in cells	in cells
Metabolite	GTP	GTP	GTP	GTP	GTP	GTP	GTP	GTP
Strain	WT	*prs1 prs3*	WT + Supplements	*prs1 prs3* + supplements	WT	*prs1 prs3*	WT + Supplements	*prs1 prs3* + supplements
sample 1	2,87	0,71	2,90	3,24	0,77	0,19	0,77	0,86
sample 2	2,79	0,69	2,96	3,36	0,74	0,18	0,79	0,90
sample 3	2,80	0,98	2,85	3,28	0,74	0,26	0,76	0,87
sample 4	2,69	0,91	2,67	3,23	0,72	0,24	0,71	0,86
**Mean**	2,8	0,8	2,8	3,3	0,74	0,22	0,76	0,87
**SD**	0,1	0,1	0,1	0,1	0,02	0,04	0,03	0,02
Unpaired t-test vsWT		5,3E-06	0,48	6,7E-05		5,3E-06	0,48	6,7E-05
Unpaired t-test vs *prs1prs3*	5,3E-06		7,9E-07	5,0E-06	5,3E-06		7,9E-07	5,0E-06

F: [UTP] in mM

					mM	mM	mM	mM
	Peak area	Peak area	Peak area	Peak area	in cells	in cells	in cells	in cells
Metabolite	UTP	UTP	UTP	UTP	UTP	UTP	UTP	UTP
Strain	WT	*prs1 prs3*	WT + Supplements	*prs1 prs3* + supplements	WT	*prs1 prs3*	WT + Supplements	*prs1 prs3* + supplements
sample 1	1,75	0,42	2,77	2,07	0,71	0,17	1,12	0,84
sample 2	1,63	0,39	2,64	2,10	0,66	0,16	1,07	0,85
sample 3	1,66	0,53	2,60	2,10	0,67	0,21	1,05	0,85
sample 4	1,66	0,52	2,48	2,08	0,67	0,21	1,00	0,84
**Mean**	1,7	0,5	2,6	2,1	0,68	0,19	1,06	0,84
**SD**	0,1	0,1	0,1	0,0	0,02	0,03	0,05	0,01
Unpaired t-test vsWT		2,1E-07	1,0E-04	3,4E-04		2,1E-07	1,0E-04	3,4E-04
Unpaired t-test vs *prs1prs3*	2,1E-07		1,3E-06	8,8E-06	2,1E-07		1,3E-06	8,8E-06

G: [CTP] in mM

					mM	mM	mM	mM
	Peak area	Peak area	Peak area	Peak area	in cells	in cells	in cells	in cells
Metabolite	CTP	CTP	CTP	CTP	CTP	CTP	CTP	CTP
Strain	WT	*prs1 prs3*	WT + Supplements	*prs1 prs3* + supplements	WT	*prs1 prs3*	WT + Supplements	*prs1 prs3* + supplements
sample 1	1,00	0,28	1,21	1,39	0,45	0,13	0,54	0,62
sample 2	0,99	0,27	1,23	1,39	0,44	0,12	0,55	0,62
sample 3	1,02	0,39	1,21	1,39	0,46	0,17	0,54	0,63
sample 4	1,02	0,35	1,14	1,37	0,46	0,16	0,51	0,62
**Mean**	1,0	0,3	1,2	1,4	0,45	0,14	0,54	0,62
**SD**	0,0	0,1	0,0	0,0	0,01	0,03	0,02	0,00
Unpaired t-test vsWT		6,8E-05	6,8E-04	6,3E-08		6,8E-05	6,8E-04	6,3E-08
Unpaired t-test vs *prs1prs3*	6,8E-05		1,1E-06	2,6E-05	6,8E-05		1,1E-06	2,6E-05

H: $[NAD^{+}]$ + [NADH] in mM

					mM	mM	mM	mM
	µM in extract	µM in extract	µM in extract	µM in extract	in cells	in cells	in cells	in cells
Metabolite	NAD(H)	NAD(H)	NAD(H)	NAD(H)	NAD(H)	NAD(H)	NAD(H)	NAD(H)
Strain	WT	*prs1 prs3*	WT + Supplements	*prs1 prs3* + supplements	WT	*prs1 prs3*	WT + Supplements	*prs1 prs3* + supplements
sample 1	63,12	18,54	63,09	42,42	1,26	0,37	1,26	0,85
sample 2	63,36	21,63	66,20	42,15	1,27	0,43	1,32	0,84
sample 3	64,56	27,24	58,57	47,46	1,29	0,54	1,17	0,95
sample 4	68,28	23,25	62,40	45,30	1,37	0,46	1,25	0,91
**Mean**	64,8	22,7	62,6	44,3	1,30	0,45	1,25	0,89
**SD**	2,4	3,6	3,1	2,5	0,05	0,07	0,06	0,05
Unpaired t-test vsWT		4,7E-06	0,30	2,3E-05		4,7E-06	0,30	2,3E-05
Unpaired t-test vs *prs1prs3*	4,7E-06		3,6E-06	1,3E-04	4,7E-06		3,6E-06	1,3E-04

Table S10: Raw data Figure 7

C: Population doubling time (min)

	Doubling Time (min) Wild Type	Doubling Time (min) *prs1 prs3 PRS5 WT*	Doubling Time (min) *prs1 prs3 PRS5* P110S	Doubling Time (min) *prs1 prs3 PRS5* R311I	Doubling Time (min) *prs1 prs3 PRS5* D401N
Strain 1	85,9	221,6	94,6	160,2	103,1
Strain 2	98,8	272,1	106,2	183,4	120,8
Strain 3	91,9	244,2	100,1	175,3	111,3
Strain 4	94,1	258,5	103,4	163,4	108,9
Unpaired t-test vs *prs1 prs3*	4,1E-04		5,3E-04	2,0E-03	4,2E-04

E: [PRPP] in mM

	Peak area	Peak area	Peak area	Peak area	Peak area	mM in cells	mM in cells	mM in cells	mM in cells	mM in cells
Metabolite	PRPP	PRPP	PRPP	PRPP	PRPP	PRPP	PRPP	PRPP	PRPP	PRPP
Strain	Wild Type	*prs1 prs3 PRS5 WT*	*prs1 prs3 PRS5* P110S	*prs1 prs3 PRS5* R311I	*prs1 prs3 PRS5* D401N	Wild Type	*prs1 prs3 PRS5 WT*	*prs1 prs3 PRS5* P110S	*prs1 prs3 PRS5* R311I	*prs1 prs3 PRS5* D401N
sample 1	0,037	0,006	0,014	0,008	0,012	0,1480	0,0240	0,0560	0,0320	0,0480
sample 2	0,041	0,005	0,013	0,011	0,011	0,1640	0,0200	0,0520	0,0440	0,0440
sample 3	0,036	0,004	0,013	0,009	0,010	0,1440	0,0160	0,0520	0,0360	0,0400
sample 4	0,037	0,005	0,014	0,010	0,012	0,1480	0,0200	0,0560	0,0400	0,0480
sample 5	0,038	0,006	0,012	0,010	0,011	0,1520	0,0240	0,0480	0,0400	0,0440
sample 6	0,037	0,005	0,013	0,011	0,013	0,1480	0,0200	0,0520	0,0440	0,0520
**Mean**	0,038	0,005	0,013	0,010	0,012	0,15	0,02	0,05	0,04	0,05
**SD**	0,002	0,001	0,001	0,001	0,001	0,01	0,00	0,00	0,00	0,00
Unpaired t-test vs *prs1 prs3*	1,9E-09		4,8E-09	2,4E-05	7,1E-07	1,9E-09		4,8E-09	2,4E-05	7,1E-07

F: [ATP] in mM

	Peak area	Peak area	Peak area	Peak area	Peak area	mM in cells	mM in cells	mM in cells	mM in cells	mM in cells
Metabolite	ATP	ATP	ATP	ATP	ATP	ATP	ATP	ATP	ATP	ATP
Strain	Wild Type	*prs1 prs3 PRS5 WT*	*prs1 prs3 PRS5* P110S	*prs1 prs3 PRS5* R311I	*prs1 prs3 PRS5* D401N	Wild Type	*prs1 prs3 PRS5 WT*	*prs1 prs3 PRS5* P110S	*prs1 prs3 PRS5* R311I	*prs1 prs3 PRS5* D401N
sample 1	20,10	9,88	15,47	13,24	15,90	3,94	1,94	3,03	2,60	3,12
sample 2	20,32	9,92	16,00	15,57	15,75	3,98	1,94	3,14	3,05	3,09
sample 3	20,23	10,26	15,98	14,19	15,78	3,97	2,01	3,13	2,78	3,09
sample 4	21,43	9,84	15,91	13,26	15,74	4,20	1,93	3,12	2,60	3,09
sample 5	19,74	9,91	15,29	13,38	15,02	3,87	1,94	3,00	2,62	2,95
sample 6	21,05	9,27	15,09	13,28	15,56	4,13	1,82	2,96	2,60	3,05
**Mean**	20,48	9,85	15,62	13,82	15,63	4,02	1,93	3,06	2,71	3,06
**SD**	0,63	0,32	0,39	0,93	0,32	0,12	0,06	0,08	0,18	0,06
Unpaired t-test vs *prs1 prs3*	1,2E-09		1,6E-10	5,2E-05	2,5E-11	1,2E-09		1,6E-10	5,2E-05	2,5E-11

G: [GTP] in mM

	Peak area	Peak area	Peak area	Peak area	Peak area	mM in cells	mM in cells	mM in cells	mM in cells	mM in cells
Metabolite	GTP	GTP	GTP	GTP	GTP	GTP	GTP	GTP	GTP	GTP
Strain	Wild Type	*prs1 prs3 PRS5 WT*	*prs1 prs3 PRS5* P110S	*prs1 prs3 PRS5* R311I	*prs1 prs3 PRS5* D401N	Wild Type	*prs1 prs3 PRS5 WT*	*prs1 prs3 PRS5* P110S	*prs1 prs3 PRS5* R311I	*prs1 prs3 PRS5* D401N
sample 1	3,89	0,81	1,59	1,31	1,67	0,91	0,19	0,37	0,31	0,39
sample 2	4,01	0,82	1,71	1,47	1,64	0,94	0,19	0,40	0,34	0,38
sample 3	4,07	0,84	1,72	1,34	1,69	0,95	0,19	0,40	0,31	0,39
sample 4	4,36	0,79	1,74	1,38	1,64	1,02	0,18	0,41	0,32	0,38
sample 5	3,91	0,81	1,68	1,27	1,54	0,91	0,19	0,39	0,30	0,36
sample 6	4,23	0,78	1,57	1,26	1,74	0,99	0,18	0,36	0,29	0,41
**Mean**	4,08	0,81	1,67	1,34	1,65	0,95	0,19	0,39	0,31	0,39
**SD**	0,18	0,02	0,07	0,08	0,07	0,04	0,00	0,02	0,02	0,02
Unpaired t-test vs *prs1 prs3*	9,3E-08		2,6E-07	5,8E-06	1,2E-07	9,3E-08		2,6E-07	5,8E-06	1,2E-07

H: [UTP] in mM

	Peak area	Peak area	Peak area	Peak area	Peak area	mM in cells	mM in cells	mM in cells	mM in cells	mM in cells
Metabolite	UTP	UTP	UTP	UTP	UTP	UTP	UTP	UTP	UTP	UTP
Strain	Wild Type	*prs1 prs3 PRS5 WT*	*prs1 prs3 PRS5* P110S	*prs1 prs3 PRS5* R311I	*prs1 prs3 PRS5* D401N	Wild Type	*prs1 prs3 PRS5 WT*	*prs1 prs3 PRS5* P110S	*prs1 prs3 PRS5* R311I	*prs1 prs3 PRS5* D401N
sample 1	3,16	1,26	1,71	2,00	1,85	1,12	0,44	0,61	0,71	0,66
sample 2	3,29	1,25	1,82	1,96	1,97	1,16	0,44	0,64	0,69	0,70
sample 3	3,26	1,20	1,87	1,89		1,15	0,42	0,66	0,67	
sample 4	3,49	1,23	1,80	1,90	2,29	1,23	0,43	0,64	0,67	0,81
sample 5	3,39	1,20	1,75	1,67	1,93	1,20	0,42	0,62	0,59	0,68
sample 6	3,56	1,20	1,67	1,90	2,04	1,26	0,43	0,59	0,67	0,72
**Mean**	3,36	1,22	1,77	1,89	2,02	1,19	0,43	0,63	0,67	0,71
**SD**	0,15	0,03	0,08	0,11	0,17	0,05	0,01	0,03	0,04	0,06
Unpaired t-test vs *prs1 prs3*	2,1E-07		2,2E-06	1,7E-05	3,8E-04	2,1E-07		2,2E-06	1,7E-05	3,8E-04

I: [CTP] in mM

	Peak area	Peak area	Peak area	Peak area	Peak area	mM in cells	mM in cells	mM in cells	mM in cells	mM in cells
Metabolite	CTP	CTP	CTP	CTP	CTP	CTP	CTP	CTP	CTP	CTP
Strain	Wild Type	*prs1 prs3 PRS5 WT*	*prs1 prs3 PRS5* P110S	*prs1 prs3 PRS5* R311I	*prs1 prs3 PRS5* D401N	Wild Type	*prs1 prs3 PRS5 WT*	*prs1 prs3 PRS5* P110S	*prs1 prs3 PRS5* R311I	*prs1 prs3 PRS5* D401N
sample 1	2,81	1,01	1,43	1,47	1,46	0,79	0,28	0,40	0,41	0,41
sample 2	2,82	1,00	1,46	1,73	1,48	0,79	0,28	0,41	0,49	0,41
sample 3	2,81	0,99	1,50	1,58	1,37	0,79	0,28	0,42	0,44	0,38
sample 4	2,91	0,98	1,45	1,55	1,41	0,82	0,27	0,41	0,43	0,40
sample 5	2,73	0,99	1,38	1,53	1,35	0,77	0,28	0,39	0,43	0,38
sample 6	2,91	0,93	1,45	1,52	1,37	0,82	0,26	0,41	0,43	0,38
**Mean**	2,83	0,98	1,44	1,56	1,41	0,79	0,28	0,41	0,44	0,39
**SD**	0,07	0,03	0,04	0,09	0,05	0,02	0,01	0,01	0,03	0,01
Unpaired t-test vs *prs1 prs3*	3,2E-10		2,6E-09	7,0E-06	3,1E-07	3,2E-10		2,6E-09	7,0E-06	3,1E-07

J: [NAD(H)] in mM

	µM in sample	µM in sample	µM in sample	µM in sample	µM in sample	mM in cells	mM in cells	mM in cells	mM in cells	mM in cells
Metabolite	NAD(H)	NAD(H)	NAD(H)	NAD(H)	NAD(H)	NAD(H)	NAD(H)	NAD(H)	NAD(H)	NAD(H)
Strain	Wild Type	*prs1 prs3 PRS5 WT*	*prs1 prs3 PRS5* P110S	*prs1 prs3 PRS5* R311I	*prs1 prs3 PRS5* D401N	Wild Type	*prs1 prs3 PRS5 WT*	*prs1 prs3 PRS5* P110S	*prs1 prs3 PRS5* R311I	*prs1 prs3 PRS5* D401N
sample 1	88,4	47,3	70,5	64,7	64,2	2,53	1,35	2,01	1,85	1,83
sample 2	95,8	52,4	72,8	71,4	77,0	2,74	1,50	2,08	2,04	2,20
sample 3	101,7	46,5	80,4	70,7	74,9	2,91	1,33	2,30	2,02	2,14
sample 4	102,1	48,5	77,9	67,4	74,1	2,92	1,39	2,23	1,93	2,12
sample 5	96,1	49,2	72,8	65,0	70,4	2,75	1,41	2,08	1,86	2,01
sample 6	95,3	48,8	69,3	61,6	81,0	2,72	1,39	1,98	1,76	2,31
**Mean**	96,56	48,80	73,95	66,81	73,61	2,76	1,39	2,11	1,91	2,10
**SD**	5,01	2,03	4,32	3,77	5,79	0,14	0,06	0,12	0,11	0,17
Unpaired t-test vs *prs1 prs3*	2,3E-07		3,4E-06	9,2E-06	4,9E-05	2,3E-07		3,4E-06	9,2E-06	4,9E-05

Table S11: Raw data Figure 8 (Part 1/2)

A: [PRPP] in mM

	Peak area	Peak area	Peak area	Peak area	mM in cells	mM in cells	mM in cells	mM in cells
**Metabolite**	PRPP	PRPP	PRPP	PRPP	PRPP	PRPP	PRPP	PRPP
**Strain**	Control	PRPS1 WT	PRPS1 V142L	PRPS1 A190V	Control	PRPS1 WT	PRPS1 V142L	PRPS1 A190V
sample 1	0,028	0,027		0,044	0,112	0,108		0,176
sample 2	0,030	0,026	0,038	0,043	0,120	0,104	0,152	0,172
sample 3	0,030	0,026	0,043	0,051	0,120	0,104	0,172	0,204
sample 4	0,033	0,031	0,045	0,049	0,132	0,124	0,180	0,196
sample 5	0,029	0,028	0,041	0,047	0,116	0,112	0,164	0,188
sample 6	0,034	0,029	0,043	0,051	0,136	0,116	0,172	0,204
**Mean**	0,031	0,028	0,042	0,048	0,123	0,111	0,168	0,190
**SD**	0,002	0,002	0,003	0,003	0,009	0,008	0,011	0,014
Unpaired t-test vs empty vector		4,6E-02	6,6E-05	4,6E-06		4,6E-02	6,6E-05	4,6E-06

B: [ATP] in mM

	Peak area	Peak area	Peak area	Peak area	mM in cells	mM in cells	mM in cells	mM in cells
**Metabolite**	ATP	ATP	ATP	ATP	ATP	ATP	ATP	ATP
**Strain**	Control	PRPS1 WT	PRPS1 V142L	PRPS1 A190V	Control	PRPS1 WT	PRPS1 V142L	PRPS1 A190V
sample 1	24,6390	22,5630		28,8390	3,623	3,318		4,241
sample 2	23,3730	23,1000	27,0610	29,3410	3,437	3,397	3,980	4,315
sample 3	24,9750	20,3550	26,7010	26,6500	3,673	2,993	3,927	3,919
sample 4	23,4690	22,7800	28,3900	29,3420	3,451	3,350	4,175	4,315
sample 5	23,0900	22,7100	26,0100	29,0590	3,396	3,340	3,825	4,273
sample 6	24,0700	22,2500	27,5400	29,3300	3,540	3,272	4,050	4,313
**Mean**	23,94	22,29	27,14	28,76	3,520	3,278	3,991	4,229
**SD**	0,75	0,99	0,89	1,05	0,111	0,145	0,131	0,155
Unpaired t-test vs empty vector		9,7E-03	2,3E-04	7,3E-06		9,7E-03	2,3E-04	7,3E-06

C: [GTP] in mM

	Peak area	Peak area	Peak area	Peak area	mM in cells	mM in cells	mM in cells	mM in cells
**Metabolite**	GTP	GTP	GTP	GTP	GTP	GTP	GTP	GTP
**Strain**	Control	PRPS1 WT	PRPS1 V142L	PRPS1 A190V	Control	PRPS1 WT	PRPS1 V142L	PRPS1 A190V
sample 1	4,6890	4,0090		5,6600	0,937	0,801		1,130
sample 2	4,3900	4,1480	5,2100	5,7100	0,877	0,828	1,041	1,140
sample 3	4,7800	3,7300	5,2800	4,8800	0,955	0,745	1,055	0,975
sample 4	4,4390	4,3300	5,5500	5,7800	0,887	0,865	1,109	1,154
sample 5	4,4700	4,2500	5,1900	5,7400	0,893	0,849	1,037	1,146
sample 6	4,5400	4,4500	5,5700	5,9100	0,907	0,889	1,113	1,180
**Mean**	4,55	4,15	5,36	5,61	0,909	0,829	1,071	1,121
**SD**	0,15	0,26	0,19	0,37	0,031	0,051	0,037	0,074
Unpaired t-test vs empty vector		0,01	6,1E-05	4,1E-04		0,01	6,1E-05	4,1E-04

D: [UTP] in mM

	Peak area	Peak area	Peak area	Peak area	mM in cells	mM in cells	mM in cells	mM in cells
**Metabolite**	UTP	UTP	UTP	UTP	UTP	UTP	UTP	UTP
**Strain**	Control	PRPS1 WT	PRPS1 V142L	PRPS1 A190V	Control	PRPS1 WT	PRPS1 V142L	PRPS1 A190V
sample 1	2,5800	2,2500		3,1200	0,782	0,682		0,945
sample 2	2,4900	2,2900	2,9600	3,2200	0,755	0,694	0,897	0,976
sample 3	2,8080	2,0000	2,7310	3,0630	0,851	0,606	0,828	0,928
sample 4	2,5600	2,4010	3,0380	3,1770	0,776	0,728	0,921	0,963
sample 5	2,5020	2,2790	2,8300	3,2470	0,758	0,691	0,858	0,984
sample 6	2,5600	2,4040	3,0200	3,3300	0,776	0,728	0,915	1,009
**Mean**	2,58	2,27	2,92	3,19	0,783	0,688	0,884	0,968
**SD**	0,12	0,15	0,13	0,09	0,035	0,045	0,040	0,029
Unpaired t-test vs empty vector		2,5E-03	2,2E-03	2,2E-06		2,5E-03	2,2E-03	2,2E-06

E: [CTP] in mM

	Peak area	Peak area	Peak area	Peak area	mM in cells	mM in cells	mM in cells	mM in cells
**Metabolite**	CTP	CTP	CTP	CTP	CTP	CTP	CTP	CTP
**Strain**	Control	PRPS1 WT	PRPS1 V142L	PRPS1 A190V	Control	PRPS1 WT	PRPS1 V142L	PRPS1 A190V
sample 1	2,8200	2,5300	2,8090	3,3900	0,949	0,852		1,141
sample 2	2,7400	2,6300	3,1120	3,4100	0,923	0,886	1,048	1,148
sample 3	2,9300	2,3500	3,1440	2,9310	0,987	0,791	1,059	0,987
sample 4	2,8220	2,4460	3,2680	3,4760	0,950	0,824	1,100	1,170
sample 5	2,5950	2,5970	3,0070	3,4560	0,874	0,874	1,012	1,164
sample 6	2,8100	2,8330	3,0510	3,4440	0,946	0,954	1,027	1,160
**Mean**	2,79	2,56	3,07	3,35	0,938	0,863	1,049	1,128
**SD**	0,11	0,17	0,15	0,21	0,038	0,056	0,034	0,070
Unpaired t-test vs empty vector		0,02	5,7E-03	4,5E-04		0,02	6,0E-04	4,5E-04

F: [NAD(H)] in mM

	mM in cells	mM in cells	mM in cells	mM in cells
**Metabolite**	NAD(H)	NAD(H)	(NADH)	(NADH)
**Strain**	Control	PRPS1 WT	PRPS1 V142L	PRPS1 A190V
sample 1	1,9385	1,8617		2,4532
sample 2	1,9335	1,8906	2,3750	2,4179
sample 3	2,1209	1,7032	2,3389	2,2621
sample 4	2,1006	1,9662	2,4430	2,4590
sample 5	1,9955	1,9338	2,2827	2,5381
sample 6	2,0840	2,0527	2,3121	2,5029
**Mean**	2,03	1,90	2,35	2,44
**SD**	0,08	0,12	0,06	0,10
Unpaired t-test vs empty vector		0,06	4,8E-05	1,5E-05

Table S11: Raw data Figure 8 (Part 2/2)

G: Doubling time (min)

Metabolite	AEC	AEC	AEC	AEC
Strain	Control	PRPS1 WT	PRPS1 V142L	PRPS1 A190V
sample 1	81,330	84,140	90,740	89,890
sample 2	82,300	89,980	92,040	89,160
sample 3	84,660	85,500	94,490	91,150
sample 4	84,700	87,988	91,470	89,200
Mean	83,25	86,90	92,19	89,85
SD	1,70	2,60	1,63	0,93
Unpaired t-test vs empty vector		0,06	2,7E-04	1,4E-03

H: [SZMP] in mM

					mM	mM	mM	mM
	Peak area	Peak area	Peak area	Peak area	in cells	in cells	in cells	in cells
Metabolite	S-ZMP	S-ZMP	S-ZMP	S-ZMP	S-ZMP	S-ZMP	S-ZMP	S-ZMP
Strain	Control	PRPS1 WT	PRPS1 V142L	PRPS1 A190V	Control	PRPS1 WT	PRPS1 V142L	PRPS1 A190V
sample 1	0,0050	0,0030	0,0610	0,0460	0,00127	0,00076		0,01172
sample 2	0,0050	0,0040	0,0650	0,0520	0,00127	0,00102	0,01657	0,01325
sample 3	0,0050	0,0040	0,0610	0,0370	0,00127	0,00102	0,01555	0,00943
sample 4	0,0070	0,0060	0,0590	0,0550	0,00178	0,00153	0,01504	0,01402
sample 5	0,0060	0,0040	0,0580	0,0600	0,00153	0,00102	0,01478	0,01529
sample 6	0,0070	0,0050	0,0630	0,0570	0,00178	0,00127	0,01606	0,01453
Mean	0,0058	0,0043	0,0612	0,0512	0,001	0,001	0,016	0,013
SD	0,0010	0,0010	0,0026	0,0084	0,000	0,000	0,001	0,002
Unpaired t-test vs empty vector		0,03	1,5E-09	3,8E-05		0,03	2,7E-07	3,8E-05

I: [IMP] in mM

					mM	mM	mM	mM
	Peak area	Peak area	Peak area	Peak area	in cells	in cells	in cells	in cells
Metabolite	IMP	IMP	IMP	IMP	IMP	IMP	IMP	IMP
Strain	Control	PRPS1 WT	PRPS1 V142L	PRPS1 A190V	Control	PRPS1 WT	PRPS1 V142L	PRPS1 A190V
sample 1	0,139	0,092	0,591	0,693	0,062	0,041		0,310
sample 2	0,129	0,101	0,683	0,704	0,058	0,045	0,305	0,315
sample 3	0,145	0,110	0,721	0,596	0,065	0,049	0,322	0,266
sample 4	0,176	0,113	0,728	0,725	0,079	0,051	0,325	0,324
sample 5	0,150	0,126	0,690	0,761	0,067	0,056	0,308	0,340
sample 6	0,144	0,117	0,609	0,690	0,064	0,052	0,272	0,308
Mean	0,147	0,110	0,670	0,695	0,066	0,049	0,307	0,311
SD	0,016	0,012	0,057	0,055	0,007	0,005	0,021	0,025
Unpaired t-test vs empty vector		1,2E-03	1,0E-06	5,6E-07		1,2E-03	3,6E-06	5,6E-07

J: [Inosine] in mM

					mM	mM	mM	mM
	Peak area	Peak area	Peak area	Peak area	in cells	in cells	in cells	in cells
Metabolite	Inosine	Inosine	Inosine	Inosine	Inosine	Inosine	Inosine	Inosine
Strain	Control	PRPS1 WT	PRPS1 V142L	PRPS1 A190V	Control	PRPS1 WT	PRPS1 V142L	PRPS1 A190V
sample 1	0,819	0,686		9,787	0,159	0,133		1,902
sample 2	0,734	0,659	11,740	9,470	0,143	0,128	2,281	1,840
sample 3	0,827	0,553	13,050	7,730	0,161	0,107	2,536	1,502
sample 4	0,848	0,677	13,460	9,070	0,165	0,132	2,615	1,762
sample 5	0,931	0,742	10,480	10,440	0,181	0,144	2,036	2,028
sample 6	0,847	0,757	8,710	10,130	0,165	0,147	1,692	1,968
Mean	0,834	0,679	11,488	9,438	0,162	0,132	2,232	1,834
SD	0,063	0,073	1,945	0,965	0,012	0,014	0,378	0,188
Unpaired t-test vs empty vector		2,8E-03	2,5E-04	3,5E-06		2,8E-03	2,5E-04	3,5E-06

Table S12: Raw data Figure 9

D: [ATP] in mM

	Intracellular	Intracellular	Intracellular	Intracellular
	concentration	concentration	concentration	concentration
**Metabolite (mM)**	[ATP]	[ATP]	[ATP]	[ATP]
**Strain**	*ADE4* - adenine	*ade4Δ* - adenine	*ADE4* + adenine	*ade4Δ* + adenine
sample 1	3,500	0,390	3,581	3,390
sample 2	3,275	0,380	3,663	3,403
sample 3	3,405	0,350	4,003	3,568
sample 4	3,103	0,430	3,922	3,599
**Mean**	3,32	0,39	3,79	3,49
**SD**	0,17	0,03	0,20	0,11
Unpaired t-test vs *ADE4* -adenine		3,3E-05	1,3E-02	1,6E-01

E: [GTP] in mM

	Intracellular	Intracellular	Intracellular	Intracellular
	concentration	concentration	concentration	concentration
**Metabolite (mM)**	[GTP]	[GTP]	[GTP]	[GTP]
**Strain**	*ADE4* - adenine	*ade4Δ* - adenine	*ADE4* + adenine	*ade4Δ* + adenine
sample 1	0,955	0,374	0,841	0,823
sample 2	0,936	0,373	0,863	0,897
sample 3	0,884	0,359	0,964	0,881
sample 4	0,802	0,368	0,957	0,871
**Mean**	0,89	0,37	0,91	0,87
**SD**	0,07	0,01	0,06	0,03
Unpaired t-test vs *ADE4* -adenine		5,5E-04	8,1E-01	5,3E-01

F: [CTP] in mM

	Intracellular	Intracellular	Intracellular	Intracellular
	concentration	concentration	concentration	concentration
**Metabolite (mM)**	[CTP]	[CTP]	[CTP]	[CTP]
**Strain**	*ADE4* - adenine	*ade4Δ* - adenine	*ADE4* + adenine	*ade4Δ* + adenine
sample 1	0,475	0,246	0,453	0,467
sample 2	0,445	0,257	0,446	0,493
sample 3	0,449	0,251	0,526	0,495
sample 4	0,407	0,260	0,510	0,500
**Mean**	0,44	0,25	0,48	0,49
**SD**	0,03	0,01	0,04	0,01
Unpaired t-test vs *ADE4* -adenine		5,5E-04	1,6E-01	4,1E-02

G: [NAD[+] + NADH] in mM

	Intracellular	Intracellular	Intracellular	Intracellular
	concentration	concentration	concentration	concentration
**Metabolite (mM)**	[NAD(H)]	[NAD(H)]	[NAD(H)]	[NAD(H)]
**Strain**	*ADE4* - adenine	*ade4Δ* - adenine	*ADE4* + adenine	*ade4Δ* + adenine
sample 1	1,623	0,789	1,775	1,917
sample 2	1,935	0,785	1,940	1,974
sample 3	1,848	0,793	1,993	2,098
sample 4	1,952	0,818	2,114	2,233
**Mean**	1,84	0,80	1,96	2,06
**SD**	0,15	0,01	0,14	0,14
Unpaired t-test vs *ADE4* -adenine		7,6E-04	3,0E-01	8,1E-02

H: [UTP] in mM

	Intracellular	Intracellular	Intracellular	Intracellular
	concentration	concentration	concentration	concentration
**Metabolite (mM)**	[UTP]	[UTP]	[UTP]	[UTP]
**Strain**	*ADE4* - adenine	*ade4Δ* - adenine	*ADE4* + adenine	*ade4Δ* + adenine
sample 1	0,964	3,931	0,941	1,024
sample 2	0,928	4,052	1,042	1,116
sample 3	0,946	4,344	1,218	1,336
sample 4	0,958	4,276	1,269	1,228
**Mean**	0,95	4,15	1,12	1,18
**SD**	0,02	0,19	0,15	0,14
Unpaired t-test vs *ADE4* -adenine		5,4E-05	1,1E-01	4,3E-02

I: [UTP] in mM

	Intracellular	Intracellular
	concentration	concentration
**Metabolite (mM)**	[UTP]	[UTP]
	vector	Ade4 R328K
sample 1	0,955	0,504
sample 2	0,929	0,572
sample 3	0,838	0,574
sample 4	0,944	0,545
**Mean**	0,92	0,55
**SD**	0,05	0,03
Unpaired t-test vs vector		8,4E-05

J: [CTP] in mM

	Intracellular	Intracellular
	concentration	concentration
**Metabolite (mM)**	[CTP]	[CTP]
	vector	Ade4 R328K
sample 1	0,461	0,325
sample 2	0,451	0,358
sample 3	0,459	0,338
sample 4	0,449	0,327
**Mean**	0,45	0,34
**SD**	0,01	0,02
Unpaired t-test vs vector		1,5E-04

K: [ATP] in mM

	Intracellular	Intracellular
	concentration	concentration
**Metabolite (mM)**	[ATP]	[ATP]
	vector	Ade4 R328K
sample 1	3,889	3,557
sample 2	3,664	3,856
sample 3	3,671	3,834
sample 4	3,623	3,619
**Mean**	3,71	3,72
**SD**	0,12	0,15
Unpaired t-test vs vector		9,6E-01

L: [GTP] in mM

	Intracellular	Intracellular
	concentration	concentration
**Metabolite (mM)**	[GTP]	[GTP]
	vector	Ade4 R328K
sample 1	0,903	0,835
sample 2	0,896	0,911
sample 3	0,880	0,934
sample 4	0,871	0,874
**Mean**	0,89	0,89
**SD**	0,01	0,04
Unpaired t-test vs vector		9,7E-01

M: [ZMP] in mM

	Intracellular	Intracellular
	concentration	concentration
**Metabolite (mM)**	[ZMP]	[ZMP]
	vector	Ade4 R328K
sample 1	0,00059	0,01824
sample 2	0,00047	0,02136
sample 3	0,00044	0,02918
sample 4	0,00028	0,02572
**Mean**	0,00044	0,02363
**SD**	0,00013	0,00481
Unpaired t-test vs vector		2,4E-03

N: [Inosine] in mM

	Intracellular	Intracellular
	concentration	concentration
**Metabolite (mM)**	[Inosine]	[Inosine]
	vector	Ade4 R328K
sample 1	0,211	7,257
sample 2	0,186	7,850
sample 3	0,199	8,622
sample 4	0,183	8,285
**Mean**	0,19	8,00
**SD**	0,01	0,59
Unpaired t-test vs vector		1,2E-04

Table S13: Raw data Figure 10

A: [PRPP] in mM

	Peak area	Peak area	Peak area	Peak area	Peak area	mM in cells	mM in cells	mM in cells	mM in cells	mM in cells
**Metabolite**	**PRPP**	**PRPP**	**PRPP**	**PRPP**	**PRPP**	**PRPP**	**PRPP**	**PRPP**	**PRPP**	**PRPP**
**Strain**	**Control**	**PRPS1 WT**	**PRPS1 V142L**	**PRPS1 A190V**	**PRPS2 WT**	**Control**	**PRPS1 WT**	**PRPS1 V142L**	**PRPS1 A190V**	**PRPS2 WT**
sample 1	0,0011	0,0035	0,0056	0,0039	0,0028	0,026	0,081	0,130	0,090	0,065
sample 2	0,0013	0,0038	0,0074	0,0054	0,0027	0,030	0,088	0,172	0,125	0,063
sample 3	0,0013	0,0026	0,0061	0,0049	0,0025	0,030	0,060	0,141	0,114	0,058
sample 4	0,0012	0,0027	0,0051	0,0042	0,0018	0,028	0,063	0,118	0,097	0,042
**Mean**	0,0012	0,0032	0,0061	0,0046	0,0025	0,028	0,073	0,140	0,107	0,057
**SD**	0,0001	0,0006	0,0010	0,0007	0,0005	0,002	0,014	0,023	0,016	0,010
Unpaired t-test vs control		6,5E-03	2,1E-03	1,9E-03	0,01		6,5E-03	2,1E-03	1,9E-03	0,01

B: [ATP] in mM

	Peak area	Peak area	Peak area	Peak area	Peak area	mM in cells	mM in cells	mM in cells	mM in cells	mM in cells
**Metabolite**	**ATP**	**ATP**	**ATP**	**ATP**	**ATP**	**ATP**	**ATP**	**ATP**	**ATP**	**ATP**
**Strain**	**Control**	**PRPS1 WT**	**PRPS1 V142L**	**PRPS1 A190V**	**PRPS2 WT**	**Control**	**PRPS1 WT**	**PRPS1 V142L**	**PRPS1 A190V**	**PRPS2 WT**
sample 1	5,250	5,487	9,080	8,749	6,660	4,48	4,68	7,74	7,46	5,68
sample 2	5,820	5,865	9,240	9,650	6,730	4,96	5,00	7,88	8,23	5,74
sample 3	5,357	5,537	9,329	9,435	6,630	4,57	4,72	7,96	8,05	5,65
sample 4	5,712	5,996	9,388	9,633	6,497	4,87	5,11	8,01	8,21	5,54
**Mean**	5,53	5,72	9,26	9,37	6,63	4,72	4,88	7,90	7,99	5,65
**SD**	0,27	0,25	0,13	0,42	0,10	0,23	0,21	0,11	0,36	0,08
Unpaired t-test vs control		0,35	7,9E-06	1,8E-05	2,2E-03		0,35	7,9E-06	1,8E-05	2,2E-03

C: [GTP] in mM

	Peak area	Peak area	Peak area	Peak area	Peak area	mM in cells	mM in cells	mM in cells	mM in cells	mM in cells
**Metabolite**	**GTP**	**GTP**	**GTP**	**GTP**	**GTP**	**GTP**	**GTP**	**GTP**	**GTP**	**GTP**
**Strain**	**Control**	**PRPS1 WT**	**PRPS1 V142L**	**PRPS1 A190V**	**PRPS2 WT**	**Control**	**PRPS1 WT**	**PRPS1 V142L**	**PRPS1 A190V**	**PRPS2 WT**
sample 1	0,716	0,698	1,366	1,288	0,833	0,83	0,81	1,58	1,49	0,96
sample 2	0,638	0,702	1,420	1,395	0,848	0,74	0,81	1,64	1,62	0,98
sample 3	0,679	0,762	1,421	1,345	0,841	0,79	0,88	1,65	1,56	0,97
sample 4	0,714	0,687	1,428	1,361	0,822	0,83	0,80	1,65	1,58	0,95
**Mean**	0,69	0,71	1,41	1,35	0,84	0,80	0,82	1,63	1,56	0,97
**SD**	0,04	0,03	0,03	0,04	0,01	0,04	0,04	0,03	0,05	0,01
Unpaired t-test vs control		3,5E-01	1,5E-07	7,0E-07	2,4E-03		3,5E-01	1,5E-07	7,0E-07	2,4E-03

D: [UTP] in mM

	Peak area	Peak area	Peak area	Peak area	Peak area	mM in cells	mM in cells	mM in cells	mM in cells	mM in cells
**Metabolite**	**UTP**	**UTP**	**UTP**	**UTP**	**UTP**	**UTP**	**UTP**	**UTP**	**UTP**	**UTP**
**Strain**	**Control**	**PRPS1 WT**	**PRPS1 V142L**	**PRPS1 A190V**	**PRPS2 WT**	**Control**	**PRPS1 WT**	**PRPS1 V142L**	**PRPS1 A190V**	**PRPS2 WT**
sample 1	0,6746	0,6628	1,3801	1,2011	0,8751	1,19	1,16	2,43	2,11	1,54
sample 2	0,6142	0,6725	1,4262	1,3295	0,8663	1,08	1,18	2,51	2,34	1,52
sample 3	0,6203	0,7168	1,4325	1,2780	0,8759	1,09	1,26	2,52	2,25	1,54
sample 4	0,6580	0,7145	1,4818	1,3214	0,8476	1,16	1,26	2,60	2,32	1,49
**Mean**	0,64	0,69	1,43	1,28	0,87	1,13	1,22	2,51	2,25	1,52
**SD**	0,03	0,03	0,04	0,06	0,01	0,05	0,05	0,07	0,10	0,02
Unpaired t-test vs control		0,05	2,8E-07	1,9E-05	1,2E-04		0,05	2,8E-07	1,9E-05	1,2E-04

E: [CTP] in mM

	Peak area	Peak area	Peak area	Peak area	Peak area	mM in cells	mM in cells	mM in cells	mM in cells	mM in cells
**Metabolite**	**CTP**	**CTP**	**CTP**	**CTP**	**CTP**	**CTP**	**CTP**	**CTP**	**CTP**	**CTP**
**Strain**	**Control**	**PRPS1 WT**	**PRPS1 V142L**	**PRPS1 A190V**	**PRPS2 WT**	**Control**	**PRPS1 WT**	**PRPS1 V142L**	**PRPS1 A190V**	**PRPS2 WT**
sample 1	0,3081	0,3101	0,6045	0,5360	0,4052	0,60	0,61	1,18	1,05	0,79
sample 2	0,2803	0,3202	0,6372	0,5904	0,4051	0,55	0,63	1,24	1,15	0,79
sample 3	0,2888	0,3327	0,6358	0,5715	0,4033	0,56	0,65	1,24	1,12	0,79
sample 4	0,3084	0,3347	0,6383	0,5908	0,3844	0,60	0,65	1,25	1,15	0,75
**Mean**	0,30	0,32	0,63	0,57	0,40	0,58	0,63	1,23	1,12	0,78
**SD**	0,01	0,01	0,02	0,03	0,01	0,03	0,02	0,03	0,05	0,02
Unpaired t-test vs control		0,02	1,0E-07	1,4E-05	4,3E-05		0,02	1,0E-07	1,4E-05	4,3E-05

F: [NAD(H)] in mM

	µM in sample	µM in sample	µM in sample	µM in sample	µM in sample	mM in cells	mM in cells	mM in cells	mM in cells	mM in cells
**Metabolite**	**NAD(H)**	**NAD(H)**	**NAD(H)**	**NAD(H)**	**NAD(H)**	**NAD(H)**	**NAD(H)**	**NAD(H)**	**NAD(H)**	**NAD(H)**
**Strain**	**Control**	**PRPS1 WT**	**PRPS1 V142L**	**PRPS1 A190V**	**PRPS2 WT**	**Control**	**PRPS1 WT**	**PRPS1 V142L**	**PRPS1 A190V**	**PRPS2 WT**
sample 1	6,3536	5,2928	7,5551	7,8023	7,5133	2,21	1,84	2,63	2,71	2,61
sample 2	5,6084	5,4943	6,9772	7,9049	6,7833	1,95	1,91	2,43	2,75	2,36
sample 3	5,6958	5,9810	6,9392	8,0038	6,8289	1,98	2,08	2,41	2,78	2,38
sample 4	6,1217	5,5475	7,5475	8,2510	7,1027	2,13	1,93	2,63	2,87	2,47
**Mean**	5,94	5,58	7,25	7,99	7,06	2,07	1,94	2,52	2,78	2,46
**SD**	0,35	0,29	0,34	0,19	0,34	0,12	0,10	0,12	0,07	0,12
Unpaired t-test vs control		1,6E-01	1,8E-03	2,4E-04	3,8E-03		1,6E-01	1,8E-03	2,4E-04	3,8E-03

G: Cell proliferation

	Cells/cm² x10-3	Cells/cm² x10-3	Cells/cm² x10-3	Cells/cm² x10-3	Cells/cm² x10-3
	**Control**	**PRPS1 WT**	**PRPS1 V142L**	**PRPS1 A190V**	**PRPS2 WT**
Strain 1	76,4	61,9	54,5	46,5	33,4
Strain 2	78,7	59,1	48,0	45,8	37,3
Strain 3	73,4	57,1	49,1	44,8	39,1
Unpaired t-test vs control		1,3E-03	7,6E-04	1,1E-03	6,6E-05

Table S14: Raw data Figure 11

HCT116

A: [ATP] in mM

	Peak area	Peak area	mM in cells	mM in cells
**Metabolite**	**ATP**	**ATP**	**ATP**	**ATP**
**Extracellular Purine**	**None**	**Adenine**	**None**	**Adenine**
sample 1	60,12	97,43	3,00	4,87
sample 2	67,33	101,23	3,36	5,06
sample 3	66,13	99,63	3,30	4,98
**Mean**	64,53	99,43	3,22	4,97
**SD**	3,87	1,91	0,19	0,10
Unpaired t-test vs control		9,0E-04		9,0E-04

B: GTP relative content

	Peak area	Peak area	mM in cells	mM in cells
**Metabolite**	**GTP**	**GTP**	**GTP**	**GTP**
**Extracellular Purine**	**None**	**Adenine**	**None**	**Adenine**
sample 1	5,48	3,43	0,37	0,24
sample 2	6,37	3,48	0,43	0,24
sample 3	6,07	3,51	0,41	0,25
**Mean**	5,97	3,47	0,40	0,24
**SD**	0,45	0,04	0,03	0,01
Unpaired t-test vs control		9,9E-03		9,9E-03

C: UTP relative content

	Peak area	Peak area	mM in cells	mM in cells
**Metabolite**	**UTP**	**UTP**	**UTP**	**UTP**
**Extracellular Purine**	**None**	**Adenine**	**None**	**Adenine**
sample 1	9,48	6,29	0,98	0,67
sample 2	10,54	7,84	1,09	0,84
sample 3	10,52	6,47	1,08	0,69
**Mean**	10,18	6,87	1,05	0,73
**SD**	0,61	0,85	0,06	0,09
Unpaired t-test vs control		0,01		0,01

D: CTP relative content

	Peak area	Peak area	mM in cells	mM in cells
**Metabolite**	**CTP**	**CTP**	**CTP**	**CTP**
**Extracellular Purine**	**None**	**Adenine**	**None**	**Adenine**
sample 1	2,70	1,79	0,31	0,21
sample 2	3,06	1,84	0,35	0,22
sample 3	2,85	1,82	0,33	0,22
**Mean**	2,87	1,82	0,33	0,22
**SD**	0,18	0,03	0,02	0,01
Unpaired t-test vs control		6,6E-03		6,6E-03

U-87 MG

E: ATP relative content

	Peak area	Peak area	mM in cells	mM in cells
**Metabolite**	**ATP**	**ATP**	**ATP**	**ATP**
**Extracellular Purine**	**None**	**Adenine**	**None**	**Adenine**
sample 1	30,09	43,10	3,95	5,66
sample 2	35,23	47,62	4,63	6,25
sample 3	29,49	41,75	3,87	5,48
sample 4	32,35	46,56	4,25	6,11
sample 5	34,08	47,82	4,48	6,28
**Mean**	32,25	45,37	4,24	5,96
**SD**	2,48	2,77	0,33	0,36
Unpaired t-test vs control		5,2E-05		5,2E-05

F: GTP relative content

	Peak area	Peak area	content	content
**Metabolite**	**GTP**	**GTP**	**GTP**	**GTP**
**Extracellular Purine**	**None**	**Adenine**	**None**	**Adenine**
sample 1	4,90	4,23	1,03	0,89
sample 2	5,12	4,42	1,07	0,93
sample 3	4,04	3,79	0,85	0,79
sample 4	4,59	4,25	0,96	0,89
sample 5	5,24	4,28	1,10	0,90
**Mean**	4,78	4,19	1,00	0,88
**SD**	0,48	0,24	0,10	0,05
Unpaired t-test vs control		0,05		0,05

G: UTP relative content

	Peak area	Peak area	content	content
**Metabolite**	**UTP**	**UTP**	**UTP**	**UTP**
**Extracellular Purine**	**None**	**Adenine**	**None**	**Adenine**
sample 1	2,95	1,58	0,79	0,42
sample 2	4,06	2,12	1,09	0,57
sample 3	3,48	1,93	0,93	0,52
sample 4	3,93	2,24	1,05	0,60
sample 5	4,28	2,25	1,14	0,60
**Mean**	3,74	2,02	1,00	0,54
**SD**	0,53	0,28	0,14	0,07
Unpaired t-test vs control		6,4E-04		6,4E-04

H: CTP relative content

	Peak area	Peak area	mM in cells	mM in cells
**Metabolite**	**CTP**	**CTP**	**CTP**	**CTP**
**Extracellular Purine**	**None**	**Adenine**	**None**	**Adenine**
sample 1	1,35	0,80	0,90	0,53
sample 2	1,60	0,90	1,06	0,60
sample 3	1,36	0,80	0,90	0,53
sample 4	1,52	0,88	1,01	0,59
sample 5	1,69	0,89	1,12	0,59
**Mean**	1,50	0,85	1,00	0,57
**SD**	0,15	0,05	0,10	0,03
Unpaired t-test vs control		2,6E-04		2,6E-04

Table S15: Raw data Figure S1 (Part 1/3)

A: [ATP] in mM

				mM	mM	mM
	µM in extract	µM in extract	µM in extract	in cells	in cells	in cells
**Metabolite**	**ATP**	**ATP**	**ATP**	**ATP**	**ATP**	**ATP**
**Strain**	**WT**	***prs1 prs3***	***prs2 prs4 prs5***	**WT**	***prs1 prs3***	***prs2 prs4 prs5***
sample 1	118,48	49,71	97,25	3,55	1,49	2,92
sample 2	117,40	47,30	96,23	3,52	1,42	2,89
sample 3	116,72	54,50	106,28	3,50	1,63	3,19
sample 4	121,18	55,48	96,35	3,64	1,66	2,89
sample 5	123,87	57,21	103,25	3,72	1,72	3,10
sample 6	121,42	52,97	103,75	3,64	1,59	3,11
sample 7	125,24	57,51	102,18	3,76	1,73	3,07
sample 8	126,49	54,25	102,28	3,79	1,63	3,07
sample 9	126,11	58,87	103,38	3,78	1,77	3,10
sample 10	121,90	56,09	100,27	3,66	1,68	3,01
**Mean**	121,88	54,39	101,12	3,66	1,63	3,03
**SD**	3,55	3,59	3,46	0,11	0,11	0,10
Unpaired t-test vs WT		1,8E-19	1,0E-10		1,8E-19	1,0E-10
Unpaired t-test vs *prs1 prs3*	1,8E-19		1,0E-16	1,8E-19		1,0E-16

B: [GTP] in mM

				mM	mM	mM
	µM in extract	µM in extract	µM in extract	in cells	in cells	in cells
**Metabolite**	**GTP**	**GTP**	**GTP**	**GTP**	**GTP**	**GTP**
**Strain**	**WT**	***prs1 prs3***	***prs2 prs4 prs5***	**WT**	***prs1 prs3***	***prs2 prs4 prs5***
sample 1	30,892	5,732	22,304	0,93	0,17	0,67
sample 2	31,656	5,599	21,904	0,95	0,17	0,66
sample 3	31,270	6,075	23,973	0,94	0,18	0,72
sample 4	31,391	6,595	21,800	0,94	0,20	0,65
sample 5	32,874	6,186	23,476	0,99	0,19	0,70
sample 6	32,874	6,063	22,830	0,99	0,18	0,68
sample 7	34,583	6,933	22,949	1,04	0,21	0,69
sample 8	33,254	6,998	23,461	1,00	0,21	0,70
sample 9	33,231	6,717	23,466	1,00	0,20	0,70
sample 10	32,854	6,476	23,650	0,99	0,19	0,71
**Mean**	32,488	6,337	22,981	0,97	0,19	0,69
**SD**	1,149	0,483	0,759	0,03	0,01	0,02
Unpaired t-test vs WT		7,4E-17	4,1E-13		7,4E-17	4,1E-13
Unpaired t-test vs *prs1 prs3*	7,4E-17		2,2E-19	7,4E-17		2,2E-19

C: UTP] in mM

				mM	mM	mM
	µM in extract	µM in extract	µM in extract	in cells	in cells	in cells
**Metabolite**	**UTP**	**UTP**	**UTP**	**UTP**	**UTP**	**UTP**
**Strain**	**WT**	***prs1 prs3***	***prs2 prs4 prs5***	**WT**	***prs1 prs3***	***prs2 prs4 prs5***
sample 1	37,980	14,949	34,040	1,14	0,45	1,02
sample 2	38,081	13,778	34,232	1,14	0,41	1,03
sample 3	37,768	14,461	36,399	1,13	0,43	1,09
sample 4	40,475	15,301	33,355	1,21	0,46	1,00
sample 5	39,535	14,727	35,860	1,19	0,44	1,08
sample 6	39,596	15,117	34,542	1,19	0,45	1,04
sample 7	42,230	16,486	36,031	1,27	0,49	1,08
sample 8	38,794	15,539	36,498	1,16	0,47	1,09
sample 9	40,037	16,225	36,253	1,20	0,49	1,09
sample 10	39,974	16,014	34,871	1,20	0,48	1,05
**Mean**	39,447	15,260	35,208	1,18	0,46	1,06
**SD**	1,363	0,838	1,134	0,04	0,03	0,03
Unpaired t-test vs WT		9,1E-18	6,7E-07		9,1E-18	6,7E-07
Unpaired t-test vs *prs1 prs3*	9,1E-18		1,0E-18	9,1E-18		1,0E-18

D: [CTP] in mM

				mM	mM	mM
	µM in extract	µM in extract	µM in extract	in cells	in cells	in cells
**Metabolite**	**CTP**	**CTP**	**CTP**	**CTP**	**CTP**	**CTP**
**Strain**	**WT**	***prs1 prs3***	***prs2 prs4 prs5***	**WT**	***prs1 prs3***	***prs2 prs4 prs5***
sample 1	22,458	8,082	18,425	0,67	0,24	0,55
sample 2	22,597	7,789	18,437	0,68	0,23	0,55
sample 3	22,276	8,895	19,930	0,67	0,27	0,60
sample 4	24,490	9,957	19,915	0,73	0,30	0,60
sample 5	24,305	9,115	19,974	0,73	0,27	0,60
sample 6	23,910	8,928	18,816	0,72	0,27	0,56
sample 7	24,795	9,891	19,299	0,74	0,30	0,58
sample 8	23,423	9,203	19,240	0,70	0,28	0,58
sample 9	24,558	9,851	19,750	0,74	0,30	0,59
sample 10	24,391	9,544	18,332	0,73	0,29	0,55
**Mean**	23,720	9,125	19,212	0,71	0,27	0,58
**SD**	0,961	0,742	0,670	0,03	0,02	0,02
Unpaired t-test vs WT		7,9E-18	1,6E-09		7,9E-18	1,6E-09
Unpaired t-test vs *prs1 prs3*	7,9E-18		3,6E-17	7,9E-18		3,6E-17

E: [ADP] in mM

				mM	mM	mM
	µM in extract	µM in extract	µM in extract	in cells	in cells	in cells
**Metabolite**	**ADP**	**ADP**	**ADP**	**ADP**	**ADP**	**ADP**
**Strain**	**WT**	***prs1 prs3***	***prs2 prs4 prs5***	**WT**	***prs1 prs3***	***prs2 prs4 prs5***
sample 1	12,77	10,26	11,92	0,38	0,31	0,36
sample 2	11,92	9,96	13,12	0,36	0,30	0,39
sample 3	12,57	10,87	12,21	0,38	0,33	0,37
sample 4	12,97	11,32	12,20	0,39	0,34	0,37
sample 5	13,75	11,68	13,75	0,41	0,35	0,41
sample 6	14,27	11,86	12,74	0,43	0,36	0,38
sample 7	14,58	11,89	12,52	0,44	0,36	0,38
sample 8	14,51	11,63	13,61	0,44	0,35	0,41
sample 9	14,11	11,72	14,22	0,42	0,35	0,43
sample 10	13,33	11,36	13,89	0,40	0,34	0,42
**Mean**	13,478	11,255	13,019	0,40	0,34	0,39
**SD**	0,908	0,679	0,814	0,03	0,02	0,02
Unpaired t-test vs WT		1,1E-05	2,5E-01		1,1E-05	2,5E-01
Unpaired t-test vs *prs1 prs3*	1,1E-05		5,9E-05	1,1E-05		5,9E-05

Table S15: Raw data Figure S1 (Part 2/3)

F: [GDP] in mM

				mM	mM	mM
	µM in extract	µM in extract	µM in extract	in cells	in cells	in cells
**Metabolite**	**GDP**	**GDP**	**GDP**	**GDP**	**GDP**	**GDP**
**Strain**	**WT**	***prs1 prs3***	***prs2 prs4 prs5***	**WT**	***prs1 prs3***	***prs2 prs4 prs5***
sample 1	5,744	2,318	4,827	0,172	0,070	0,145
sample 2	5,301	2,224	4,758	0,159	0,067	0,143
sample 3	5,535	2,509	4,921	0,166	0,075	0,148
sample 4	5,441	2,362	4,066	0,163	0,071	0,122
sample 5	5,751	2,270	4,962	0,173	0,068	0,149
sample 6	5,343	2,330	4,615	0,160	0,070	0,138
sample 7	5,458	2,833	4,532	0,164	0,085	0,136
sample 8	5,325	2,398	4,657	0,160	0,072	0,140
sample 9	5,753	2,408	4,970	0,173	0,072	0,149
sample 10	5,403	2,515	4,784	0,162	0,075	0,144
**Mean**	5,505	2,417	4,709	0,165	0,072	0,141
**SD**	0,182	0,173	0,270	0,005	0,005	0,008
Unpaired t-test vs WT		8,6E-19	9,3E-07		8,6E-19	9,3E-07
Unpaired t-test vs *prs1 prs3*	8,6E-19		3,3E-13	8,6E-19		3,3E-13

G: [UDP] in mM

				mM	mM	mM
	µM in extract	µM in extract	µM in extract	in cells	in cells	in cells
**Metabolite**	**UDP**	**UDP**	**UDP**	**UDP**	**UDP**	**UDP**
**Strain**	**WT**	***prs1 prs3***	***prs2 prs4 prs5***	**WT**	***prs1 prs3***	***prs2 prs4 prs5***
sample 1	2,261	2,856	2,036	0,068	0,086	0,061
sample 2	1,972	2,649	1,824	0,059	0,079	0,055
sample 3	1,841	2,322	1,905	0,055	0,070	0,057
sample 4	2,655	3,053	1,634	0,080	0,092	0,049
sample 5	2,662	3,159	2,140	0,080	0,095	0,064
sample 6	2,387	2,780	1,481	0,072	0,083	0,044
sample 7	2,815	2,864	1,980	0,084	0,086	0,059
sample 8	2,866	2,720	1,937	0,086	0,082	0,058
sample 9	2,431	3,033	1,723	0,073	0,091	0,052
sample 10	2,493	2,789	1,899	0,075	0,084	0,057
**Mean**	2,438	2,823	1,856	0,073	0,085	0,056
**SD**	0,339	0,237	0,197	0,010	0,007	0,006
Unpaired t-test vs WT		9,6E-03	3,1E-04		9,6E-03	3,1E-04
Unpaired t-test vs *prs1 prs3*	9,6E-03		1,4E-08	9,6E-03		1,4E-08

H: [CDP] in mM

				mM	mM	mM
	µM in extract	µM in extract	µM in extract	in cells	in cells	in cells
**Metabolite**	**CDP**	**CDP**	**CDP**	**CDP**	**CDP**	**CDP**
**Strain**	**WT**	***prs1 prs3***	***prs2 prs4 prs5***	**WT**	***prs1 prs3***	***prs2 prs4 prs5***
sample 1	1,182	0,815	0,863	0,035	0,024	0,026
sample 2	1,211	0,820	0,983	0,036	0,025	0,029
sample 3	1,132	0,717	1,019	0,034	0,021	0,031
sample 4	1,287	0,766	0,867	0,039	0,023	0,026
sample 5	1,223	0,698	0,920	0,037	0,021	0,028
sample 6	1,163	0,746	0,833	0,035	0,022	0,025
sample 7	1,155	0,737	0,906	0,035	0,022	0,027
sample 8	1,372	0,735	1,019	0,041	0,022	0,031
sample 9	1,133	0,824	0,950	0,034	0,025	0,028
sample 10	1,265	0,753	1,071	0,038	0,023	0,032
**Mean**	1,212	0,761	0,943	0,036	0,023	0,028
**SD**	0,077	0,045	0,079	0,002	0,001	0,002
Unpaired t-test vs WT		1,3E-10	4,0E-07		1,3E-10	4,0E-07
Unpaired t-test vs *prs1 prs3*	1,3E-10		1,6E-05	1,3E-10		1,6E-05

I: [AMP] in mM

				mM	mM	mM
	µM in extract	µM in extract	µM in extract	in cells	in cells	in cells
**Metabolite**	**AMP**	**AMP**	**AMP**	**AMP**	**AMP**	**AMP**
**Strain**	**WT**	***prs1 prs3***	***prs2 prs4 prs5***	**WT**	***prs1 prs3***	***prs2 prs4 prs5***
sample 1	4,619	3,320	4,217	0,139	0,100	0,127
sample 2	4,227	3,456	3,897	0,127	0,104	0,117
sample 3	4,624	3,303	4,962	0,139	0,099	0,149
sample 4	3,950	3,747	3,947	0,119	0,112	0,118
sample 5	4,302	3,654	4,582	0,129	0,110	0,137
sample 6	4,516	3,792	4,112	0,135	0,114	0,123
sample 7	4,397	3,785	4,428	0,132	0,114	0,133
sample 8	4,222	3,647	3,815	0,127	0,109	0,114
sample 9	4,299	3,424	4,425	0,129	0,103	0,133
sample 10	4,456	3,728	4,284	0,111	0,093	0,107
**Mean**	4,361	3,586	4,267	0,129	0,106	0,126
**SD**	0,207	0,192	0,350	0,009	0,007	0,012
Unpaired t-test vs WT		7,6E-08	4,8E-01		4,9E-06	5,7E-01
Unpaired t-test vs *prs1 prs3*	7,6E-08		9,5E-05	4,9E-06		5,1E-04

J: [GMP] in mM

				mM	mM	mM
	µM in extract	µM in extract	µM in extract	in cells	in cells	in cells
**Metabolite**	**GMP**	**GMP**	**GMP**	**GMP**	**GMP**	**GMP**
**Strain**	**WT**	***prs1 prs3***	***prs2 prs4 prs5***	**WT**	***prs1 prs3***	***prs2 prs4 prs5***
sample 1	12,831	2,837	10,141	0,385	0,085	0,304
sample 2	12,555	2,908	10,263	0,377	0,087	0,308
sample 3	10,503	2,585	10,591	0,315	0,078	0,318
sample 4	11,098	2,866	9,832	0,333	0,086	0,295
sample 5	12,545	2,908	10,729	0,376	0,087	0,322
sample 6	12,538	2,906	10,892	0,376	0,087	0,327
sample 7	11,054	3,049	10,117	0,332	0,091	0,304
sample 8	11,255	3,014	9,816	0,338	0,090	0,294
sample 9	12,461	2,969	10,488	0,374	0,089	0,315
sample 10	11,876	2,984	9,541	0,356	0,090	0,286
**Mean**	11,872	2,903	10,241	0,356	0,087	0,307
**SD**	0,827	0,130	0,436	0,025	0,004	0,013
Unpaired t-test vs WT		3,5E-11	8,3E-05		3,5E-11	8,3E-05
Unpaired t-test vs *prs1 prs3*	3,5E-11		5,2E-14	3,5E-11		5,2E-14

Table S15: Raw data Figure S1 (Part 3/3)

K: [UMP] in mM

				mM	mM	mM
	**µM in extract**	**µM in extract**	**µM in extract**	**in cells**	**in cells**	**in cells**
**Metabolite**	**UMP**	**UMP**	**UMP**	**UMP**	**UMP**	**UMP**
**Strain**	**WT**	***prs1 prs3***	***prs2 prs4 prs5***	**WT**	***prs1 prs3***	***prs2 prs4 prs5***
sample 1	15,799	15,168	12,120	0,474	0,455	0,364
sample 2	15,144	15,754	13,760	0,454	0,473	0,413
sample 3	15,190	16,945	14,321	0,456	0,508	0,430
sample 4	14,553	16,958	12,465	0,437	0,509	0,374
sample 5	16,325	17,331	13,373	0,490	0,520	0,401
sample 6	16,460	16,376	13,647	0,494	0,491	0,409
sample 7	15,355	17,389	12,137	0,461	0,522	0,364
sample 8	15,023	16,925	11,718	0,451	0,508	0,352
sample 9	16,731	17,874	12,881	0,502	0,536	0,386
sample 10	15,825	16,675	11,559	0,475	0,500	0,347
**Mean**	15,641	16,739	12,798	0,469	0,502	0,384
**SD**	0,706	0,800	0,944	0,021	0,024	0,028
Unpaired t-test vs WT		4,4E-03	7,9E-07		4,4E-03	7,9E-07
Unpaired t-test vs *prs1 prs3*	4,4E-03		1,0E-08	4,4E-03		1,0E-08

L: [CMP] in mM

				mM	mM	mM
	**µM in extract**	**µM in extract**	**µM in extract**	**in cells**	**in cells**	**in cells**
**Metabolite**	**CMP**	**CMP**	**CMP**	**CMP**	**CMP**	**CMP**
**Strain**	**WT**	***prs1 prs3***	***prs2 prs4 prs5***	**WT**	***prs1 prs3***	***prs2 prs4 prs5***
sample 1	0,572	0,371	0,434	0,017	0,011	0,013
sample 2	0,546	0,391	0,454	0,016	0,012	0,014
sample 3	0,511	0,337	0,413	0,015	0,010	0,012
sample 4	0,509	0,471	0,404	0,015	0,014	0,012
sample 5	0,654	0,449	0,459	0,020	0,013	0,014
sample 6	0,496	0,463	0,384	0,015	0,014	0,012
sample 7	0,466	0,417	0,461	0,014	0,013	0,014
sample 8	0,535	0,402	0,420	0,016	0,012	0,013
sample 9	0,461	0,471	0,404	0,014	0,014	0,012
sample 10	0,479	0,400	0,384	0,014	0,012	0,012
**Mean**	0,523	0,417	0,422	0,016	0,013	0,013
**SD**	0,058	0,046	0,029	0,002	0,001	0,001
Unpaired t-test vs WT		2,9E-04	2,5E-04		2,9E-04	2,5E-04
Unpaired t-test vs *prs1 prs3*	2,9E-04		8,0E-01	2,9E-04		8,0E-01

Table S16 Figure S3

B: [ATP] in mM

	mM	mM	mM	mM
	in cells	in cells	in cells	in cells
**Metabolite (mM)**	**[ATP]**	**[ATP]**	**[ATP]**	**[ATP]**
**Strain**	***URA2* - uracil**	***URA2* + uracil**	***ura2Δ* - uracil**	***ura2Δ* + uracil**
sample 1	3,064	3,353	3,994	3,072
sample 2	3,490	2,811	4,210	2,869
sample 3	3,020	3,036	3,820	3,223
sample 4	3,409	3,292	4,270	3,527
**Mean**	3,25	3,12	4,07	3,17
**SD**	0,24	0,25	0,21	0,28
Unpaired t-test vs *URA2* -uracil		0,50	2,0E-03	0,70

C: [GTP] in mM

	mM	mM	mM	mM
	in cells	in cells	in cells	in cells
**Metabolite (mM)**	**[GTP]**	**[GTP]**	**[GTP]**	**[GTP]**
**Strain**	***URA2* - uracil**	***URA2* + uracil**	***ura2Δ* - uracil**	***ura2Δ* + uracil**
sample 1	0,795	0,820	0,976	0,806
sample 2	0,797	0,738	1,063	0,802
sample 3	0,823	0,723	0,984	0,870
sample 4	0,782	0,756	0,973	0,848
**Mean**	0,80	0,76	1,00	0,83
**SD**	0,02	0,04	0,04	0,03
Unpaired t-test vs *URA2* -uracil		0,16	1,0E-03	0,15

D: [UTP] in mM

	mM	mM	mM	mM
	in cells	in cells	in cells	in cells
**Metabolite (mM)**	**[UTP]**	**[UTP]**	**[UTP]**	**[UTP]**
**Strain**	***URA2* - uracil**	***URA2* + uracil**	***ura2Δ* - uracil**	***ura2Δ* + uracil**
sample 1	0,709	1,025	0,010	0,795
sample 2	0,809	0,943	0,003	0,813
sample 3	0,805	1,095	0,004	0,931
sample 4	0,880	1,126	0,015	0,858
**Mean**	0,88	1,13	0,02	0,86
**SD**	0,07	0,08	0,01	0,06
Unpaired t-test vs *URA2* -uracil		3,9E-03	1,7E-04	0,34

E: [CTP] in mM

	mM	mM	mM	mM
	in cells	in cells	in cells	in cells
**Metabolite (mM)**	**[CTP]**	**[CTP]**	**[CTP]**	**[CTP]**
**Strain**	***URA2* - uracil**	***URA2* + uracil**	***ura2Δ* - uracil**	***ura2Δ* + uracil**
sample 1	0,446	0,532	0,186	0,453
sample 2	0,442	0,477	0,226	0,451
sample 3	0,461	0,476	0,192	0,466
sample 4	0,448	0,560	0,216	0,530
**Mean**	0,88	1,13	0,02	0,86
**SD**	0,01	0,04	0,02	0,04
Unpaired t-test vs *URA2* -uracil		0,06	1,6E-05	0,26

F: [NAD^+ + NADH] in mM

	mM	mM	mM	mM
	in cells	in cells	in cells	in cells
**Metabolite (mM)**	**[NAD(H)]**	**[NAD(H)]**	**[NAD(H)]**	**[NAD(H)]**
**Strain**	***URA2* - uracil**	***URA2* + uracil**	***ura2Δ* - uracil**	***ura2Δ* + uracil**
sample 1	2,152	2,298	2,789	2,377
sample 2	2,289	2,298	3,051	2,256
sample 3	2,290	2,316	2,852	2,534
sample 4	2,252	2,528	2,783	2,355
**Mean**	0,88	1,13	0,02	0,86
**SD**	0,07	0,11	0,13	0,12
Unpaired t-test vs *URA2* -uracil		0,14	5,2E-04	0,10

Table S17: Raw data Figure S4

A: [PRPP] in µM

	Peak area	Peak area	Peak area	Peak area	µM in cells	µM in cells	µM in cells	µM in cells
**Metabolite**	PRPP	PRPP	PRPP	PRPP	PRPP	PRPP	PRPP	PRPP
**Strain**	Control	PRPS1 WT	PRPS1 V142L	PRPS1 A190V	Control	PRPS1 WT	PRPS1 V142L	PRPS1 A190V
sample 1	0,00045	0,00040	0,00120	0,00080	6,6651	5,9245	17,7736	11,8491
sample 2	0,00032	0,00021	0,00096	0,00085	4,7259	3,1506	14,1778	12,6025
sample 3	0,00042	0,00042	0,00084	0,00063	6,2537	6,2537	12,5073	9,3805
sample 4	0,00026	0,00010	0,00114	0,00073	3,8443	1,5377	16,9148	10,7639
**Mean**	0,0004	0,0003	0,0010	0,0008	5,3722	4,2166	15,3434	11,1490
**SD**	0,0001	0,0002	0,0002	0,0001	1,3167	2,2642	2,4343	1,3998
Unpaired t-test vs control		0,42	1,1E-03	9,7E-04		0,42	1,1E-03	9,7E-04

B: [ATP] in mM

	Peak area	Peak area	Peak area	Peak area	mM in cells	mM in cells	mM in cells	mM in cells
**Metabolite**	ATP	ATP	ATP	ATP	PRPP	PRPP	PRPP	PRPP
**Strain**	Control	PRPS1 WT	PRPS1 V142L	PRPS1 A190V	Control	PRPS1 WT	PRPS1 V142L	PRPS1 A190V
sample 1	6,398	6,726	9,175	10,075	3,48	3,66	5,00	5,49
sample 2	6,865	7,131	9,632	10,392	3,74	3,88	5,24	5,66
sample 3	6,760	7,110	9,888	10,224	3,68	3,87	5,38	5,57
sample 4	6,376	6,677	9,845	9,511	3,47	3,64	5,36	5,18
**Mean**	6,60	6,91	9,64	10,05	3,59	3,76	5,25	5,47
**SD**	0,25	0,24	0,33	0,38	0,14	0,13	0,18	0,21
Unpaired t-test vs control		0,12	1,0E-05	1,8E-05		0,12	1,0E-05	1,8E-05

C: [GTP] in mM

	Peak area	Peak area	Peak area	Peak area	mM in cells	mM in cells	mM in cells	mM in cells
**Metabolite**	GTP	GTP	GTP	GTP	GTP	GTP	GTP	GTP
**Strain**	Control	PRPS1 WT	PRPS1 V142L	PRPS1 A190V	Control	PRPS1 WT	PRPS1 V142L	PRPS1 A190V
sample 1	0,743	0,810	1,119	1,182	0,55	0,60	0,83	0,87
sample 2	0,790	0,855	1,142	1,248	0,58	0,63	0,84	0,92
sample 3	0,782	0,856	1,171	1,216	0,58	0,63	0,87	0,90
sample 4	0,731	0,773	1,079	1,203	0,54	0,57	0,80	0,89
**Mean**	0,76	0,82	1,13	1,21	0,56	0,61	0,83	0,90
**SD**	0,03	0,04	0,04	0,03	0,02	0,03	0,03	0,02
Unpaired t-test vs control		0,05	1,1E-05	4,8E-07		0,05	1,1E-05	4,8E-07

D: [UTP] in mM

	Peak area	Peak area	Peak area	Peak area	mM in cells	mM in cells	mM in cells	mM in cells
**Metabolite**	UTP	UTP	UTP	UTP	UTP	UTP	UTP	UTP
**Strain**	Control	PRPS1 WT	PRPS1 V142L	PRPS1 A190V	Control	PRPS1 WT	PRPS1 V142L	PRPS1 A190V
sample 1	0,788	0,773	1,557	1,435	0,88	0,87	1,75	1,61
sample 2	0,813	0,846	1,432	1,463	0,91	0,95	1,61	1,64
sample 3	0,825	0,850	1,461	1,442	0,93	0,95	1,64	1,62
sample 4	0,773	0,852	1,468	1,324	0,87	0,96	1,65	1,49
**Mean**	0,80	0,83	1,48	1,42	0,90	0,93	1,66	1,59
**SD**	0,02	0,04	0,05	0,06	0,03	0,04	0,06	0,07
Unpaired t-test vs control		0,24	1,6E-05	6,8E-05		0,24	1,6E-05	6,8E-05

E: [CTP] in mM

	Peak area	Peak area	Peak area	Peak area	mM in cells	mM in cells	mM in cells	mM in cells
**Metabolite**	CTP	CTP	CTP	CTP	CTP	CTP	CTP	CTP
**Strain**	Control	PRPS1 WT	PRPS1 V142L	PRPS1 A190V	Control	PRPS1 WT	PRPS1 V142L	PRPS1 A190V
sample 1	0,319	0,329	0,491	0,544	0,40	0,41	0,61	0,68
sample 2	0,344	0,359	0,551	0,578	0,43	0,45	0,69	0,72
sample 3	0,329	0,353	0,547	0,558	0,41	0,44	0,68	0,70
sample 4	0,297	0,325	0,539	0,504	0,37	0,41	0,67	0,63
**Mean**	0,32	0,34	0,53	0,55	0,40	0,43	0,66	0,68
**SD**	0,02	0,02	0,03	0,03	0,02	0,02	0,03	0,04
Unpaired t-test vs control		0,19	3,7E-05	6,5E-05		0,19	3,7E-05	6,5E-05

F: [NAD(H)] in mM

	µM in sample	µM in sample	µM in sample	µM in sample	mM in cells	mM in cells	mM in cells	mM in cells
**Metabolite**	NAD(H)	NAD(H)	NAD(H)	NAD(H)	NAD(H)	NAD(H)	NAD(H)	NAD(H)
**Strain**	Control	PRPS1 WT	PRPS1 V142L	PRPS1 A190V	Control	PRPS1 WT	PRPS1 V142L	PRPS1 A190V
sample 1	1,182	1,192	1,788	1,350	0,263	0,265	0,397	0,300
sample 2	0,998	1,253	1,983	1,399	0,236	0,296	0,469	0,331
sample 3	0,961	1,314	1,667	1,192	0,225	0,308	0,391	0,280
sample 4	1,156	1,010	1,509	1,484	0,267	0,233	0,348	0,342
**Mean**	1,07	1,19	1,74	1,36	0,248	0,275	0,401	0,313
**SD**	0,11	0,13	0,20	0,12	0,020	0,034	0,050	0,029
Unpaired t-test vs control		0,22	4,9E-03	0,01		0,22	4,9E-03	0,01

G: Cell proliferation

	Cell/cm² $x10^{-3}$	Cell/cm² $x10^{-3}$	Cell/cm² $x10^{-3}$	Cell/cm² $x10^{-3}$
	Control	PRPS1 WT	PRPS1 V142L	PRPS1 A190V
Strain 1	227,1	254,5	128,3	119,1
Strain 2	215,2	245,8	133,3	119,9
Strain 3	216,4	244,7	123,2	106,7
Unpaired t-test vs control		4,7E-03	7,1E-05	5,8E-05
